# Mechanochemical Crosstalk Produces Cell-Intrinsic Patterning of the Cortex to Orient the Mitotic Spindle

**DOI:** 10.1016/j.cub.2020.06.098

**Published:** 2020-09-21

**Authors:** Andrea Dimitracopoulos, Pragya Srivastava, Agathe Chaigne, Zaw Win, Roie Shlomovitz, Oscar M. Lancaster, Maël Le Berre, Matthieu Piel, Kristian Franze, Guillaume Salbreux, Buzz Baum

**Affiliations:** 1 Department of Physiology, Development and Neuroscience, University of Cambridge, Downing Street, Cambridge CB2 3DY, UK; 2 MRC Laboratory for Molecular Cell Biology, University College London, Gower Street, London WC1E 6BT, UK; 3 The Francis Crick Institute, 1 Midland Road, London NW1 1AT, UK; 4 Department of Chemical Physics, The Weizmann Institute of Science, PO Box 26, Rehovot 76100, Israel; 5 Institut Curie, PSL Research University, CNRS, UMR 144, Paris 75005, France; 6 Institute for the Physics of Living Systems, University College London, Gower Street, London WC1E 6BT, UK

**Keywords:** mitotic spindle, computational model, spindle orientation, mitotic rounding, mechanochemical model, microtubules, Ran-GTP, LGN, cell, long axis, monopolar spindle

## Abstract

Proliferating animal cells are able to orient their mitotic spindles along their interphase cell axis, setting up the axis of cell division, despite rounding up as they enter mitosis. This has previously been attributed to molecular memory and, more specifically, to the maintenance of adhesions and retraction fibers in mitosis [1, 2, 3, 4, 5, 6], which are thought to act as local cues that pattern cortical Gαi, LGN, and nuclear mitotic apparatus protein (NuMA) [3, 7, 8, 9, 10, 11, 12, 13, 14, 15, 16, 17, 18]. This cortical machinery then recruits and activates Dynein motors, which pull on astral microtubules to position the mitotic spindle. Here, we reveal a dynamic two-way crosstalk between the spindle and cortical motor complexes that depends on a Ran-guanosine triphosphate (GTP) signal [12], which is sufficient to drive continuous monopolar spindle motion independently of adhesive cues in flattened human cells in culture. Building on previous work [1, 12, 19, 20, 21, 22, 23], we implemented a physical model of the system that recapitulates the observed spindle-cortex interactions. Strikingly, when this model was used to study spindle dynamics in cells entering mitosis, the chromatin-based signal was found to preferentially clear force generators from the short cell axis, so that cortical motors pulling on astral microtubules align bipolar spindles with the interphase long cell axis, without requiring a fixed cue or a physical memory of interphase shape. Thus, our analysis shows that the ability of chromatin to pattern the cortex during the process of mitotic rounding is sufficient to translate interphase shape into a cortical pattern that can be read by the spindle, which then guides the axis of cell division.

## Highlights

• Monopolar spindles undergo persistent chase and run movements in flat mitotic cells
• Spindle movements depend on microtubules, LGN, and the Ran-GTP pathway
• A mechanochemical computational model recapitulates monopolar spindle movements
• The model shows how spindles orient along the long cell axis during mitotic rounding

## Teaser

Dimitracopoulos et al. show how two-way feedback between the cortex and the spindle drives unstable spindle movements in mitotic cells in culture and show how this dynamic system is able to orient the mitotic spindle so that it aligns with the interphase long cell axis, even in the absence of external cues.

## Results and Discussion

**Figure 1:**
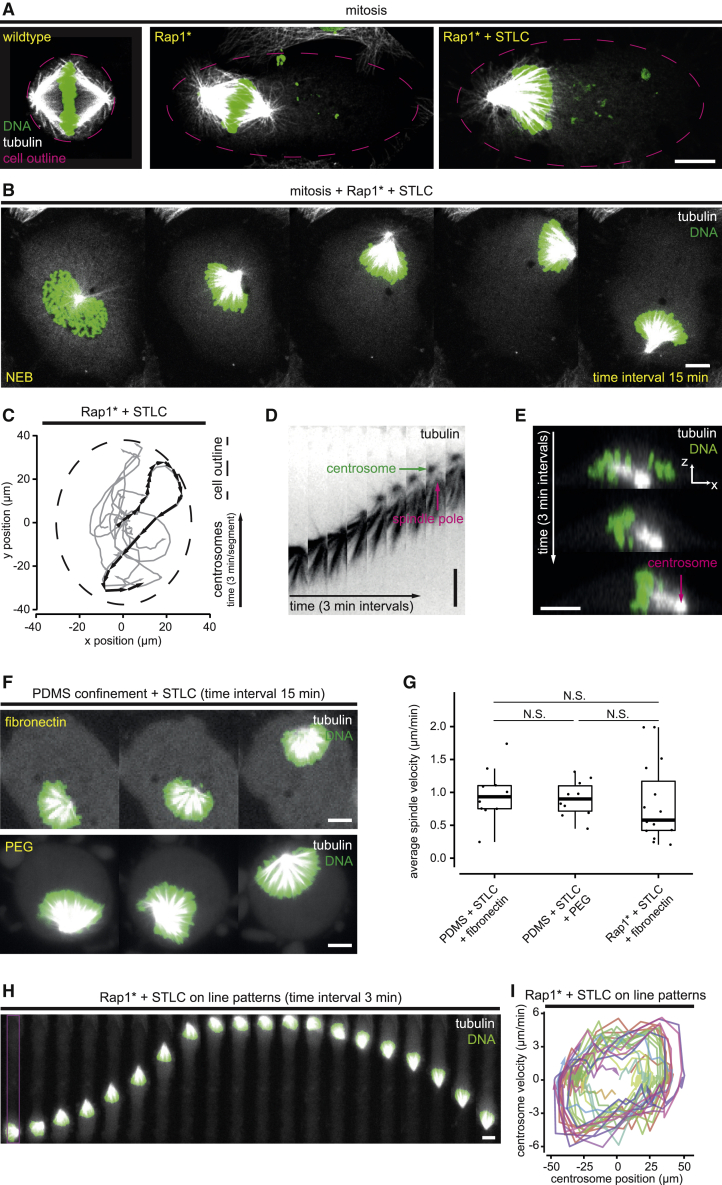
Flat cells with a monopolar spindle as a simplified system to study dynamic spindle positioning (A) Immuno-fluorescence confocal images of HeLa cells in mitosis on FN-coated unpatterned substrates. Wild-type cells are round and have a bipolar spindle (left). Overexpression of Rap1^∗^ results in cells that fail to round up in mitosis (middle), and the combined treatment of Rap1^∗^ and STLC results in flat mitotic cells with a monopolar spindle. The dashed line (magenta) shows the elliptical fit of the cell outline. (B) Time-lapse confocal images of a HeLa cell (Rap1^∗^ + STLC) on a uniformly FN-coated substrate as it enters mitosis: at NEB, the two centrosomes fail to separate, resulting in monopolar spindle formation. The monopolar spindle moves freely and continuously. (C) Plot of the trajectories of monopolar spindles in mitotic HeLa cells on fibronectin-coated adhesive substrates (Rap1^∗^ + STLC; n = 15). The trajectory of the spindle shown in (B) is highlighted in black, and the outline of the cell is shown as a dashed line, although other trajectories are shown in gray. (D) Detailed time lapse of the monopolar spindle shown in (B). The centrosome leads the movement, and the rest of the spindle follows. (E) X-Z section of a confocal time lapse of a representative HeLa cell treated with Rap1^∗^ + STLC on a FN-coated unpatterned substrate. Centrosomes lie close to the basal membrane as the spindle moves. (F) Spindle motion still occurs in STLC-treated HeLa cells on FN-coated surfaces under a FN-coated PDMS roof (upper graphic, top row; n = 10), as well as on non-adherent PEG-coated surfaces, held flat under a PEG-coated roof of PDMS (bottom; n = 10). (G) Boxplots of STLC-treated monopolar spindle velocities in cells flattened by different means. Physical confinement with FN or PEG coating (shown in F) or genetic treatments (Rap1^∗^ overexpression; shown in B and C) results in similar monopolar spindle behavior (PDMS + STLC + FN n = 10; PDMS + STLC + PEG n = 10; Rap1* + STLC + FN n = 15; significance tests: p > 0.05; Mann-Whitney U test). Thick bars and boxes indicate median values and lower/upper quartiles, respectively. Whiskers extend to the smallest/largest value, but no further than 1.5 times the interquartile range. (H) Wide-field time-lapse images of a mitotic HeLa cell (Rap1^∗^ + STLC) on a FN-coated line pattern (10 μm width, magenta box). The spindle follows a 1D path, alternating its direction of movement. (I) Phase portrait of centrosome motion in monopolar cells on line-patterns (as in H). The phase portrait reveals that the spindle motion alternates between fast motion and pausing near cell ends as it changes direction. All cells in time-lapse images are expressing tubulin-GFP and H2B-mCherry. All scale bars indicate 10 μm. See also Figure S1 and Video S1A.

Mitosis in animal cells is accompanied by large-scale changes in cell geometry and cytoskeletal organization, making it hard to understand how the spindle in a rounded mitotic cell is able to align along the interphase long cell axis. Thus, to simplify the system, we overexpressed a constitutively activated form of the small guanosine triphosphatase (GTPase) Rap1 to prevent mitotic rounding (Rap1^∗^ in this text) [24, 25] in the presence/absence of a kinesin-5 inhibitor S-trityl-L-cysteine (STLC), which both inhibits the formation of a bipolar spindle (Figures 1 and S1A–S1D) [26] and stops cells from exiting mitosis, extending the time window in which to study mitotic cells (Figures 1A–1C; Video S1A) [26]. When cells were treated in this way and imaged by using tubulin-GFP and H2B-mCherry, we observed striking spindle dynamics in flat monopolar cells (Figures S1E–S1I). In 13 out of 15 of Rap1^∗^ cells treated with STLC, spindles moved more than 10 μm away from their average position (Figures 1B and S1I), traveling at speeds of up to ∼5 μm/min (Figure S1H). As spindles moved across the basal cell cortex, centrosomes led, tilted downward (Figures 1C–1E), while kinetochore-microtubules and DNA followed (Figures 1B and 1D).

**Figure 2:**
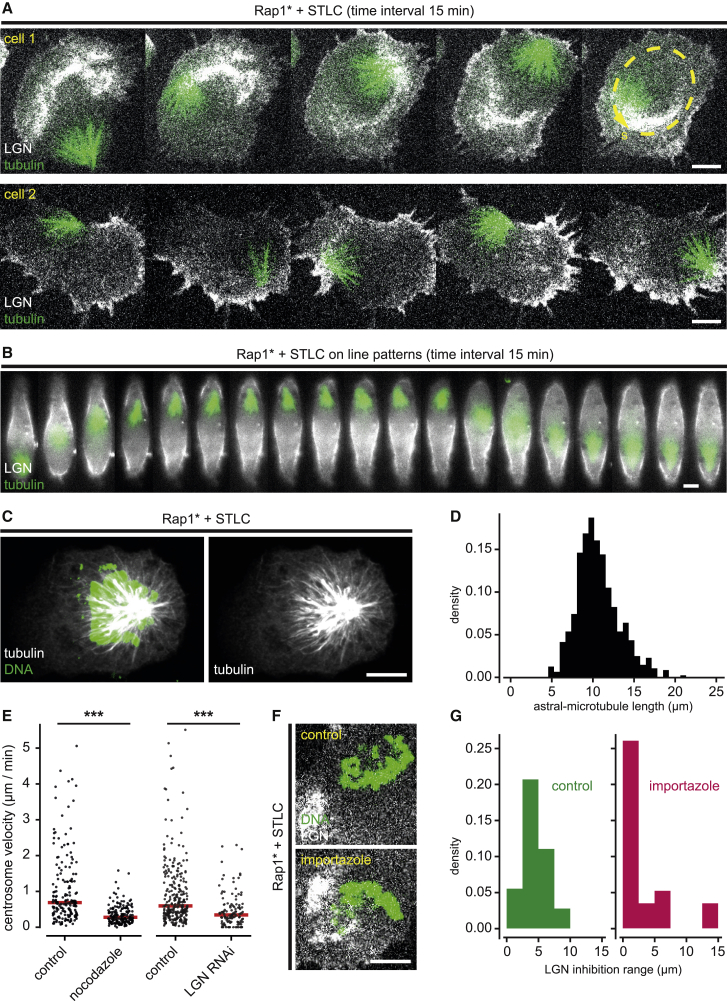
LGN, astral microtubules, and the ran pathway are responsible for monopolar spindle motion in flat cells (A and B) Time-lapse imaging reveals a feedback loop between spindle position and cortical LGN levels. LGN levels decrease in regions close to the spindle and increase far from the spindle. (A) Time-lapse images of mitotic HeLa cells (Rap1^∗^ + STLC) on FN-coated unpatterned substrates (confocal imaging; two-representative example). The last frame of the top montage shows the reference line used to obtain the kymograph in Figure 3B. (B) Time-lapse images of a mitotic HeLa cell (Rap1^∗^ + STLC) on a FN-coated line pattern (wide-field imaging, representative example). (C) Maximum projection of immuno-fluorescence confocal images of a HeLa cell (Rap1^∗^ + STLC) in mitosis on a FN-coated unpatterned substrate. Microtubules emanate radially from the spindle pole. (D) Histogram of astral microtubule lengths, measured as the distance between the spindle pole and the microtubule ends in HeLa cells (as shown in C; n = 8). (E) Perturbing astral microtubules or LGN reduces spindle velocity. Plots show centrosome velocities in control HeLa cells (Rap1^∗^ + STLC), in cells treated with small doses of nocodazole to perturb astral microtubules (control n = 9; nocodazole n = 9), and in LGN RNAi cells (control n = 14; LGN RNAi n = 6; significance tests: p < 0.001; Mann-Whitney U test). Red lines indicate median values (F and G) Perturbation of the Ran pathway reduces LGN clearance close to the DNA. (F) Confocal images of mitotic HeLa cells (Rap1^∗^ + STLC) on FN-coated unpatterned substrates, treated with importazole to perturb the Ran pathway. Compared with control, importazole-treated cells show higher levels of LGN near the DNA. (G) Histograms show minimum distance between DNA and high-level LGN (thresholded at the 0.975 quantile) in control (n = 29) and importazole-treated (n = 23) HeLa cells (Rap1^∗^ + STLC). In importazole-treated cells, the LGN inhibition range is shorter than in control cells (medians: 4.5 μm control; 1.2 μm importazole treated; p < 0.01; Mann-Whitney *U* test). Images of live cells show GFP-LGN, tubulin-mCherry, and/or H2B-mCherry. All scale bars indicate 10 μm. See also Figure S2 and Videos S1B and S1C.

Video S1. Simplified experimental system and mechanochemical model to study dynamic spindle positioning, related to Figures 1, 2, and 4 A – Monopolar spindles move freely and continuously in flat cells. Time-lapse confocal images of a HeLa cell (Rap1^∗^ + STLC) labeled with tubulin-GFP (gray) and H2B-mCherry (green) entering mitosis, from 9 min before NEB onwards. B – Monopolar spindles in flat cells reveal a feedback loop between spindle position and cortical LGN levels. Time-lapse confocal images of a HeLa cell (Rap1^∗^ + STLC) labeled with GFP-LGN (gray) and tubulin-mCherry (green) in mitosis. C – Two half spindles move in a Rap1^∗^ + STLC-treated cell. Time-lapse confocal images of a HeLa cell (Rap1^∗^ + STLC) labeled with GFP-LGN (gray) and tubulin-mCherry (green) in mitosis, where a transiently formed bipolar spindle spontaneously breaks into two monopolar spindles. D – Bipolar spindle formation and reorientation around NEB. Time-lapse confocal images of a HeLa cell labeled with GFP-LGN (gray) and tubulin-mCherry (green) in mitosis. E – Simulation of mitotic rounding and spindle rotation in control conditions. Cell shape, DNA and spindle angle, and LGN concentration at different times after NEB, in a simulation of mitotic rounding and spindle rotation in control conditions, for an initial spindle angle$\;\varphi_{0}=\pi /4$. F – Simulation of mitotic rounding and spindle rotation in LGN-RNAi conditions. Cell shape, DNA and spindle angle, and LGN concentration at different times after NEB, in a simulation of mitotic rounding and spindle rotation in LGN-RNAi conditions, for an initial angle $\;\varphi_{0}=\pi /4$.

We were then able to use this simplified system to test whether cell-extracellular matrix (ECM) adhesions function as positional cues to guide spindle movements, as previously proposed [1]. To do so, we physically confined cells to a height of 5 μm by using polydimethylsiloxane (PDMS) spacers [24] and compared spindle movements in flat cells in the presence (fibronectin [FN]-coated PDMS) (Figure 1F, top) or absence (polyethylene glycol [PEG]-coated PDMS) (Figure 1F, bottom) of adhesive cues. Under these conditions, the distribution of monopolar spindle velocities was unaffected by cell-substrate adhesions (Rap1^∗^ + STLC on FN: 0.85 ± 0.60 μm/min; PDMS + STLC on FN: 0.97 ± 0.40 μm/min; PDMS + STLC on PEG: 0.91 ± 0.27 μm/min; mean ± SD; p = 0.37; Kruskal Wallis non-parametric test) (Figures 1F and 1G). Similarly, monopolar spindles moved repeatedly back and forth along the long axis of cells plated on thin micropatterned lines of 10 μm width [24], apparently blind to the cell poles (Figures 1H and 1I)—where adhesion is strong and retraction fibers are concentrated. Monopolar spindles in these cells reached a dynamic steady state as they oscillated from one cell end to the other, reaching their maximum velocity as they crossed the cell center before pausing and turning (rather than flipping) to repeat the oscillation (Figures 1H and 1I). Taken together, these data suggest that spindle movements in this system are not influenced by local adhesive cues or retraction fibers.

In other systems, spindle orientation depends on Gαi, LGN, and NuMA [8], which recruit Dynein to the cortex, where it exerts forces on astral microtubules to move the spindle. Because all four proteins had a similar cortical distribution at the basal cortex of fixed flat monopolar cells (Figures S1J and S1K), we used GFP-LGN [12] as a proxy for the entire set of proteins in live-imaging experiments. Strikingly, the pattern of GFP-LGN accumulation in these experiments was both dynamic and closely correlated with monopolar spindle movement imaged by using α-tubulin-mCherry (Figures 2A and 2B; Video S1B). In addition, the pattern of GFP-LGN remained stable during periods in which the spindle remained in place (Figures S2D and S2I). In more detail, upon entry into mitosis, the distribution of LGN across the basal surface of flat cells appeared relatively homogeneous. Shortly thereafter, cortical LGN became depleted at the cell center. As LGN polarized, spindles tended to move off center toward regions of the cortex rich in LGN (Figure S2A). LGN was then lost from regions of the cortex that came to within ∼4 μm of the DNA that trailed behind the centrosome and microtubules (Figures 2A, 2B, 2F, top, 2G, left graph, S2B, and S2C), in line with the idea of a Ran-GTP-dependent inhibitory signal emanating from mitotic chromatin [12, 27]. At the same time, LGN was observed re-accumulating at regions of the cortex that were previously depleted of LGN (Figures 2A, 2B, S2D, and S2E). This led to a cycle of LGN loss from the cortex close to the chromatin and its re-accumulation at sites far from the moving spindle.

To determine whether these correlations between spindle movement and cortical LGN patterning reflect a causal relationship between the two systems, as expected based on previous work, we first treated cells with low doses of the microtubule depolymerizing drug nocodazole to assess whether monopolar spindle movements depend on astral microtubules, whose distribution we determined (Figures 2C, 2D, and S2G) [28, 29]. This proved to be the case: monopolar spindle movements were markedly slower in nocodazole-treated cells than in the control (Figure 2E, left) (p < 0.001; Mann-Whitney *U* test), leading to a pause in LGN dynamics (Figure S2I). Second, when we used RNAi to silence LGN expression, spindle movements were dramatically reduced as expected if LGN is required for force generation at the cortex (Figure 2E, right) (p < 0.001; Mann-Whitney *U* test). Third, to determine whether chromatin-based signals are responsible for the dynamic changes in the association of LGN with the cortex [12], we treated flat monopolar cells with importazole for short periods (Figure 2F) to interfere with chromatin-based Ran-GTP signaling [12, 27, 30]. Importazole reduced the clearance of LGN from the cortex close to chromatin, leading to a reduction in the LGN inhibition range (Figures 2F, 2G, and S2H) (4.5 μm control, 1.2 μm importazole-treated; p < 0.01; Mann-Whitney *U* test; see Methods S1), as expected based on previous work [27]. Additionally, in a rare flat, untreated cell in which a bipolar spindle broke into two, LGN was seen locally clearing from the basal membrane in the vicinity of both half-spindles (Figure S2F; Video S1C), implying that the effect is mediated by local short-range signaling. Together, these data support the idea that LGN and other cortical proteins controlling Dynein-mediated forces on astral microtubules, together with the Ran-GTP gradient centered on mitotic chromatin, constitute a dynamic feedback system that links the spindle and the cortex. This feedback prevents the system from reaching a static equilibrium state, giving rise to the striking instability of monopolar spindle positioning in flat cells.

Despite previous work suggesting a role for actin cortical mechanics in spindle orientation [2], we found no correlation between the organization of the actin cortex and the position or movement of the spindle (Figure S2J). In line with this, two perturbations that inhibited cortical myosin did not alter spindle movement (p > 0.05; Mann-Whitney *U* test) (Figures S2L and S2M). Nevertheless, when we disrupted the actin cortex by using high doses of latrunculin B, LGN (and associated membrane) was pulled toward the centrosome in a microtubule-dependent manner (Figure S2K). As previously suggested by work in *C. elegans* embryos and HeLa cells [31, 32], this implies that the actin cortex is not required for cortical motors to exert forces on the spindle. Instead, the cortex provides a stable platform that resists cortical deformation as the spindle moves.

**Figure 3:**
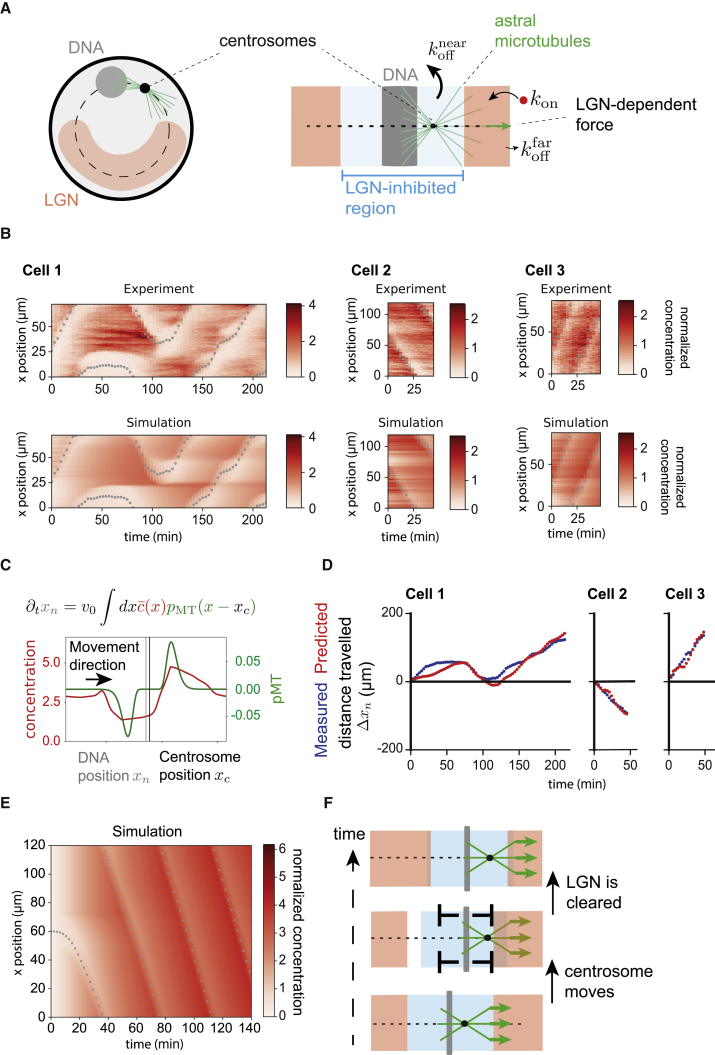
A mechanochemical model describing the LGN/DNA/microtubule interaction accounts for monopolar spindle motion (A) Schematic of 1D model simplification. Experimental data show DNA, centrosome positions, and LGN profiles projected on a circular line. These data are compared to a 1D model of monopolar spindle formation with periodic boundary conditions. In the model, LGN unbinding is faster near the DNA (blue region, rate $k_{\text{off}}^{\text{near}}$) than away from the DNA (red region, rate $k_{\text{off}}^{\text{far}}$). The centrosome/DNA/spindle structure is subjected to cortical forces acting on astral microtubules. These cortical forces increase with cortical LGN concentration. (B) Simulations of LGN inhibition by spindle motion recapitulate LGN experimental profiles. Top panels: experimental kymographs show the cortical LGN fluorescence intensity over space and time (red colors) and the DNA position over time (gray dots) for 3 representative cells. Bottom panels: corresponding kymographs of simulated cortical LGN concentration are shown, imposing the experimentally measured DNA position. In the simulation, the cortical LGN is modeled using simple binding/unbinding rates that depend on distance to the DNA. See Figure S3H for kymographs of 9 additional analyzed cells. Concentrations have been normalized to the spatiotemporal mean in kymographs. (C) DNA velocity is assumed to be set by the force exerted on the DNA-spindle-centrosome structure by cortical motors pulling on astral microtubules. Top: equation of motion for the DNA is shown, capturing the dependency of the force acting on the DNA-spindle-centrosome structure on the normalized LGN concentration $\bar{c}$ and the signed distribution of microtubule ends, $p_{\text{MT}}$. $x_{n}$ and $x_{c}$ are the positions of the DNA center and of the centrosome. Bottom: example plot of normalized LGN concentration (red) and the distribution of microtubules (green) in the model are shown. (D) Comparison to experimental data of DNA motion predicted in (C). Graphs show the distance traveled by the DNA $\text{Δ}x_{n}$ as a function of time, experimentally measured (blue) and predicted from the equation given in (C) (red) for 3 representative cells. The value of the proportionality coefficient $v_{0}$ is chosen independently for each cell. See Figure S3I for graphs of 9 additional analyzed cells. (E) LGN concentration and spindle motion in full mechanochemical feedback model (colors are as in B). The system settles in a steady state characterized by constant DNA velocity and a traveling wave of LGN concentration. Parameters are given in Methods S1. (F) Schematic of mechanism of steady-state motion. The depletion of LGN around DNA results in an asymmetric distribution of LGN around the centrosome and a net force acting on the spindle. As a result of this force, the DNA-centrosome-spindle structure moves. Further LGN depletion around the DNA results in a traveling wave of LGN concentration. See also Figure S3.

To better understand how such dynamical feedback between the cortex and the spindle is likely to work, we developed a computational model of monopolar spindle movement in flat cells. This model includes (1) DNA-dependent inhibition of cortical LGN and (2) cortical dynein motors that pull on astral microtubules to exert forces on the spindle (Figures 3A and S3A). In the model, cortical LGN diffuses on the cell surface and undergoes exchange with cytoplasmic LGN with on and off rates denoted by${\text{k}}_{\mathrm{on}/\mathrm{off}}$, under the assumption that the cytoplasmic pool of LGN acts as a large reservoir. Cortical LGN is affected by spindle movement because dissociation of LGN from the cortex ($k_{\mathrm{off}}$ rate) occurs preferentially near the DNA, as observed in experiments (Figures 2A, 2B, 2F, and 2G; Videos S1B and S1C). To test whether this simple model can account for the observed dynamics of LGN, we quantified the position of the DNA, centrosomes, and LGN profiles in flat cells along the path of monopolar spindle movement (Figure 2A, top, last frame), which we visualized in kymographs as a 1D, periodic motion (Figures 3B and S3H). Taking the motion of DNA as an input, we then obtained theoretical cortical LGN profiles (Figures 3B and S3H) by using a small value for the diffusion constant ($D=0.01\;\text{μ}{\text{m}}^{2}/\text{min}$), consistent with the experimentally observed stability of cortical accumulations of LGN far from the spindle (Figures S2D and S2E). With unbinding occurring near the DNA with a characteristic timescale of ∼10 min and far from the DNA with a timescale of ∼90 min, kymographs generated from simulations reproduced key features of the experimental data (Figures 3B and S3H; Methods S1). We then assumed monopolar spindle movement to be driven by forces acting on the end of astral microtubules, taken as proportional to the sampled cortical LGN concentration at microtubule plus ends. Using our experimental measure of the astral microtubule length distribution (Figures 2C, 2D, 3C, S2G, and S3B), this simple model was also able to account for the experimentally observed motion of the DNA in the 12 cells we studied in detail (Figures 3D, S3I, and S3J), by adjusting a free parameter $v_{0}=6.7\pm \;6.7\text{μm}/\text{min}.$ This characteristic velocity depends on the force exerted by dynein motors at a reference LGN concentration, the number of microtubules, and the friction coefficient acting on the spindle (Methods S1).

Using the parameters defined above to generate a 1D dynamic model of a monopolar spindle, we found that, starting with a uniform concentration of LGN (Figures 3E and S3C), the spindle eventually moves with a constant velocity, following a traveling wave of LGN (Figures 3E, 3F, S3C, and S3D). This closely resembles monopolar spindle movement in real cells (Figures 3B, 3D, and S3H–S3J) and suggests that there is no stable rest state for a monopolar spindle configuration in which the centrosome and DNA are physically separate (Figure S3E; Methods S1). Interestingly, the model predicts that, even if the centrosome and DNA occupy the same position (e.g., equivalent to the centrosome lying on top of the DNA in 3D), the system can still undergo spontaneous symmetry breaking, leading to monopolar spindle movement (Figures S3C and S3F; Methods S1), as observed in flat monopolar cells entering mitosis (Figure S2A). Simulations also replicated the back-and-forth oscillations seen in cells plated on thin micropatterned lines (Figures S3K–S3N, 1H, and 1I), although this 1D model cannot capture spindle turns.

By modifying the model to study the movement of bipolar spindles in flat cells, we found that, in addition to a non-moving solution arising from the inherent symmetry of the bipolar configuration, the coupled spindle and LGN dynamics can also give rise to bipolar spindle movement (Figure S3G), depending on parameters (Figures S3U–S3X). This fits with our previous work, in which we showed that spindles move off center in cells that are prevented from rounding [24, 33], and with bipolar spindle behavior in Rap1^∗^ cells confined to 1D line micropatterns (Figures S3O–S3T and S3Y).

**Figure 4:**
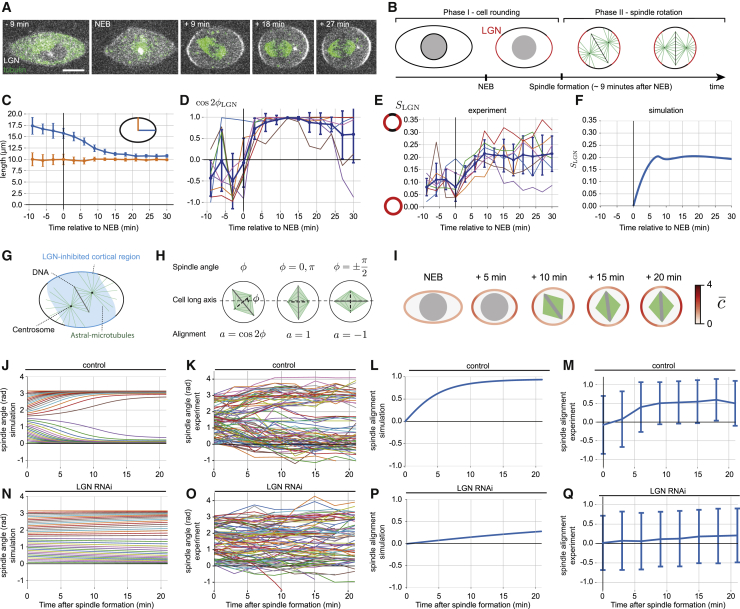
Mechanochemical model is sufficient to explain how bipolar spindles align with the interphase long cell axis as cells round (A) Experimental images showing bipolar spindle formation and reorientation upon entry into mitosis. Confocal images of a representative HeLa cell expressing GFP-LGN on a FN-coated unpatterned substrate show cell shape changes around NEB and bipolar spindle formation and reorientation after NEB. LGN is inhibited along the short axis of the cell and can freely accumulate at the two crescents corresponding to the interphase long axis of the rounded mitotic cell. The scale bar indicates 10 μm. (B) Schematic of the main phases of bipolar spindle orientation. In phase 1, the cell undergoes mitotic rounding and LGN accumulates in the cortex after NEB as cells assemble a bipolar spindle. In phase 2, the bipolar spindle reorients in the rounded cell. (C) Quantification of cell shape changes during mitotic rounding for mitotic HeLa cells (n = 7), for which (A) is a representative example. Blue, long cell axis half-length; orange, short cell axis half-length. Error bars, standard deviation. (D) Alignment of the nematic angle calculated from LGN fluorescence intensity profiles with the long cell axis for mitotic HeLa cells (n = 7), for which (A) is a representative example. Thick blue line, dots, and error bars indicate the average and standard deviation of alignment for different quantified cells. Other thin lines show the alignment for individual cells. A strong alignment with the long cell axis (average alignment close to 1) is reached ~10 min after NEB. (E) Magnitude of nematic order parameter $S_{\text{LGN}}$ for LGN concentration as a function of time (see Methods S1 for a definition) for mitotic HeLa cells (n = 7), for which (A) is a representative example. Thick blue line, dots and error bars indicate the average and standard deviation of alignment for different quantified cells. Other thin lines correspond to $S_{\text{LGN}}$ for individual cells. The nematic order parameter increases between 0 and 10 min after NEB and stabilizes after 10 min. (F) Magnitude of nematic order parameter $S_{\text{LGN}}$ as a function of time, for the simulation shown in (I). The initial rise after NEB of LGN ordering in the cell contour is comparable to that measured in experiments in (E). (G) Schematic of mechanochemical model for bipolar spindle orientation. In the model, loss of LGN from the cortex is induced within a range of 4 μm away from the DNA. LGN influences the cortical forces acting on the astral microtubules from the cortex, resulting in a torque acting on the spindle and driving its reorientation after spindle formation. At the same time, the cell undergoes mitotic cell rounding. (H) An alignment parameter a=cos2φ can be defined for φ the spindle angle in relation to the interphase long cell axis, quantifying how good (a→1) or bad (a→−1) spindle alignment is with respect to the long axis. (I) Images depict outputs from the spindle orientation simulation in a cell undergoing mitotic rounding, at different times after NEB. The initial angle of the spindle in relation to the long axis is φ=π/4. The red color on the cell contour corresponds to the concentration of LGN: DNA is represented by a gray circle up to 9 min after NEB and by a gray line after 9 min after NEB, to represent the metaphase plate. For simplicity, astral microtubules are not depicted, and spindle microtubules are shown as two triangles to visualize the spindle orientation. In the simulation, LGN accumulation due to cell rounding and feedback from DNA inhibition leads to spindle rotation along the long cell axis. (J, L, N, and P) Predicted spindle dynamics as cell round, with normal or reduced levels of LGN, as a function of time. Spindle dynamics were obtained from simulations as shown in (I). (J and N) Simulated trajectories of spindle angles in relation to long axis as a function of time, in control conditions (J) and for reduced LGN concentrations (N). (L and P) Average alignment of the spindle angle with respect to the long axis, as a function of time, obtained from simulations in (J) and (N), respectively. The alignment is defined in (H). Simulation trajectories are obtained by choosing a set of uniformly spaced initial spindle angles. For each simulation, spindle rotation is determined by the torque exerted by LGN-dependent forces on astral microtubules, in a cell which changes its shape. See Methods S1 for details. (K, M, O, and Q) Plots of spindle orientation trajectories in control (n = 94) and LGN RNAi (n = 96) cells on micropatterned lines fit with predicted behavior. Experimental trajectories of spindle angles in relation to the long cell axis in control conditions (K) and LGN-RNAi (O) (1 of 96 trajectories is out of range) are shown. (M and Q) Average alignment of the spindle angle with respect to the long axis as a function of time, obtained from experiments in (K) and (O), respectively. See also Figure S4 and Videos S1D–S1F.

Finally, we wondered whether the mechanochemical model we identified in flat cells could shed light on the functional consequences of dynamic crosstalk between the spindle and cortex in the context of a normal mitosis, where cells assemble and orient a bipolar spindle as they round. To do so, we generated a 2D model on the basis of the parameters defined above (Figures S4A and S4B; Methods S1), in which we included the dynamics of cell rounding (Figures 4A and S4C; Video S1D) [34] and bipolar spindle assembly (completed ∼9 min after nuclear envelope breakdown [NEB] [24]) (Figures S4D and S4E). For simplicity, using these values, we modeled mitosis as two distinct phases (Figure 4B). In phase I, as cells round, DNA-mediated inhibition patterns cortical LGN (Figures 4B and S4C). In phase II, from 9 min after NEB onward, the bipolar spindle interacts with cortical motors through astral-microtubule-mediated forces, in an LGN-dependent manner (Figures 4B and 4G). During this second phase, we assume that the spindle rotates according to the torque arising from cortical forces and acting on astral microtubules, in line with previous models of spindle orientation [1, 20, 21, 22]. We then compared simulation results to experiments in which we observed cells expressing GFP-LGN as they rounded (Figure 4A). In both experiments and in the model, LGN crescents formed at the poles along the long cell axis in the first ∼9 min after NEB (experiment: Figures 4A, 4D, 4E, and S4I–S4K; model: Figures 4F, 4I, S4J, and S4L). In the model, this can be understood as a simple consequence of LGN accumulation due to cell rounding and DNA-mediated inhibition of LGN along the short cell axis, as previously observed [12, 27]. Moreover, this polarized distribution of LGN was retained for an extended period of time after spindle reorientation in both simulations and experiments (Figures 4A, 4D–4F, 4I, and S4I). Then, in phase II, astral-microtubule-mediated forces act on the bipolar spindle to change its alignment to the interphase long cell axis (Figures 4G and 4H). As it does so, the polarized distribution of LGN (established in phase I) leads to an imbalance in the cortical forces acting on astral microtubules, leading to spindle rotation, even though cells are round, i.e., in the absence of a geometrical cue (Figure 4I). In fact, this simple 2D model of cell-intrinsic signaling recapitulates the reorientation of mitotic spindles toward the interphase long cell axis (Figure 4I) in rounded mitotic cells, across a wide range of initial angles (Figures 4J, 4K, and S4G), yielding spindle reorientation dynamics that are similar to those observed in HeLa cells rounding up and dividing on FN-coated micropatterned lines (Figures 4L and 4M), where there is a strong interphase shape signal (Figure S4C). Further, as expected, spindle alignment was compromised in experiments where LGN was depleted through RNAi and in equivalent simulations where LGN levels were strongly reduced (Figures 4N–4Q, S4F, and S4H; Videos S1E and S1F).

Together, these data indicate that the dynamic DNA/LGN mechanochemical system, which we defined on the basis of monopolar spindle behavior in flat cells (Figures 1, 2, and 3), is sufficient to enable cells undergoing mitotic rounding to translate interphase cell shape into a cortical pattern of LGN, which persists after cells become completely rounded (Figure 4). Although spindle movement in this system does not depend on cell-substrate mitotic adhesion sites (Figures 1F–1I) and is unaffected by perturbations that affect cortical mechanics (Figures S2L and S2M), local differences in cortical stiffness will likely impact spindle movements and alignment [2, 32]. Furthermore, these observations do not mean that external cues cannot influence spindle orientation in this context. When a strong external cue perpendicular to the interphase cell axis was introduced into the model, the spindle rotated toward the external cue and maintained a stable orientation perpendicular to the interphase cell axis (Figure S4M). This shows that, although this intrinsic patterning system is sufficient to position the spindle, it can be easily over-ridden. In these more complex scenarios, the influence of the intrinsic patterning based on spindle-cortical crosstalk functions will depend on other factors, such as the strength of extrinsic cues, gradients in cortical stiffness, the relative size of the cell and spindle, and the persistence of cell elongation (Figures S4M–S4Q), in ways that will be interesting to explore in the future. It was by studying the simplest case, though, that we were able to uncover rules of dynamic spindle-cortical crosstalk, which, as we show, are sufficient to explain how spindles orient in relation to the interphase long cell axis as cells round in the absence of any extrinsic signals.

## STAR★Methods

### Key Resources Table

**Table:** 

REAGENT or RESOURCE	SOURCE	IDENTIFIER
**Antibodies**		

Mouse monoclonal anti-α-tubulin	Sigma-Aldrich	Cat#T9026; RRID: AB_477593
Mouse monoclonal anti-α-tubulin−FITC	Sigma-Aldrich	Cat#F2168; RRID: AB_476967
Chick anti-GFP	abcam	Cat#ab13970; RRID: AB_300798
Rabbit anti-NuMA	abcam	Cat#ab84680; RRID: AB_2154610
Mouse monoclonal G_*αi*1_	Santa Cruz Biotechnology	Cat#sc-13533; RRID: AB_2111358
Goat anti-mouse secondary antibody, Alexa Fluor 488	ThermoFisher	Cat#A-11001; RRID: AB_2534069
Goat anti-chick secondary antibody, Alexa Fluor 488	ThermoFisher	Cat#A-11039; RRID: AB_2534096
Goat anti-mouse secondary antibody, Alexa Fluor 546	ThermoFisher	Cat#A-11030; RRID: AB_2534089
Goat anti-rabbit secondary antibody, Alexa Fluor 546	ThermoFisher	Cat#A-11035; RRID: AB_2534093
Goat anti-mouse secondary antibody, Alexa Fluor 647	ThermoFisher	Cat#A-21241; RRID: AB_2535810
Goat anti-mouse secondary antibody, Alexa Fluor 405	ThermoFisher	Cat#A-31553; RRID: AB_221604

**Chemicals, Peptides, and Recombinant Proteins**		

DMEM GlutaMAX	ThermoFisher	Cat#10566016
Fetal Bovine Serum (FBS)	ThermoFisher	Cat#16000044
Penicillin-Streptomycin	ThermoFisher	Cat#15070063
G418	Sigma-Aldrich	Cat#345810
Puromycin	Sigma-Aldrich	Cat#P8833
FuGENE HD	Promega	Cat#E2311
Lipofectamine LTX Reagent with PLUS Reagent	ThermoFisher	Cat#15338030
Fibronectin	Sigma-Aldrich	Cat#F1141
Lipofectamine 2000	ThermoFisher	Cat#11668030
Latrunculin B	Sigma-Aldrich	Cat#428020
Nocodazole	Sigma-Aldrich	Cat#487929
Y-27632	Sigma-Aldrich	Cat#Y0503
Importazole	Sigma-Aldrich	Cat#SML0341
S-trityl-L-cysteine (STLC)	Sigma-Aldrich	Cat#164739
PLL-g-PEG	SuSOS	Cat#pll20-g3-5-peg2
Fibrinogen-Alexa Fluor 647	ThermoFisher	Cat#F35200
Trypsin-EDTA	ThermoFisher	Cat#R001100
Formaldehyde	TAAB	Cat#F017
Triton X-100	Sigma-Aldrich	Cat#T8787
FluorSave	Sigma-Aldrich	Cat#345789
Phalloidin-TRITC	Sigma-Aldrich	Cat#P1951
DAPI	Sigma-Aldrich	Cat#D9542

**Experimental Models: Cell Lines**		

Human: HeLa Kyoto cells	[35]	N/A
Human: HeLa H2B-mRFP/tubulin-GFP	[35]	N/A
Human: HeLa GFP-LGN	[12]	N/A
Human: HeLa DHC-GFP	[12]	N/A

**Oligonucleotides**		

siRNAs against LGN (GAACUAACAGCACGACUUA)	[12]	N/A

**Recombinant DNA**		

pRK5-Rap1[Q63E] (Rap1^∗^)	[25]	N/A
pmCherry-α-tubulin-IRES-puro2	[35]	N/A
H2B-mCherry	addgene	20972
lifeact-iRFP	[36]	N/A

**Software and Algorithms**		

Volocity	Quorum Technologies	https://www.quorumtechnologies.com/volocity
Fiji	[37]	https://fiji.sc/
Custom Python 3 analysis scripts	This paper	https://github.com/andimi/spindle-orientation
Custom Python 3 modeling scripts	This paper	https://github.com/salbreux/Spindle

**Other**		

12-well glass-bottom plates	MatTek	Cat#P12G-1.5-14-F
35 mm glass-bottom dishes	MatTek	Cat#P35G-1.5-14-C
4-well Lab-Tek II Chamber Slide	Sigma-Aldrich	C6807
Axiovert 200M	Zeiss	N/A
Observer Z1	Zeiss	N/A
Eclipse Ti	Nikon	N/A
Retiga EXi camera	QImaging	N/A
UltraView VOX	Perkin Elmer	N/A
ImagEM camera	Hamamatsu	N/A
TCS SPE laser scanning confocal microscope	Leica	N/A

### Resource Availability

#### Lead Contact

Further information and requests for resources and reagents should be directed to and will be fulfilled by the Lead Contact, Buzz Baum (b.baum@ucl.ac.uk).

#### Materials Availability

There are no restrictions on any data or materials presented in this paper.

#### Data and Code Availability

Data and Code are available at https://github.com/andimi/spindle-orientation and https://github.com/salbreux/Spindle

### Experimental Model and Subject Details

Unlabeled HeLa Kyoto cells and HeLa stable cell lines expressing H2B-mRFP/tubulin-GFP [35], GFP-LGN and DHC-GFP [12], were cultured under standard conditions. They were maintained in Dulbecco’s Modified Eagles Medium (DMEM GlutaMAX; ThermoFisher) supplemented with 10% FBS (ThermoFisher) and 50 U/ml penicillin and 50 μg/ml streptomycin (ThermoFisher) at 37 ◦C under 5% CO2. Where appropriate, medium was supplemented with selective antibiotics, 0.64 mg/ml G418 (Sigma-Aldrich) and 0.5 μg/ml puromycin (Sigma-Aldrich).

### Method Details

#### DNA and siRNA transfection

HeLa cells were transfected with pRK5-Rap1[Q63E] (Rap1^∗^ throughout this text) [25], pmCherry-α-tubulin-IRES-puro2 (tubulin-mCherry) [35], H2B-mCherry (addgene plasmid #20972), or lifeact-iRFP [36] using FuGENE HD (Promega), or Lipofectamine LTX with Plus reagent (ThermoFisher), according to the manufacturers’ instructions. 20,000 HeLa cells were plated in 12-well plates, in 35 mm glass-bottom dishes (MatTek), in 4-well Lab-Tek (Sigma-Aldrich), or on 10 mm coverslips coated with 10 *μ*g/ml fibronectin (Sigma-Aldrich). The following day, the culture medium was changed for DMEM supplemented with 10% FBS without antibiotics. For transfections with Fugene HD or Lipofectamine LTX with Plus reagent, cells were processed for microscopy 24 hours later to allow expression of the plasmids. For transfection in larger culture dishes, the procedure was scaled appropriately. Control transfection reactions were performed in the absence of plasmid DNA. Cells transfected with Rap1^∗^ were identified by their failure to round up in mitosis. HeLa cells were transfected with siRNAs against LGN (GAACUAACAGCACGACUUA) as in [12], using Lipofectamine 2000 (Invitrogen) as previously described [24]. Cells were processed for microscopy after 48 hours. Where RNAi was performed in conjunction with Rap1^∗^ expression, cells were first transfected with siRNAs and sequentially transfected with the Rap1^∗^ plasmid for the final 24 hours.

#### Drug treatments

Cells were treated with 5 *μ*M latrunculin B (Sigma-Aldrich), 10 ng/ml (low doses) or 200 ng/ml (high doses) nocodazole (Sigma-Aldrich), 10 *μ*M Y27632 (Sigma-Aldrich), 40 *μ*M importazole (SigmaAldrich), 5 *μ*M S-trityl-L-cysteine (STLC; Sigma-Aldrich), and, where indicated, control treatments were performed with an equivalent volume of the solvent DMSO.

#### Micropatterning and Cell Confinement

Micropatterned islands of fibronectin were fabricated with deep UV light [38] on 25 mm coverslips. Glass-bottom dishes were coated with non-adhesive polyethylene glycol, PLL-g-PEG (SuSOS, Switzerland) for 1 h, before deep UV illumination through a photomask. Then, a 25 μg/ml fibronectin solution (Sigma-Aldrich) together with Alexa Fluor 405 or Fibrinogen-Alexa Fluor 647 (ThermoFisher) were incubated for 1 h at room temperature. HeLa cells expressing Rap1^∗^ were trypsinised (using Trypsin-EDTA; ThermoFisher), resuspended in medium at a density of 60,000 cells/ml, seeded onto micropatterned glass-bottomed dishes, and incubated at 37 ^◦^C under 5% CO_2_ for 1 hr. After 1 hr, the cells were washed in fresh medium and incubated for 4 h to allow spreading on patterned fibronectin before they were processed for microscopy.

For confinement assays, cells were seeded as above on glass-bottom 6-well plates, either on fibronectin-coated, or on PLL-g-PEG-coated glass-bottom dishes. The next day, cells were confined in a defined space (5 μm) as previously described [24, 39]. Briefly, micropillar spacers of the desired height were molded onto a thin layer of PDMS coating 10-mm-diameter glass coverslips. Pillars were coated with either (adhesive) fibronectin, or (anti-adhesive) PLL-g-PEG. Then, these pillars were positioned onto the cells, confining them with sub-micron homogeneity.

#### Live-cell microscopy

For live-cell imaging, cells were seeded on glass-bottomed dishes (MatTek) coated with 10 mg/ml fibronectin (Sigma-Aldrich), or on micropatterned fibronectin. For mitotic timing experiments, cells were imaged every 2, 3, or 5 min. For live cell microscopy, cells were imaged with a Zeiss Axiovert 200M or Observer Z1 or Nikon Eclipse Ti microscope with a 20X objective (numerical aperture, NA 0.5) or 10X objective (NA 0.3) or 40X oil objective (NA 1.3) equipped with temperature and CO_2_ controlling environmental chambers and images acquired using a Retiga EXi camera (Qimaging) and Volocity software (Perkin Elmer). For live cell confocal microscopy, cells were imaged using an UltraView VOX (Perkin Elmer) spinning disc confocal microscope with a 40X (NA 0.75) air objective or 60X (NA 1.4) oil objective equipped with temperature and CO_2_ controlling environmental chambers, and images were acquired using a Hamamatsu ImagEM camera and Volocity software (Perkin Elmer).

#### Immunofluorescence and antibodies

For immunofluorescence, cells on fibronectin-coated glass coverslips were fixed with 4% formaldehyde (TAAB), permeabilized with 0.5% Triton X-100 (Sigma-Aldrich) in PBS for 5 min, and blocked with 5% bovine serum albumin (Sigma-Aldrich) in PBS for 30 min. The cells were sequentially incubated with primary and fluorescently labeled secondary antibodies for 1 h at room temperature and then washed in PBS, 0.1% Triton X-100. The cells were mounted in FluorSave (Sigma-Aldrich) and images were acquired using a Leica TCS SPE laser scanning confocal microscope system.

For immunofluorescence, primary antibodies were used at the following dilutions: α-tubulin 1:200 (mouse monoclonal DM1A; Sigma-Aldrich); FITC-conjugated *α-*tubulin 1:500 (mouse monoclonal DM1A; Sigma-Aldrich); GFP (chick; abcam); NuMA (rabbit; abcam); G_*αi*1_ (mouse monoclonal; Santa Cruz Biotechnology). Secondary anti-mouse, anti-rabbit, or anti-chick IgG antibodies (ThermoFisher) tagged with Alexa Fluor 488, 546 or 647 were used at 1:500. Actin was visualized with TRITC-conjugated phalloidin at 1 *μ*g/ml (Sigma-Aldrich), and DNA with DAPI at 1 *μ*g/ml (Sigma-Aldrich).

### Quantification and Statistical Analysis

#### Quantification

Images were processed using Fiji/ImageJ [37], and, where necessary, contrast/brightness was changed uniformly (linearly) across the field. To measure cell length and height, the x, y, and z scales of microscopes were calibrated using 19.28 ± 0.3 mm beads coated with fluorescent PLL-g- PEG by soaking for 30 min in a 0.5 mg/ml PLL-g-PEG solution in 10 mM HEPES pH 7.4 after plasma activation. Measurements and analyses (e.g., cell structure dimensions, distances between elements within the cell, etc) were performed either by using Fiji/ImageJ built-in functions, or custom Python scripts (https://github.com/andimi/spindle-orientation). Centrosome positions were manually tracked, and DNA outlines were manually or semi-automatically traced, and then fitted to an ellipse to extract geometrical parameters. The LGN intensity used in the analysis was normalized by subtracting the background signal outside the cell, and by dividing the resulting intensity by the average intensity calculated in the cell, both in space and time. For the graphical representation of LGN kymographs shown in Figure S3, LGN intensity values were normalized by the average intensity in space, frame-by-frame.

#### Statistical analysis and visualization

Two sample t test and Mann-Whitney U test were implemented using the scipy library in Python, to compare the mean or median of data from controls and experiments. When samples were assumed to be drawn from a normal distribution, the t test was used, and the Mann-Whitney U test otherwise. The statistical details of experiments can be found in the Figure legends and in the Results section. Unless otherwise stated, n represents the number of cells. Significance was defined as p values < 0.05, and the following notation was used in the Figures: “^∗^” for 0.01 < p values < 0.05; “^∗∗^” for 0.001 < p values < 0.01; and “^∗∗∗^” for p values < 0.001. The plotnine module (implementing the ggplot R library in python), or the matplotlib/seaborn modules were used for data visualisation. Box and whisker plots show median, upper (75th percentile), and lower (25th percentile) quartiles as the box; whiskers represent the range of the data above the 75th percentile and below the 25th percentile and extend up to 1.5 times the interquartile distance (the difference between the 75th and 25th percentiles). All data points were included in the statistical analysis and in the plots.

## Supplemental Information

Document S1. Figures S1–S4

Methods S1. Dynamic equations for monopolar spindle motion in flat mitotic cells and for bipolar spindle orientation in cells undergoing mitotic rounding, related to STAR Methods

Document S2. Article plus Supplemental Information
