# Supplementary material for: Mechanochemical Crosstalk Produces Cell-Intrinsic Patterning of the Cortex to Orient the Mitotic Spindle
Source: Curr Biol. 2020 Sep 21;30(18):3687–3696.e4. doi: 10.1016/j.cub.2020.06.098 (PMC7521479; doi:10.1016/j.cub.2020.06.098)
Supplement: Document S1. Figures S1–S4 [file mmc1.pdf]

Current Biology, Volume *30*

Supplemental Information

Mechanochemical Crosstalk

Produces Cell-Intrinsic Patterning

# of the Cortex to Orient the Mitotic Spindle

Andrea Dimitracopoulos, Pragya Srivastava, Agathe Chaigne, Zaw Win, Roie Shlomovitz, Oscar M. Lancaster, Maël Le Berre, Matthieu Piel, Kristian Franze, Guillaume Salbreux, and Buzz Baum

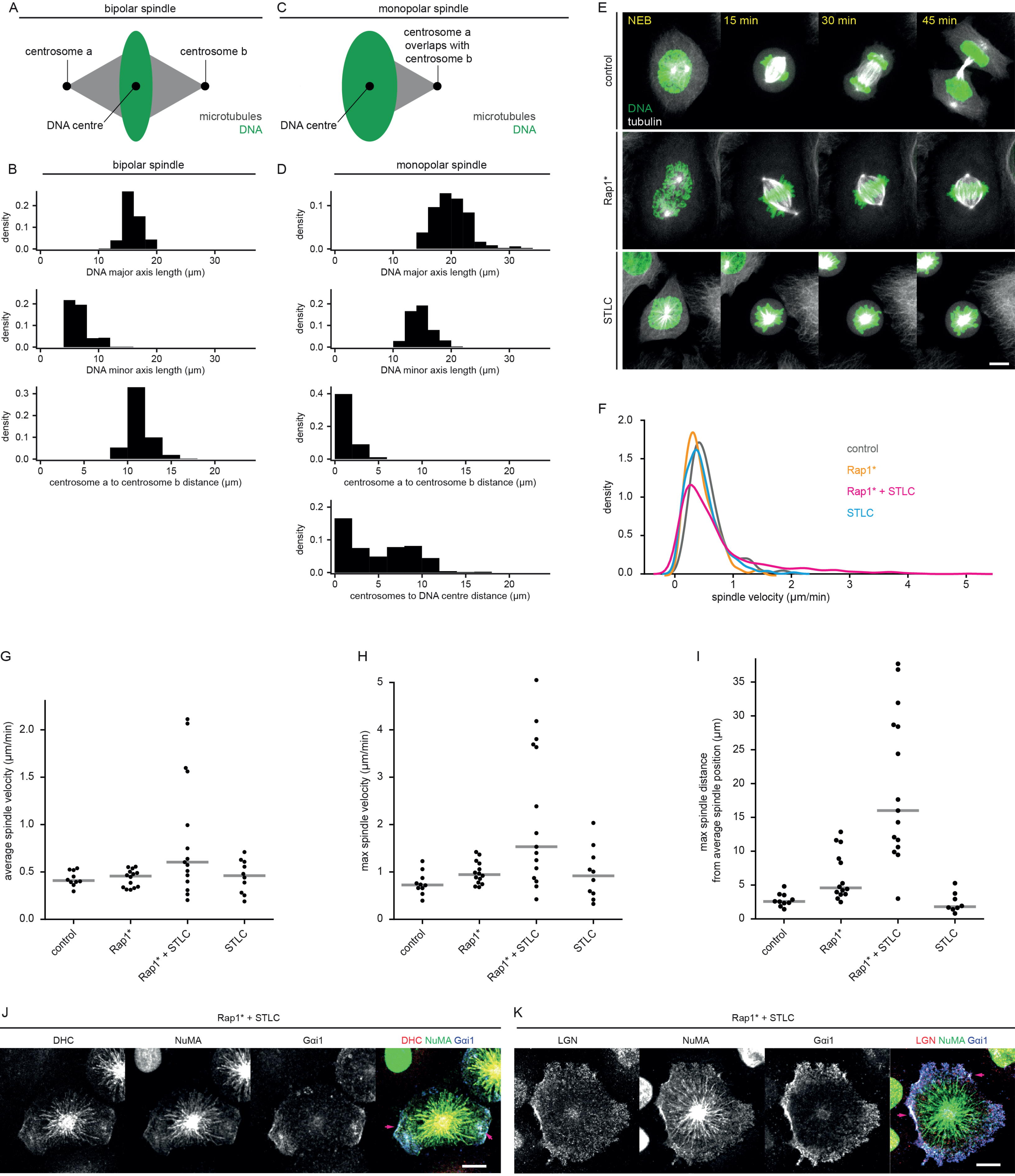
A
bipolar spindle
centrosome a
centrosome b
DNA centre
microtubules
DNA
C
monopolar spindle
centrosome a overlaps with centrosome b
DNA centre
microtubules
DNA
B
bipolar spindle
density
DNA major axis length (µm)
DNA minor axis length (µm)
centrosome a to centrosome b distance (µm)
D
monopolar spindle
density
DNA major axis length (µm)
DNA minor axis length (µm)
centrosome a to centrosome b distance (µm)
centrosomes to DNA centre distance (µm)
E
NEB
15 min
30 min
45 min
control
DNA
tubulin
Rap1*
STLC
F
density
spindle velocity (µm/min)
control
Rap1*
Rap1* + STLC
STLC
G
average spindle velocity (µm/min)
H
max spindle velocity (µm/min)
I
max spindle distance from average spindle position (µm)
control
Rap1*
Rap1* + STLC
STLC
J
Rap1* + STLC
DHC
NuMA
Gαi1
DHC NuMA Gαi1
K
Rap1* + STLC
LGN
NuMA
Gαi1
LGN NuMA Gαi1

Figure S1 – The geometrical parameters of bipolar and monopolar spindles, and spindle movements in cells treated with different combinations of Rap1* and STLC. Related to Figure 1.

A – Schematic representing a fully formed bipolar spindle (15 min or longer after NEB) under control condition.

B – Histograms of the geometrical parameters (DNA major/minor axis length, and centrosome-to-centrosome distance) that characterise a fully formed bipolar spindle (15 min or longer after NEB) in cells on FN-coated unpatterned substrates (as in **A**).

C – Schematic representing a monopolar spindle (in Rap1* +STLC treated cells, where centrosomes fail to separate).

D – Histograms of the geometrical parameters (DNA major/minor axis length, centrosome-to-centrosome distance, and centrosome-to-DNA distance) that characterise a monopolar spindle in cells on FN-coated unpatterned substrates (as in **C**).

E –Time-lapse confocal images of representative HeLa cells on FN-coated unpatterned substrates in 3 conditions as they enter mitosis: (top) control, (middle) Rap1*, and (bottom) STLC. Cells are expressing tubulin-GFP and H2B-mCherry. The scalebar indicates 10 µm.

F – Density plots of spindle velocities in cells on FN-coated unpatterned substrates in 4 different conditions: control, Rap1*, Rap1* + STLC, and STLC.

G – Plot showing average spindle velocities for cells used in **F**.

H – Plot showing maximum spindle velocities for cells used in **F**.

I – Plot showing the maximum distance between the spindle at any time during mitosis from its average position in the 4 conditions (as in **F**).

J, K – Immuno-fluorescence confocal images of HeLa cells in mitosis (Rap1* + STLC) on FN-coated unpatterned substrates. **J**. Dynein (DHC) and NuMA are found localised to spindle microtubules and the cell cortex far from the spindle - where Gαi1 also localises. **K**. NuMA is found localised to spindle microtubules and the cell cortex - where LGN and Gαi1 also localise.

Regions where the three proteins overlap are shown in white in the overlay and highlighted by magenta arrows.

All scalebars indicates 10 µm. In **G**, **H**, and **I** grey bars indicate median values.

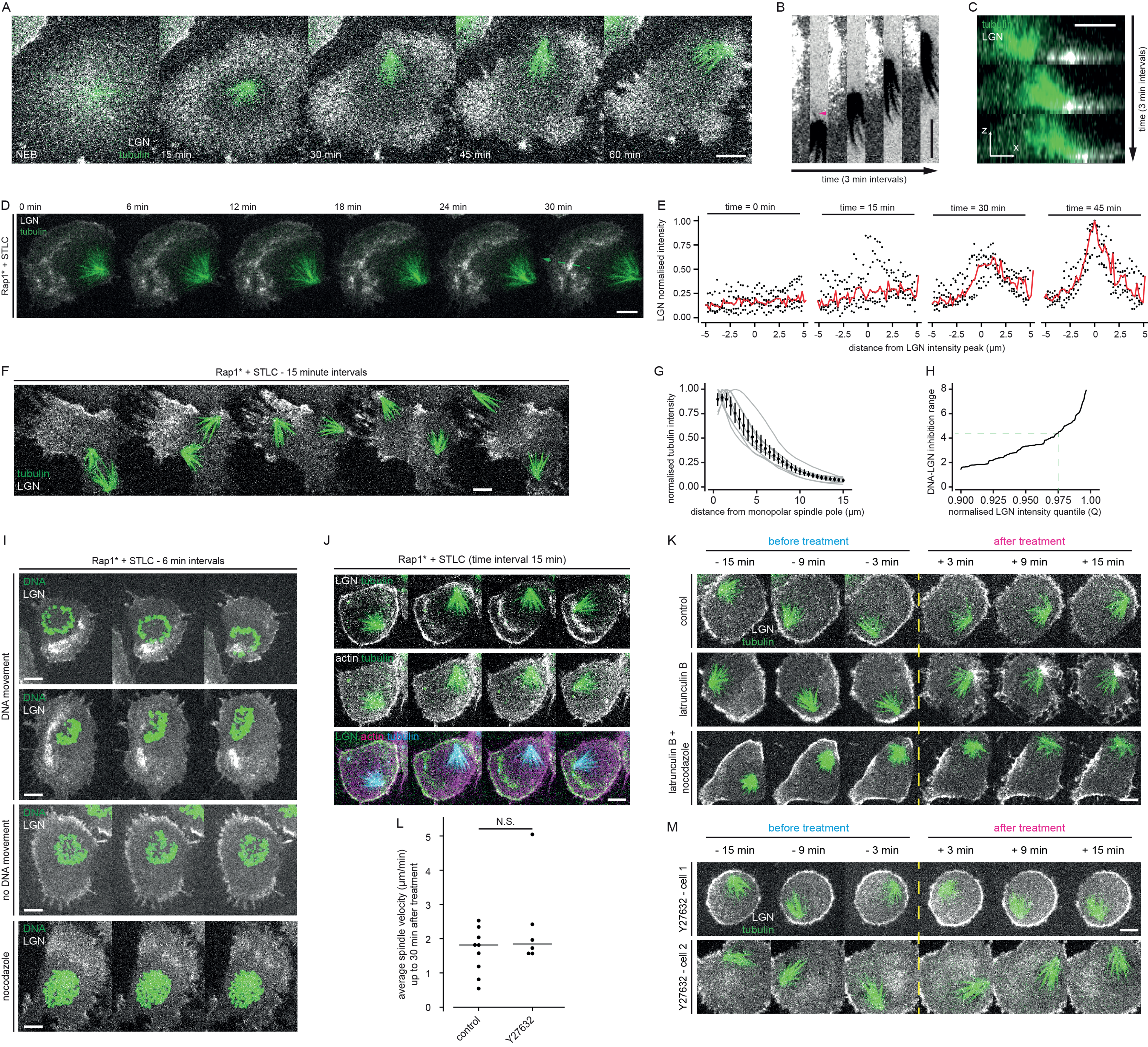

A
LGN
tubulin
NEB
15 min
30 min
45 min
60 min
B
time (3 min intervals)
C
tubulin
LGN
z
x
time (3 min intervals)
D
0 min
6 min
12 min
18 min
24 min
30 min
LGN
tubulin
Rap1* + STLC
E
time = 0 min
time = 15 min
time = 30 min
time = 45 min
LGN normalised intensity
1.00
0.75
0.50
0.25
0.00
-5
-2.5
0
2.5
5
distance from LGN intensity peak (µm)
F
Rap1* + STLC - 15 minute intervals
tubulin
LGN
G
normalised tubulin intensity
1.00
0.75
0.50
0.25
0.00
0
5
10
15
distance from monopolar spindle pole (µm)
H
DNA-LGN inhibition range
8
6
4
2
0
0.900
0.925
0.950
0.975
1.00
normalised LGN intensity quantile (Q)
I
Rap1* + STLC - 6 min intervals
DNA
LGN
DNA movement
no DNA movement
nocodazole
J
Rap1* + STLC (time interval 15 min)
LGN tubulin
actin tubulin
LGN actin tubulin
K
before treatment
after treatment
- 15 min
- 9 min
- 3 min
+ 3 min
+ 9 min
+ 15 min
control
LGN
tubulin
latrunculin B
latrunculin B + nocodazole
L
N.S.
average spindle velocity (µm/min) up to 30 min after treatment
5
4
3
2
1
0
control
Y27632
M
before treatment
after treatment
- 15 min
- 9 min
- 3 min
+ 3 min
+ 9 min
+ 15 min
Y27632 - cell 1
LGN
tubulin
Y27632 - cell 2

Figure S2 – LGN dynamics in flat mitotic cells with a monopolar spindle, and the role of the actomyosin cortex in monopolar spindle movement and LGN cortical stability. Related to Figure 2.

A – After NEB, LGN is inhibited near the spindle and accumulates far from the spindle. Time-lapse images of a mitotic HeLa cell (Rap1* + STLC) on a FN-coated unpatterned substrate (confocal imaging), showing dynamics of LGN accumulation and inhibition as a monopolar spindle moves across the cell.

B – Detail of a monopolar spindle in a cell on a FN-coated unpatterned substrate following high levels of LGN (left, high levels in white), which in turn are depleted as the spindle approaches (right, microtubules in black). A magenta arrow points at astral-microtubules emanating from the spindle pole towards a patch of LGN.

C – X-Z section of a confocal time-lapse of a representative HeLa cell on a FN-coated unpatterned substrate treated with Rap1* + STLC. Centrosomes lie close to the basal membrane where LGN is concentrated.

D – Representative confocal time-lapse of a HeLa cell on a FN-coated unpatterned substrate with a monopolar spindle that stopped moving. LGN accumulates in regions far from the spindle but does not diffuse. The green dashed line indicates a region of interest used in **E**

E – Plots of LGN normalised intensity in cells where monopolar spindles stopped moving and allowed for monitoring the accumulation of LGN in space and time. The horizontal axis represents the distance from a peak of LGN (e.g. the green dashed line in **D**). Over time LGN increases in specific regions of the cell but does not diffuse rapidly (n = 5). Red lines indicate median values.

F – Confocal time-lapse of a HeLa cell on a FN-coated unpatterned substrate with a bipolar spindle that breaks up into 2 monopolar spindles that move across the cell. Cycles of LGN clearance and accumulation are locally coordinated with both monopolar spindles.

G – Plot of the normalised tubulin intensity as a function of the distance from the spindle pole in HeLa cells (as shown in Figure 2C-D, n = 8). Black dots and bars represent mean and standard error from bootstrap respectively.

H – Plot of the DNA's mean LGN inhibition range (n = 29), as a function of the threshold (corresponding to a specific quantile of the normalised LGN intensity distribution) used to

define “high levels of LGN” (see Methods S1). The threshold (0.975) and corresponding mean inhibition range are indicated by dashed green lines. The corresponding full histogram of the DNA’s LGN inhibition range is shown in Figure 2G.

I – Time-lapse confocal images of representative HeLa cells treated with Rap1* + STLC (monopolar flat) in 3 conditions: (top two rows) no additional treatment with DNA moving across the cell, (middle) no additional treatment with no DNA movements across the cell, and (bottom) additional nocodazole treatment. Cells are expressing tubulin-GFP and H2B-mCherry.

J – Confocal time-lapse of a monopolar spindle (top and middle montage, green, and bottom montage, cyan) moving in a representative HeLa cells on FN-coated unpatterned substrates. LGN (top montage, grey, and bottom montage, green) undergoes cycles of accumulation and clearance that are tightly coordinated with spindle position, while actin (middle montage, grey, and bottom montage, magenta) does not.

K – Confocal time-lapses of monopolar spindles in cells on FN-coated unpatterned substrates before and after treatments perturbing the actin cortex. Compared to the control treatment (top), latrunculin B treatment (middle) leads to a large relocalisation of LGN from the cortex to the centrosome. This accumulation is astral-microtubule dependent, as it is prevented by simultaneously treating cells with latrunculin B and low doses of nocodazole (bottom).

L – Average spindle velocities in cells treated with Y27632 (as in **M**) are similar to control cells (p-value > 0.05, Mann-Whitney *U* test). Grey bars represent median values.

M – Confocal time-lapses of monopolar spindles in 2 representative HeLa cells on FN-coated unpatterned substrates before and after Y27632 treatment. Spindle velocity is not affected by ROCK inhibition.

All scalebars indicates 10 µm

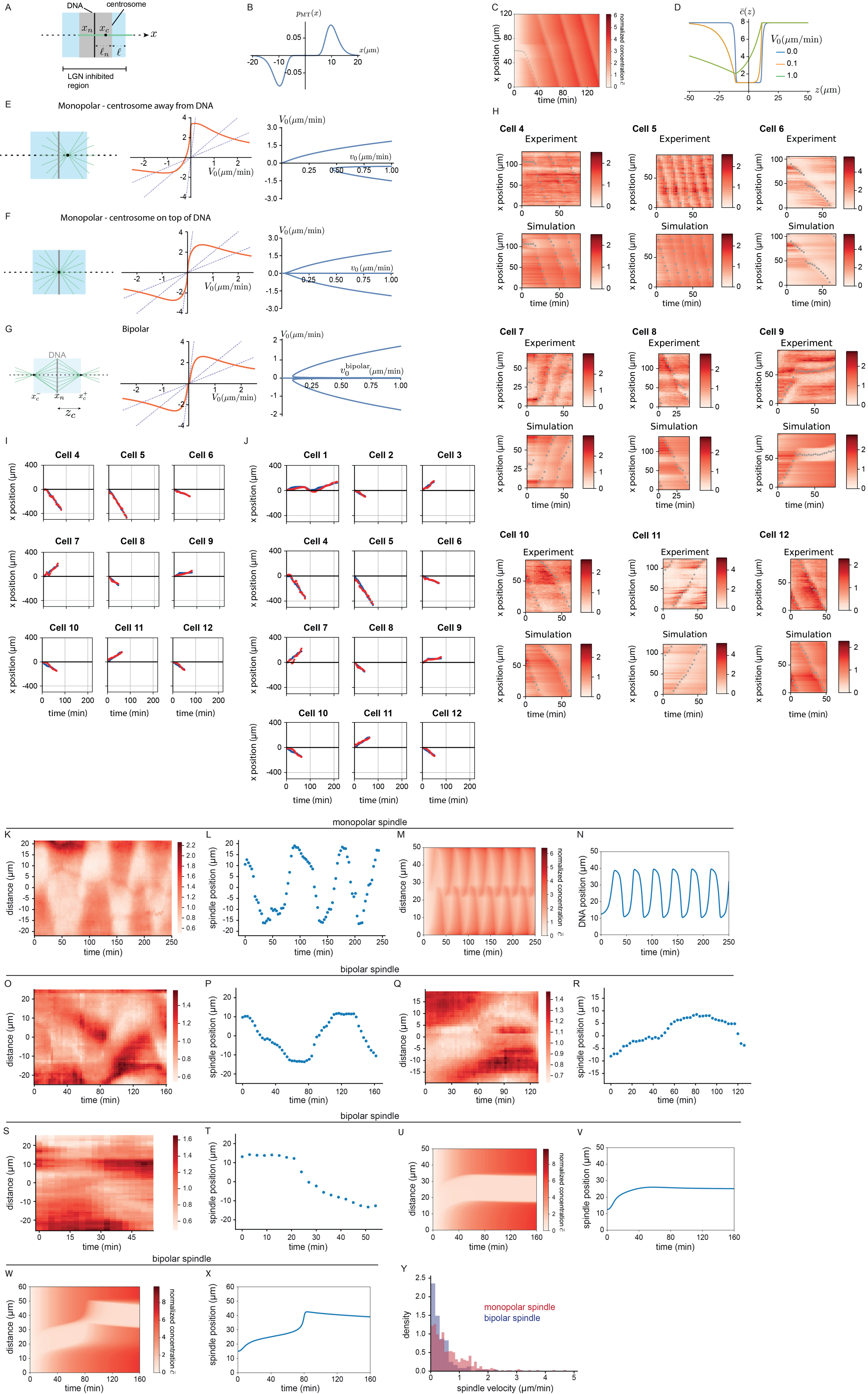

A
DNA
centrosome
x_n
x_c
ℓ_n
ℓ
x
LGN inhibited region
B
$p_{\mathrm{MT}}(x)$
$x(\mu m)$
C
x position (µm)
time (min)
normalized concentration $\bar{c}$
D
$\bar{c}(z)$
$V_0(\mu m/min)$
0.0
0.1
1.0
$z(\mu m)$
E
Monopolar - centrosome away from DNA
$V_0(\mu m/min)$
$v_0(\mu m/min)$
F
Monopolar - centrosome on top of DNA
$V_0(\mu m/min)$
$v_0(\mu m/min)$
G
Bipolar
DNA
x_c^-
x_n
x_c^+
z_c
$V_0(\mu m/min)$
$v_0^{\mathrm{bipolar}}(\mu m/min)$
H
Cell 4
Cell 5
Cell 6
Cell 7
Cell 8
Cell 9
Cell 10
Cell 11
Cell 12
Experiment
Simulation
x position (µm)
time (min)
I
J
Cell 1
Cell 2
Cell 3
x position (µm)
time (min)
monopolar spindle
K
L
M
N
distance (µm)
time (min)
spindle position (µm)
normalized concentration $\bar{c}$
DNA position (µm)
bipolar spindle
O
P
Q
R
distance (µm)
spindle position (µm)
time (min)
bipolar spindle
S
T
U
V
distance (µm)
spindle position (µm)
normalized concentration $\bar{c}$
time (min)
bipolar spindle
W
X
Y
distance (µm)
normalized concentration $\bar{c}$
spindle position (µm)
time (min)
density
monopolar spindle
bipolar spindle
spindle velocity (µm/min)

Figure S3 – Analysis of the one-dimensional model of monopolar and bipolar spindle motion and LGN concentration in cells on unpatterned substrates and on patterned lines. Related to Figure 3.

A – Schematic of geometrical quantities in the one-dimensional model of spindle motion. Black line: DNA, black dot: centrosome, green line: microtubules emanating from the centrosome. x_n is the position of the DNA, x_c the position of the centrosome, ℓ_n half the width of the DNA, and ℓ is the distance away from the DNA up to which the cortical LGN unbinding rate is enhanced.

B – Signed distribution of astral microtubule end density, obtained from a fit to experimental data (Figure 2D). The sign of the distribution corresponds to the orientation of microtubules.

C – LGN concentration and spindle motion in the full mechano-chemical feedback model, when the centrosome and DNA centre are at the same location ($x_c = x_n$). Colours are as in Figure 3E. The initial condition for the concentration profile is homogeneous with a small random perturbation. A symmetry-breaking instability leads to spontaneous motion of the DNA.

D – Steady-state profiles of theoretical LGN concentration, for positions z relative to the DNA, at different DNA velocities and for an infinite domain (see Methods S1). Different colours correspond to different velocities of the DNA (blue: $V_0 = 0$, orange: $V_0 = 0.1\ \mu\text{m/min}$, green: $V_0 = 1\ \mu\text{m/min}$). Other parameters are listed in Table I in Methods S1. DNA movement is assumed to be directed towards positive values of z. As the DNA velocity is increased, a region of inhibited LGN concentration extends behind the DNA.

E – Left: schematic of centrosome, DNA and microtubule organization, when the centrosome and DNA have different positions. Middle: a self-consistent equation (Eq. 15 in Methods S1) determines the DNA-centrosome velocity V_0 at the intersection between the red curve and blue lines (corresponding to v_0=0.05 $\mu\text{m/min}$, v_0=0.5 $\mu\text{m/min}$, v_0=1 $\mu\text{m/min}$, from the largest to smallest slope). Right: DNA velocity V_0 as a function of the characteristic velocity v_0, as determined from the self-consistent equation. A moving solution exists for all non-zero values of v_0, with the velocity of the DNA oriented towards the centrosome. For high enough values of v_0, two other solutions emerge with an opposite orientation of velocity.

F – Left: schematic of centrosome, DNA and microtubule organization, when the centrosome and DNA are at the same position. Middle: A self-consistent equation determines the DNA-centrosome velocity V_0 at the intersection between the red curve and blue lines (corresponding to v_0=0.05 $\mu\text{m}/\text{min}$, v_0=0.5 $\mu\text{m}/\text{min}$, v_0=1 $\mu\text{m}/\text{min}$, from the largest to smallest slope). Right: DNA velocity V_0 as a function of the characteristic velocity v_0, as determined from the self-consistent equation. For a large enough value of v_0, two moving solutions with velocities in opposite direction emerge. In our analysis of monopolar spindle, we obtain $v_0 = 6.7 \pm 6.7\ \mu\text{m}/\text{min}$, with an average value above the threshold for spontaneous motion.

G – Left: schematic of centrosome, DNA and microtubule organization, for a bipolar spindle. Middle: A self-consistent equation determines the bipolar spindle velocity V_0 at the intersection between the red curve and blue lines (corresponding to v_0^{bipolar}=0.05 $\mu\text{m}/\text{min}$, v_0^{bipolar}=0.5 $\mu\text{m}/\text{min}$, v_0^{bipolar}=1 $\mu\text{m}/\text{min}$, from the largest to smallest slope). Right: bipolar spindle velocity V_0 as a function of the characteristic velocity v_0^{bipolar}, as determined from the self-consistent equation. For a large enough value of v_0^{bipolar}, moving solutions with velocities in opposite direction emerge.

H – Top panels, experimental kymographs showing the cortical LGN fluorescence intensity over space and time (red colours), and the DNA position over time (grey dots) for 9 cells analysed, in addition to 3 cells shown in Figure 3B. Bottom panels, corresponding kymographs of simulated cortical LGN concentration, obtained by imposing the experimentally measured DNA position.

I – Distance travelled by the DNA, experimentally measured (blue) and predicted from the equation given in Figure 3C, for 9 analysed cells, in addition to 3 cells shown in Figure 3D. The value of the proportionality coefficient v_0 is chosen independently for each cell.

J – As in **I**, distance travelled by the DNA, experimentally measured (blue) and predicted from the equation given in Figure 3C, for all 12 analysed cells, but for a different choice of distribution of force acting on microtubules. Instead of assuming that forces act on the end of microtubules, as in **I**, one assumes here that forces act on the entire microtubule length (see Methods S1).

K – Kymograph (space-time representation) of LGN concentration over time in a HeLa cell treated with Rap1* + STLC (with a monopolar spindle) on a line pattern.

L – Position over time of the monopolar spindle in the HeLa cell shown in **K.**

M – Kymograph of simulated cortical LGN concentration, with no-flux boundary conditions for LGN at both ends of the one-dimensional domain, for a monopolar spindle, when the centrosome and DNA centre are at the same location ($x_c = x_n$). Parameters are as given in Table 1, except for the cell size which is $L_c = 50$μm. Initial conditions are set to $\bar{c} = 0$ (no cortical LGN) and the DNA position is offset from the cell centre (initial position $L_c/4$).

N – Position over time of DNA centre for the simulation shown in **M**. The DNA exhibits spontaneous oscillations.

O, Q, S – Kymograph (space-time representation) of LGN concentration over time in a HeLa cell treated with Rap1* (with a bipolar spindle) on a line pattern.

P, R, T – Position over time of the bipolar spindle in the HeLa cell shown in **O, Q, S.**

U, W – Kymograph of simulated cortical LGN concentration, with no-flux boundary conditions for LGN at both ends of the one-dimensional domain, for a bipolar spindle. Parameters are as given in Table 1 or in section 1D-E of the Methods S1, and the domain size is $L_c = 50$ μm (U) or $L_c = 60$ μm (W). Initial conditions are set to $\bar{c} = 0$ (no cortical LGN) and the DNA position is offset from the cell centre (initial position $L_c/4$ with L_c the domain size).

V, X – Position over time of DNA centre for the simulation shown in **U**, **W**. In **V** the spindle converges to the cell centre, while it starts an oscillatory movement in **W**.

Y – Histograms of spindle velocities for monopolar (n = 5 cells) and bipolar (n = 4 cells) spindles in HeLa cells on line patterns (representative plots shown respectively in **L,** and **P-R-T**).

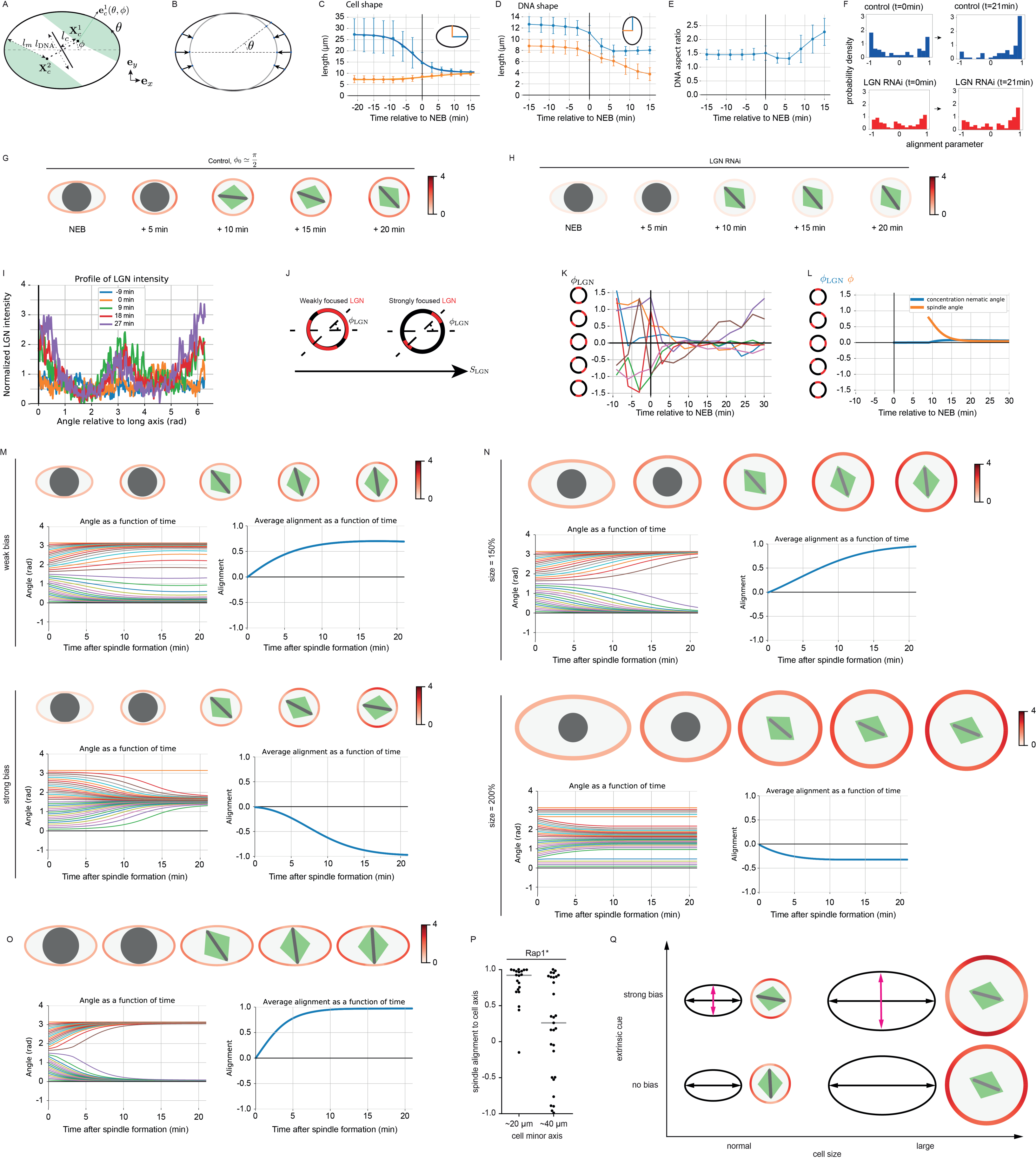

A
B
C
Cell shape
length (µm)
Time relative to NEB (min)
D
DNA shape
length (µm)
Time relative to NEB (min)
E
DNA aspect ratio
Time relative to NEB (min)
F
control (t=0min)
control (t=21min)
LGN RNAi (t=0min)
LGN RNAi (t=21min)
probability density
alignment parameter
G
Control, $\phi_0 \simeq \frac{\pi}{2}$
NEB
+ 5 min
+ 10 min
+ 15 min
+ 20 min
H
LGN RNAi
NEB
+ 5 min
+ 10 min
+ 15 min
+ 20 min
I
Profile of LGN intensity
-9 min
0 min
9 min
18 min
27 min
Normalized LGN intensity
Angle relative to long axis (rad)
J
Weakly focused LGN
Strongly focused LGN
ϕ_{LGN}
S_{LGN}
K
ϕ_{LGN}
Time relative to NEB (min)
L
ϕ_{LGN} ϕ
concentration nematic angle
spindle angle
Time relative to NEB (min)
M
weak bias
strong bias
Angle as a function of time
Angle (rad)
Time after spindle formation (min)
Average alignment as a function of time
Alignment
N
size = 150%
size = 200%
Angle as a function of time
Angle (rad)
Time after spindle formation (min)
Average alignment as a function of time
Alignment
O
Angle as a function of time
Angle (rad)
Time after spindle formation (min)
Average alignment as a function of time
Alignment
P
Rap1*
spindle alignment to cell axis
~20 µm
~40 µm
cell minor axis
Q
strong bias
no bias
extrinsic cue
normal
large
cell size

Figure S4 – Two-dimensional model of bipolar spindle motion and LGN cortical distribution in simulations and experiments, and the effect of bias of cortical cues and cell size on the model. Related to Figure 4.

A – Schematic of geometrical parameters in a two-dimensional model of bipolar spindle motion.

B – Schematic of cell rounding. In simulations, we assume for simplicity that points on the cell outline follow trajectories of constant polar angle θ as mitotic rounding occurs.

C – Fit to quantification of cell shape changes during mitotic rounding, for mitotic HeLa cells cultured on FN-coated micropatterned lines. Blue: long cell axis half-length, Orange: short cell axis half-length. Dots: mean of experimental data for n=33 cells, error bars: standard deviation, solid lines: fit to Eqs. 37-38 in Methods S1.

D - DNA shape changes around NEB in HeLa cells on FN-coated unpatterned substrates, as a function of time. Blue: DNA semi long axis length, Orange: DNA semi short axis length. Dots are mean of experimental data for n=44 cells, error bars are standard deviation.

E – DNA aspect ratio around NEB in HeLa cells on FN-coated unpatterned substrates, as a function of time. The aspect ratio is defined as the long axis length divided by the short axis length. Error bars: standard deviation for n=44 cells.

F – Distribution of experimentally measured spindle alignment along the long cell axis in mitotic HeLa cells cultured on micropatterned lines, with either control (n=94) or LGN-RNAi (n=96) treatment, at the time of spindle formation (left) and 21 minutes after spindle formation (right).

G – Cell shape, DNA and spindle angle, and LGN concentration at different times after NEB, in a simulation of mitotic rounding and spindle rotation in control conditions, for an initial spindle angle ϕ away from the long axis close to $\pi/2$ ($\phi_0 = \pi/2$ - $\pi/40$). Simulation time points and colour codes are as in Figure 4I.

H – Cell shape, DNA and spindle angle, and LGN concentration at different times after NEB, in a simulation of mitotic rounding and spindle rotation in LGN-RNAi conditions, for an initial angle $\phi_0 = \pi/4$. Simulation time points and colour codes are as in Figure 4I.

I – Quantification of LGN fluorescence intensity profile on the cell contour, at different times, from one representative cell shown in Figure 4A. Angles are taken relative to the long cell axis measured at time = NEB – 9 minutes.

J – Schematic of nematic order parameter calculated from the distribution of LGN concentration around the cell contour. S_{LGN} is the nematic order magnitude which quantifies how strongly the concentration is focused on opposite poles in the cell (see Methods S1 for a definition). ϕ_{LGN} is the nematic angle which quantifies the orientation of the axis of bipolar distribution of LGN.

K – Nematic angle ϕ_{LGN} as a function of time for different cells (n=7), for which Figure 4A is a representative example.

L – Nematic angle ϕ_{LGN} (blue curve) and spindle angle ϕ (orange curve) as a function of time, for the simulation shown in Figure 4I. In the simulation LGN orients along the long axis after NEB, and a slight loss of LGN orientation occurs after spindle formation, due to the anisotropic shape of DNA and slight misalignment of the spindle.

M – Simulation results with increasing bias along the vertical axis arising from an external cue. A bias is introduced by assuming a spatial profile of LGN binding rate $k_{\mathrm{on}}(\theta)$ with a maximum k_{on}^{+} at $\theta = \pm\frac{\pi}{2}$ and a minimum k_{on}^{-} at $\theta = 0,\ \pi$ (see Methods S1 for a more precise description). Top graphs: weak bias $k_{\mathrm{on}}^{+} \simeq 1.45\ k_{\mathrm{on}}^{-}$; bottom graphs: strong bias $k_{\mathrm{on}}^{+} = 4\ k_{\mathrm{on}}^{-}$. For each case: Top images depict simulation outputs, with time points and colour codes as in Figure 4I; bottom left graphs show predicted spindle orientation trajectories as a function of time, as in Figure 4J; bottom right graphs show the average alignment of the spindle angle with the initial cell elongation axis, as a function of time, as in Figure 4L. A strong enough bias polarizes LGN along the vertical cell axis, leading to a force distribution that orients the spindle axis perpendicular to the cell elongation axis. Negative alignment values correspond to spindle orienting along the vertical axis, with -1 corresponding to perfect vertical alignment.

N – Simulation results with increasing cell size. The cell shape is isotropically scaled by a constant factor (see Methods S1 for a more precise description). Top graphs: cell size is increased by 1.5 compared to Figure 4I; bottom graphs: cell size is increased by 2. For each case: Top images depict simulation outputs, with time points and colour codes as in Figure 4I; bottom left graphs show predicted spindle orientation trajectories as a function

of time, as in Figure 4J; bottom right graphs show the average alignment of the spindle angle with the initial cell elongation axis, as a function of time, as in Figure 4L. When the cell becomes too large, astral microtubules have reduced contacts with the cortex, leading to loss of spindle alignment along the long axis.

O - Simulation result with constant elongated cell shape (see Methods S1 for a more precise description). Top images depict simulation outputs, with time points and colour codes as in Figure 4I; bottom left graph shows predicted spindle orientation trajectories as a function of time, as in Figure 4J; bottom right graph shows the average alignment of the spindle angle with the initial cell elongation axis, as a function of time, as in Figure 4L. In this example the spindle orients on average along the long cell axis.

P – Plot showing the alignment between the mitotic spindle and the cell axis (measured at the timepoint before anaphase onset) in Rap1* cells with a small width (on FN-coated line patterns, minor axis length 22 µm ± 6 µm; major axis length 65 µm ± 17 µm), and with a larger width (on FN-coated unpatterned substrates, minor axis length 37 µm ± 7 µm; major axis length 59 µm ± 10 µm). Grey bars represent median values.

Q – Schematic of the effect of the bias of cortical cues and of cell size on spindle alignment with the cell axis. Black ellipses show interphase cells shape, black lines and arrows show the interphase cell axis, and the magenta line and arrows represent the extrinsic cue bias. In control HeLa cells (where weak or no extrinsic bias of cortical cues are present), the spindle aligns to the interphase cell axis through the mechano-chemical interaction between astral-microtubules and cortical motors. When a strong extrinsic cue is present, spindle alignment can be overridden to ignore interphase cell shape. In large cells, microtubules and DNA-mediated inhibition do not scale with cell size, thus the spindle cannot align to the interphase elongation cell axis.
