# Supplementary material for: Mechanochemical Crosstalk Produces Cell-Intrinsic Patterning of the Cortex to Orient the Mitotic Spindle
Source: Curr Biol. 2020 Sep 21;30(18):3687–3696.e4. doi: 10.1016/j.cub.2020.06.098 (PMC7521479; doi:10.1016/j.cub.2020.06.098)
Supplement: Methods S1. Dynamic equations for monopolar spindle motion in flat mitotic cells and for bipolar spindle orientation in cells undergoing mitotic rounding, related to STAR Methods [file mmc2.pdf]

Methods S1

In this supplementary we describe a model of spindle orientation that takes into-account a cross-talk between the spindle and the dynamics of LGN at the cell cortex. We first discuss a one-dimensional model for monopolar spindle motion. We describe dynamic equations for the motion of the monopolar spindle, driven by cortical forces acting on microtubules, and for the LGN cortical concentration profile which depends on the position of the DNA in the cell. We then discuss a model incorporating a similar feedback, but in a setting where a bipolar spindle orients itself within a cell undergoing mitotic rounding.

I. ONE-DIMENSIONAL MOTION OF MONOPOLAR SPINDLE

In this section we describe the motion of the DNA and centrosome in a periodic one-dimensional stripe $0 < x < L_c$, representing monopolar spindle motion on a roughly circular trajectory (Fig. 3A).

A. General equations

Here we denote by c the concentration profile of cortical LGN. We assume that the concentration of cortical LGN follows a simple reaction-diffusion equation:

$$\partial_t c = D\partial_x^2 c + k_{\mathrm{on}} - k_{\mathrm{off}}(x, x_n)c \ , \tag{1}$$

where D is the diffusion coefficient of LGN on the cortex, k_{on} is the binding rate of cytoplasmic LGN to the cortex, and $k_{\mathrm{off}}(x, x_n)$ is the off rate of LGN from the cortex. We assume here that k_{on} is constant, and $k_{\mathrm{off}}(x, x_n)$ is a function of space and the position of the center of the DNA, x_n. We assume that inhibition of LGN binding by a gradient of Ran-GTP away from the DNA is influencing the off rate k_{off}. For simplicity we consider the gradient of Ran-GTP gradient away from the DNA to be quasi-static compared to DNA motion [1]. We therefore capture the effect of Ran-GTP inhibition by the simple functional form for the LGN off-rate:

$$k_{\mathrm{off}}(x, x_n) = \begin{cases} k_{\mathrm{off}}^{\mathrm{near}}, |x - x_n| < \ell_n + \ell \\ k_{\mathrm{off}}^{\mathrm{far}}, |x - x_n| \geq \ell_n + \ell \end{cases} , \tag{2}$$

where ℓ_n represent the half-width of the DNA and ℓ the LGN inhibition length arising from the Ran-GTP gradient. We expect $k_{\mathrm{off}}^{\mathrm{near}} > k_{\mathrm{off}}^{\mathrm{far}}$. In practice we use periodic boundary conditions and the distance $|x - x_n|$ in Eq. 2 is determined as the shortest distance for x, x_n in different periodic boxes.

The DNA is assumed to move according to the force balance equation:

$$\gamma \partial_t x_n = F = \int dx f_0 c(x) n_{\mathrm{MT}}(x - x_c) \ , \tag{3}$$

where x_c is the position of the centrosome. In Eq. 3, γ is an effective friction coefficient acting against motion of the DNA, F is the total force exerted on the DNA, aside from the friction force. We assume here that the force F arises from dyneins exerting forces at microtubules end, in a LGN-dependent manner. $n_{\mathrm{MT}}(x - x_c)$ is a signed number density of microtubule ends at distance x from the DNA, where the sign indicates microtubule orientation. We use periodic boundary conditions on a domain of size L_c, and we assume that that the characteristic decay length of $n_{\mathrm{MT}}(x - x_c)$ away from x_c is small compared to L_c. f_0 is the force exerted by dynein molecular motors on the spindle per microtubule end and per LGN molecule density; such that $f_0 c(x)$ is the force exerted by dyneins on one microtubule end at location x.

To estimate $n_{\mathrm{MT}}(x - x_c)$, we use experimental measurements of microtubule end density away from the centrosome (Fig. 2D). We fit the experimentally measured microtubule end probability density $p_{\mathrm{MT}}^{+}(x)$ for $x > 0$ by a log-normal function. As a result, we define a signed normalized density microtubule ends, $p_{\mathrm{MT}} = n_{\mathrm{MT}}/N_{\mathrm{MT}}$, where N_{MT} is the total number of microtubules ends on both sides of the centrosome, by

$$p_{\mathrm{MT}}(x - x_c) = \frac{1}{2} \begin{cases} \frac{1}{|x - x_c|\sigma\sqrt{2\pi}} e^{-\frac{(\log|x - x_c| - \mu)^2}{2\sigma^2}} & x > x_c \\ -\frac{1}{|x - x_c|\sigma\sqrt{2\pi}} e^{-\frac{(\log|x - x_c| - \mu)^2}{2\sigma^2}} & x < x_c \end{cases} , \tag{4}$$

with $e^{\mu} = 10.28\mu$m and $e^{\sigma} = 1.26$ (Fig. S3B). Here we have assumed that microtubules ends have the same probability to be on each side of the centrosome, and the position of the centrosome is denoted x_c (Fig. S3A). The changing sign of the function p_{MT} is introduced to take into account the direction of microtubules. In the following we assume that the distance $x_n - x_c$ is fixed, and the distribution defined in Eq. 4 is implemented taking into account periodic boundary conditions on a domain of size L_c, and that the decay length of the distribution is small compared to L_c.

We now denote $c_0 = k_{\mathrm{on}}/k_{\mathrm{off}}^{\mathrm{near}}$ the equilibrium concentration near the DNA, and introduce the rescaled concentration $\bar{c} = c/c_0$. The dynamical equations 1 and 3 can then be rewritten

$$\partial_t \bar{c} = D\partial_x^2 \bar{c} + \frac{1}{\tau} - k_{\mathrm{off}}(x, x_n)\bar{c} \tag{5}$$

$$\partial_t x_n = v_0 \int dx \bar{c}(x) p_{\mathrm{MT}}(x - x_c) \ , \tag{6}$$

where $\tau = 1/k_{\mathrm{off}}^{\mathrm{near}}$ is the time scale of LGN cortical unbinding near the DNA, and

$$v_0 = \frac{f_0 N_{\mathrm{MT}} c_0}{\gamma} \tag{7}$$

is a characteristic DNA velocity.

B. Fitting to experimental data

In order to fit the model described by Eqs. 5-6 to experimental data of monopolar spindles, we proceeded as follows:

- The radius of the DNA was estimated by fitting an ellipse to the DNA shape, and measuring its major and minor axes. This gives a major axis $20 \pm 3\mu$m and a minor axis $14.7 \pm 1.9\mu$m (mean ± standard deviation, n=15 cells, Fig. S1D). In the one-dimensional model we take the half-width of the DNA to be half the minor axis, $\ell_n \simeq 7\mu$m.
- The length of LGN inhibition ℓ away from the center of the DNA was estimated as follows. Cell images were recorded when by chance, the DNA was stationary and the LGN profile in the cell was not visibly changing (Fig. 2F). We then determined the distance between the contour of the DNA and the closest region of high LGN intensity, where high LGN intensity region were thresholded from the original images by retaining pixels with values of fluorescence intensity above quantile Q We found that this distance was of the order of a few μms, with a precise value that was sensitive to the choice of Q (Fig. S2H). Although this analysis sets the order of magnitude of the inhibition length, to obtain a specific value for simulations, we chose a quantile of 0.975 relative to the distribution of fluorescence intensities, giving an inhibition length of $\sim 4\mu$m. The need to choose a specific value is borne out by our simplifying assumption that LGN is inhibited uniformly up to a fixed distance from the DNA. We note however that we expect our results to be weakly sensitive to a small change in this inhibition length, as long as it matches with the short and long axis dimensions of the cell to correctly pattern the cortex (Fig. 4I).
- We postulated a diffusion constant $D = 0.01\mu\mathrm{m}^2/\mathrm{min}$, resulting in a small effect of diffusion of LGN compared to binding and unbinding to the cytoplasm. This is consistent with the experimentally observed stability of local accumulations of LGN far from the spindle (Fig. S2D-E).
- We fitted numerical solutions of Eq. 5 to experimental spatiotemporal profiles of cortical LGN, taking the position of the DNA x_n from experimental measurements. This yielded values for the off-rates $k_{\mathrm{off}}^{\mathrm{far}}$, $k_{\mathrm{off}}^{\mathrm{near}}$. Specifically, we fitted the following objective function:

$$S = \left[\sum_{\text{cell } \alpha} \left[\sum_{t_i} \sum_{x_j} \left(\frac{c^{\mathrm{sim},\alpha}(t_i, x_j)}{\langle c^{\mathrm{sim},\alpha} \rangle} - \frac{c^{\mathrm{exp},\alpha}}{\langle c^{\mathrm{exp},\alpha} \rangle} \right)^2 \right] \right]^{\frac{1}{2}} \ , \tag{8}$$

  where t_i and x_j are the experimentally measured time and space points, $\langle c \rangle = \frac{1}{N_t N_x} \sum_{t_i} \sum_{x_j} c$ is the average concentration over time and space, $c^{\mathrm{sim},\alpha}$ is the simulated concentration for cell α and $c^{\mathrm{exp},\alpha}$ the experimentally measured LGN fluorescence intensity of cell α. To obtain simulation results, we needed to set an initial condition

for the concentration profile of LGN. This initial condition was determined by running $n = 1...N$ simulations iteratively with initial condition for simulation n

$$c^{\text{sim},\alpha,1}(t=0) = \frac{c^{\text{exp},\alpha}(t=0)}{\langle c^{\text{exp},\alpha}\rangle}$$
$$c^{\text{sim},\alpha,n+1}(t=0) = \langle c^{\text{sim},\alpha,n}\rangle \frac{c^{\text{exp},\alpha}(t=0)}{\langle c^{\text{exp},\alpha}\rangle}, \quad n \geq 1, \tag{9}$$

which converges to $c^{\text{sim},\alpha}(t=0)/\langle c^{\text{sim},\alpha}\rangle = c^{\text{exp},\alpha}(t=0)/\langle c^{\text{exp},\alpha}\rangle$ for large n. In the fitting procedure we chose $N = 8$, as we found that with this number the last two iterations led to a relative variation of the objective function $< 1.4 \cdot 10^{-3}$ for all tested parameters.

- For different time points and different cells, we calculated from simulations adjusted to experimental measurements of LGN concentration, the integral $\int dx\bar{c}(x)p_{\text{MT}}(x-x_c)$. We then integrated Eq. 6 to obtain a predicted DNA displacement $\Delta x_n^{\text{predicted}}$. We then estimated v_0 for each cell by adjusting the predicted DNA displacement to the experimentally measured DNA displacement Δx_n^{exp} (Figs. 3D and S3I). We found $v_0 = 6.7 \pm 6.7\mu$m/min with v_0 evaluated in 12 cells.

We find that our results for the evaluation of v_0 and the relation between LGN intensity and DNA velocity are not strongly sensitive to a particular choice for the distribution p_{MT}. To show this, we have tested a different distribution where the force is taken proportional to the microtubule density, instead of the microtubule end density (Fig. S2G). We then use the function

$$p_{\text{MT}}(x-x_c) = \frac{1}{2}\begin{cases} I_{\text{MT}}(|x-x_c|) & x > x_c \\ -I_{\text{MT}}(|x-x_c|) & x < x_c \end{cases} \tag{10}$$

$$I_{\text{MT}}(x) = \frac{1}{d_0 + d_{\text{MT}}}\begin{cases} 1 & x < d_0 \\ e^{-\frac{x-d_0}{d_{\text{MT}}}} & x > d_0 \end{cases}, \tag{11}$$

where $d_0 \simeq 1.7\mu$m and $d_{\text{MT}} \simeq 4.8\mu$m are determined using a fit to experimental data of tubulin-GFP intensity away from the spindle pole; in practice we fitted a function $I_\infty + I_0(d_0 + d_{\text{MT}})I_{\text{MT}}(x)$ to experimental data where I_0 and I_∞ are two additional fitted constants. This different choice of force distribution applied on microtubules still yields a good agreement between the predicted and experimental DNA velocity (Fig. S3J) for a set of values of v_0 which verify $v_0 = 15.3 \pm 15.4\mu$m/min (mean± std).

Parameter	Name	Value	Unit
DNA half-width	ℓ_n	~ 7	μm
Ran-GTP inhibition length	ℓ	4	μm
DNA-centrosome distance	$\lvert x_c - x_n\rvert$	4.9	μm
LGN diffusion coefficient	D	0.01	μm^2/min
LGN unbinding timescale near the DNA	τ	11.5	min
Ratio of LGN off rates away and near the DNA	$\frac{k_{\text{off}}^{\text{far}}}{k_{\text{off}}^{\text{near}}}$	0.1265	
Characteristic DNA velocity	v_0	6.7 ± 6.7	μm/min

TABLE I: Parameter values for model of monopolar spindle motion. The DNA half width and DNA-centrosome distance are obtained from Fig. S1D. The LGN unbinding timescale and ratio fo LGN off rates are obtained from a fit to spatiotemporal profiles of cortical LGN in monopolar spindle experiments. The characteristic DNA velocity is obtained from a fit to the DNA displacement in monopolar spindle experiments.

In Fig. 3E we show results of simulation using Eqs. 5 and 6, with parameters listed in Table I. The centrosome is positioned to the left of the DNA, $x_c = x_n - |x_c - x_n|$, and the initial LGN cortical concentration is set to 0. The distance between the nucleus and centrosome $|x_c - x_n|$ is set to 4.9μm, an average value from experimental measurements (Fig. S1D). In Fig. S3C we show results of simulation under the same conditions, except that the DNA and the centrosome are in the same position, $x_c = x_n$, and a small random perturbation is added to the initial LGN cortical concentration.

C. Analysis of steady-state velocity

Numerical simulations indicate that Eqs. 5 and 6 evolve towards a steady-state motion of the DNA and the LGN profile (Fig. 3E). Here we discuss the velocity of the DNA V_0 in the steady-state. We introduce the comoving coordinate $z = x - V_0 t$, and take $x_n = V_0 t$, such that the equation for the concentration profile reads at steady-state

$$V_0 \partial_z \bar{c} + D\partial_z^2 \bar{c} + \frac{1}{\tau} - k_{\text{off}}(z, 0)\bar{c} = 0 \ . \tag{12}$$

For simplicity we solve the equation in an infinite domain, corresponding to the length of the cell L_c being much larger than other characteristic lengths determining the concentration profile. Requesting that the solution does not diverge at infinity, we then obtain:

$$\bar{c}(z) = \begin{cases} C_1 e^{\frac{z}{l_+^{\text{far}}}} + \frac{k_{\text{off}}^{\text{near}}}{k_{\text{off}}^{\text{far}}} & z \leq -(\ell + \ell_n) \\ C_2 e^{\frac{z}{l_+^{\text{near}}}} + C_3 e^{-\frac{z}{l_-^{\text{near}}}} + 1 & -(\ell + \ell_n) < z < \ell + \ell_n \\ C_4 e^{-\frac{z}{l_-^{\text{far}}}} + \frac{k_{\text{off}}^{\text{near}}}{k_{\text{off}}^{\text{far}}} & z \geq \ell + \ell_n \ , \end{cases} \tag{13}$$

where we have introduced the length scales

$$\begin{aligned} l_+^{\text{far}} &= \frac{V_0}{2k_{\text{off}}^{\text{far}}} \left(1 + \sqrt{1 + 4D\frac{k_{\text{off}}^{\text{far}}}{V_0^2}}\right) \\ l_-^{\text{far}} &= \frac{2D}{V_0} \frac{1}{1 + \sqrt{1 + \frac{4Dk_{\text{off}}^{\text{far}}}{V_0^2}}} \\ l_+^{\text{near}} &= \frac{1}{2}\tau V_0 \left(\sqrt{\frac{4D}{\tau V_0^2} + 1} + 1\right) \\ l_-^{\text{near}} &= \frac{1}{2}\tau V_0 \left(\sqrt{\frac{4D}{\tau V_0^2} + 1} - 1\right) . \end{aligned} \tag{14}$$

The constants C_i, $i = 1..4$ are found from matching the concentration profiles and its first derivative at $z = -(\ell + \ell_n)$ and $z = \ell + \ell_n$. Corresponding concentration profiles are plotted in Fig. S3D for different values of the velocity V_0 and for parameters listed in Table I.

The velocity V_0 is then obtained from the force balance equation 6, which becomes the self-consistent equation in an infinite domain:

$$\frac{V_0}{v_0} = \int_{-\infty}^{\infty} dz \bar{c}(z, V_0) p_{\text{MT}}(z - z_c) \quad . \tag{15}$$

In Fig. S3E-F, we plot the left and right hand side of this equation for different values of v_0, for the cases $|x_c - x_n| = 0$ and $|x_c - x_n| = 4.9\mu$m, and look for solutions moving with constant velocity V_0. For the case $|x_c - x_n| = 0$ where the DNA and the centrosome are at the same point, the velocity $V_0 = 0$ is a solution. For sufficiently large v_0, two opposite moving solutions $\pm V_0 \neq 0$ emerge through a pitchfork bifurcation. For $|x_c - x_n| \neq 0$ the pitchfork bifurcation becomes imperfect and a positive solution $V_0 > 0$ exists for all values of $v_0 > 0$.

D. Bipolar spindle one-dimensional movement

Here we briefly discuss simulations of one-dimensional motion of a bipolar spindle. The DNA position is denoted x_n, and the positions of the two centrosomes x_c^+ and x_c^-. We take the distances between the centrosome and DNA $|x_c^+ - x_n| = |x_c^- - x_n| = z_c$ to be equal to 8μm (Fig. S3G), a value obtained from experimental measurements ($z_c = 8 \pm 1\mu$m, mean± std, n = 29 cells). Here we consider that each centrosome has astral microtubules radiating in the direction opposite from the DNA and participating to force generation on the spindle, such that the distribution

of microtubules is given by

$$p_{\rm MT}^{\rm bipolar}(z) = \frac{1}{2}\begin{cases} \frac{1}{|z-z_c|\sigma\sqrt{2\pi}} e^{-\frac{(\log|z-z_c|-\mu)^2}{2\sigma^2}} & z \geq z_c \\ 0 & -z_c < z < z_c \\ -\frac{1}{|z+z_c|\sigma\sqrt{2\pi}} e^{-\frac{(\log|z+z_c|-\mu)^2}{2\sigma^2}} & z \leq -z_c \end{cases} , \tag{16}$$

with $z = x - x_n$ the distance between a point in the cell and the DNA center.

The DNA is assumed to move according to the modified force balance equation:

$$\gamma \partial_t x_n = F = \int dx f_0 c(x) N_{\rm MT}^{\rm bipolar} p_{\rm MT}^{\rm bipolar}(x - x_n) , \tag{17}$$

where $N_{\rm MT}^{\rm bipolar}$ is the total number of microtubules participating to force generation in the bipolar case. We further assume that the LGN concentration follows Eq. 5. The analysis of section I C for the steady-state concentration around the DNA moving at constant velocity can be repeated; and solutions for the steady-state velocity are then given by

$$\frac{V_0}{v_0^{\rm bipolar}} = \int_{-\infty}^{\infty} dz \bar{c}(z, V_0) p_{\rm MT}^{\rm bipolar}(z) \quad . \tag{18}$$

with $v_0^{\rm bipolar} = f_0 N_{\rm MT}^{\rm bipolar} c_0/\gamma$. In Fig. S3G, we plot the left and right hand side of this equation for different values of $v_0^{\rm bipolar}$, and look for solutions moving with constant velocity V_0. We take here $\ell_n = 4\mu m$ (from experiments $\ell_n = 4 \pm 0.9\mu$m, mean ± std, n = 20 cells). By symmetry the velocity $V_0 = 0$ is always a solution. For a large enough value of $v_0^{\rm bipolar}$, two symmetric saddle-node bifurcations give rise to moving solutions $V_0 \neq 0$ coexisting with the solution $V_0 = 0$.

E. Simulations with no-flux boundary conditions

For completeness we have run simulations of monopolar and bipolar spindle motions in a finite one-dimensional stripe of length L_c (Fig. S3M-N for the monopolar case, Fig. S3U-X for the bipolar case). We solved Eqs. 5-6 for the monopolar case, Eqs. 5 and 17 for the bipolar case, in a finite domain. Here we impose a no-flux boundary conditions for the normalized LGN concentration $\bar{c}$ at the boundaries:

$$\partial_x \bar{c}|_{x=0} = \partial_x \bar{c}|_{x=L_c} = 0 \tag{19}$$

For simplicity we did not consider additional forces acting on the DNA near the boundaries, but simply assumed that microtubules that come in contact with the cell boundaries at $x = 0$ and $x = L_c$ do not exert a force on the spindle. Parameters are chosen as in Table 1, except for $v_0^{\rm bipolar} = v_0/2$, as we assume that in the bipolar configuration less microtubules participate to force generation on the spindle than in the monopolar case.

II. BIPOLAR SPINDLE ORIENTATION IN A CELL WITH ELLIPTIC SHAPE

We now discuss a situation where the cell shape is given by a 2D contour $\mathbf{X}(\theta)$ with θ a polar angle coordinate. In the latter we consider $\mathbf{X}(\theta)$ to be an ellipse for simplicity. We also assume that the center of the DNA is pinned in the center of the cell, $\mathbf{X}_0$. The DNA is connected by the spindle to the two centrosomes with position $\mathbf{X}_c^1$ and $\mathbf{X}_c^2$ (Fig. S4A).

A. General equations

The cortical concentration of LGN on the cell contour $c(\theta)$ follows a reaction-diffusion equation analogous to Eq. 1 in the previous section:

$$\frac{1}{\sqrt{g}} \partial_t (\sqrt{g} c) = D \Delta c + k_{\rm on} - k_{\rm off}(\theta) c , \tag{20}$$

where $\sqrt{g} = |\partial_\theta \mathbf{X}|$ is the square root of the metric tensor on the curve, and Δ is the Laplace-Beltrami operator on the curved cell contour. We assume here for simplicity that during cell shape change, material points are at fixed values of θ (Fig. S4B). The rate $k_{\rm off}(\theta)$ is now determined by the distance between the contour of the DNA and the point on the contour $\mathbf{X}(\theta)$, such that

$$k_{\rm off}(\theta) = \begin{cases} k_{\rm off}^{\rm near}, d_n(\theta) < \ell \\ k_{\rm off}^{\rm far}, d_n(\theta) > \ell \end{cases} , \tag{21}$$

where $d_n(\theta)$ denotes the closest distance between $\mathbf{X}(\theta)$ and the contour of the DNA. The shape of the DNA contour is changing with time (Fig. S4D); in simulations we take the DNA contour to be either a circle or a straight line with length $l_{\rm DNA}$ (see section II C for details). ℓ is, as in the case of the monopolar spindle, the length of LGN inhibition acting away from the DNA.

Using the same normalization for the concentration as in the previous section, $\bar{c} = c/c_0$ with $c_0 = k_{\rm on}/k_{\rm off}^{\rm near}$, we write

$$\frac{1}{\sqrt{g}} \partial_t (\sqrt{g} \bar{c}) = D \Delta \bar{c} + \frac{1}{\tau} - k_{\rm off}(\theta) \bar{c} \ . \tag{22}$$

The angle of the axis of the spindle relative to the horizontal axis (also chosen to correspond to the long axis of the cell, when the cell is not circular) is now denoted ϕ. We assume that the angle ϕ evolves according to the torque exerted on the spindle:

$$\gamma_r \partial_t \phi = \Gamma_1 + \Gamma_2 \tag{23}$$

$$= \left[\oint ds \mathbf{X}(\theta) \times \mathbf{f}_1(\theta, \phi) + \oint ds \mathbf{X}(\theta) \times \mathbf{f}_2(\theta, \phi) \right] \cdot \mathbf{e}_z \quad , \tag{24}$$

where ds is the infinitesimal length element on the contour, γ_r is a rotational friction coefficient, Γ_1 and Γ_2 are the torques acting on the spindle, arising from forces exerted on microtubules connected to the two centrosomes, respectively labelled 1 and 2. The microtubules are assumed to emanate radially from the centrosomes. $\mathbf{e}_z$ is a unit vector orthogonal to the 2D plane considered here. $\mathbf{f}_1(\theta, \phi)$ and $\mathbf{f}_2(\theta, \phi)$ are the force densities acting on the spindle from microtubules connected to the cell surface and to the centrosomes 1 and 2, taken to be equal to

$$\mathbf{f}_1(\theta, \phi) = f_0 c(\theta) n_{\rm MT}(\theta, \phi) \mathbf{e}_c^1(\theta, \phi) \tag{25}$$

$$\mathbf{f}_2(\theta, \phi) = f_0 c(\theta) n_{\rm MT}(\theta, \phi + \pi) \mathbf{e}_c^2(\theta, \phi) \quad , \tag{26}$$

which can be compared to Eq. 3. In the equation above, $\mathbf{e}_c^i(\theta, \phi) = \frac{\mathbf{X}(\theta) - \mathbf{X}_c^i}{|\mathbf{X}(\theta) - \mathbf{X}_c^i|}$ is the unit vector pointing away from the cell contour, along the direction joining the position of centrosome i $\mathbf{X}_c^i$ to the point on the surface $\mathbf{X}(\theta)$. $n_{\rm MT}(\theta, \phi)$ is the number density of microtubules ends at the cell surface, for microtubules connected to centrosome 1, and by symmetry $n_{\rm MT}(\theta, \phi + \pi)$ is the number density of microtubules ends for microtubules connected to centrosome 2. f_0 is the force exerted on the spindle per microtubule end and per LGN molecule density on the cortex. Here, we have assumed that dyneins exert forces along the axis of microtubules, in a manner independent of the length of microtubules.

For simplicity we assume that the angular density of microtubules away from the centrosome is uniform, and that microtubules only touch the cell cortex in a region sufficiently close to the centrosome, such that

$$n_{\rm MT}(\theta, \phi) = n_{a0} \Theta(l_m - |\mathbf{X}(\theta) - \mathbf{X}_c^1|) \frac{(\mathbf{X}(\theta) - \mathbf{X}_c^1) \cdot \mathbf{n}}{|\mathbf{X}(\theta) - \mathbf{X}_c^1|^2} \ , \tag{27}$$

with Θ the Heaviside step function, $\mathbf{n}$ the normal vector to the cell contour, pointing away from the cell, l_m is the maximal microtubule length, and n_{a0} is the microtubule end angular density, taken here to be uniform.

B. Explicit expressions in polar coordinates

We use here polar coordinates (r, θ) and denote $\mathbf{u}_r = \cos\theta \mathbf{e}_x + \sin\theta \mathbf{e}_y$, $\mathbf{u}_\theta = -\sin\theta \mathbf{e}_x + \cos\theta \mathbf{e}_y$. The cell shape is given by

$$\mathbf{X}(\theta) = r(\theta) \mathbf{u}_r \quad , \tag{28}$$

with tangent vector $\mathbf{e}_\theta \equiv \partial_\theta \mathbf{X} = r'(\theta)\mathbf{u}_r + r(\theta)\mathbf{u}_\theta$ and normal vector $\mathbf{n} = (r(\theta)\mathbf{u}_r - r'(\theta)\mathbf{u}_\theta)/\sqrt{r(\theta)^2 + r'(\theta)^2}$. The centrosome positions are given $\mathbf{X}_c^1 = l_c(\cos\phi \mathbf{e}_x + \sin\phi \mathbf{e}_y)$ and $\mathbf{X}_c^2 = -\mathbf{X}_c^1$. The vectors $\mathbf{e}_c^i$ are given by

$$\mathbf{e}_c^1(\theta,\phi) = \frac{1}{d_c(\theta,\phi)} \begin{pmatrix} r(\theta)\cos\theta - l_c\cos\phi \\ r(\theta)\sin\theta - l_c\sin\phi \end{pmatrix} \tag{29}$$

$$\mathbf{e}_c^2(\theta,\phi) = \mathbf{e}_c^1(\theta,\phi+\pi) \quad , \tag{30}$$

where we have introduced the function giving the distance of a point on the contour to the centrosome 1:

$$d_c(\theta,\phi) = |\mathbf{X}(\theta) - \mathbf{X}_c^1| = \sqrt{r(\theta)^2 + l_c^2 - 2r(\theta)l_c\cos(\theta-\phi)} \; . \tag{31}$$

The length element on the contour is given by $ds = \sqrt{g}d\theta = \sqrt{r(\theta)^2 + r'(\theta)^2}d\theta$. The Laplace-Beltrami operator reads $\Delta\bar{c} = (\partial_\theta^2\bar{c})/g - (\partial_\theta\bar{c})(\partial_\theta g)/(2g^2)$.

The total torque acting on the spindle is given by

$$\Gamma_1 + \Gamma_2 = \Gamma(\phi) + \Gamma(\phi+\pi) \tag{32}$$

$$\Gamma(\phi) = f_0 n_{a0} l_c \int_{\Omega(\theta,\phi)} d\theta h(\theta,\phi)c(\theta) \tag{33}$$

$$h(\theta,\phi) = \frac{r(\theta)^2 - l_c\sin(\theta-\phi)r'(\theta) - l_c r(\theta)\cos(\theta-\phi)}{d_c(\theta,\phi)^3} r(\theta)\sin(\theta-\phi) \quad , \tag{34}$$

where $\Omega(\theta,\phi)$ is the domain defined by $d_c(\theta,\phi) < l_m$, and $h(\theta,\phi)$ is a geometrical dimensionless function. In the specific case where both the shape ($r(\theta)$) and the LGN concentration profiles ($c(\theta)$) are invariant by the transformation $\theta \to \theta + \pi$, one has $\Gamma(\phi+\pi) = \Gamma(\phi)$ which further simplifies the expression of the torque. As a result, the dynamic equation for ϕ can be written

$$\tau_r \partial_t \phi = 2 \int_{\Omega(\theta,\phi)} d\theta h(\theta,\phi)\bar{c}(\theta) \; , \tag{35}$$

where $\tau_r = \gamma_r/(f_0 n_{a0} l_c c_0)$ and as introduced in the monopolar case, $c_0 = k_{\text{on}}/k_{\text{off}}^{\text{near}}$ and $\bar{c} = c/c_0$. In the case of an elliptic shape that we consider here, the function $r(\theta)$ is given by

$$r(\theta) = \frac{L_m}{\sqrt{1 - e^2\cos^2\theta}} \; , \tag{36}$$

where $2L_m$ is the length of the short axis, and $e = \sqrt{1 - L_m^2/L_M^2}$ is the eccentricity, with $2L_M$ the length of the long axis.

C. Simulation of spindle rotation during mitotic rounding

Parameter	Name	Unit	Value
Final cell shape radius	L	10.5	μm
Initial cell shape semi-major axis	L_M^0	27.3	μm
Initial cell shape semi-minor axis	L_m^0	7.1	μm
Semi-major axis inflexion time	t_{M0}	-3.1	min
Semi-minor axis inflexion time	t_{m0}	4.2	min
Characteristic time scale for semi-major axis	τ_M	6.2	min
Characteristic time scale for semi-minor axis	τ_m	12	min

TABLE II: Parameter values for cell shape changes during cell rounding, obtained from experimental measurements of mitotic rounding of cells on micropatterns (Fig. S4C).

In this section we describe simulations where the cell follows a dynamics of rounding at the same time as the spindle orients itself. We consider the following sequence of events:

- At $t = 0$ (corresponding to nuclear envelope breakdown) the cell contour has a low concentration of LGN $c(\theta) = c_0^{\text{near}}$. At subsequent times, the concentration field evolves according to Eq. 20.

- The cell shape is represented as an ellipse, whose semi-major and semi-minor axis L_M and L_m change according to

$$L_M(t) = L + (L_M^0 - L)\frac{1 - \tanh\left(\frac{t - t_{M0}}{\tau_M}\right)}{2} \tag{37}$$

$$L_m(t) = L + (L_m^0 - L)\frac{1 - \tanh\left(\frac{t - t_{m0}}{\tau_m}\right)}{2} \tag{38}$$

  where L is the final cell shape radius, and the parameters t_{m0}, t_{M0}, τ_m, τ_M, L_m^0, L_M^0 are reported in Table II. These parameters were obtained by fitting experimental measurements of cell shape changes for cells cultured on micropatterns (Fig. S4C).

- In Fig. S4D-E we plot the evolution of the DNA shape and aspect ratio measured experimentally; here we used available data obtained for cells on unpatterned substrates. Over time the space occupied by DNA decreases (Fig. S4D) and around the timescale of spindle formation ($\sim$ 9 minutes after NEB) the aspect ratio of DNA increases. For the sake of simplicity we keep only some key features of this shape change dynamics in our simulations. The shape of DNA is represented by a circle of diameter l_{DNA} for to $t < t_s = 9$min. For $t \geq t_s$, the shape of the DNA is taken as a single line orthogonal to the spindle axis, with length l_{DNA}. We take here $l_{\text{DNA}} = 14\mu m$, of the order of the experimentally measured DNA diameter for cells in wild-type conditions (Fig. S4D) and smaller than the initial minor axis of cells dividing on micropatterns (Table II).

- We assume that due to delay in spindle formation, forces start to act on the spindle for $t \geq t_s \simeq 9$min (Fig. 4A-B). At this point cell rounding is complete, the cell has a circular shape and therefore without LGN patterning, no rotation of the spindle would occur.

Other model parameters are listed in Table III. The Ran-GTP inhibition length, LGN diffusion coefficient, LGN unbinding rates are taken as in simulations of monopolar spindles. The maximal length reached by astral microtubules l_m is determined from the 95% percentile of the astral microtubule length distribution determined in Fig. 2D. The distance between the centrosomes and the spindle center, l_c, is determined from experimental measurements (Fig. S1B). To estimate the spindle mobility timescale τ_r, we compare Eqs. 3 and 23 which describe respectively translational and rotational motion of the spindle. Assuming that viscous forces acting on monopolar and bipolar spindles are not very different, we expect in general $\gamma_r \sim \gamma l_s^2$, which l_s a characteristic spindle length scale. Taking $l_s = l_c$, we then expect $\tau_r \sim 4\pi l_c/v_0$, taking $n_{a0} = N_{\text{MT}}/2/(2\pi)$ with N_{MT} the total number of astral microtubules in the cell. With the values of l_c and v_0 reported in Tables I and III, we obtain the order of magnitude $\tau_r \sim 10$ minutes. In the simulations we chose $\tau_r = 12$ minutes, which leads to the simulated dynamics of spindle reorientation similar to the experimental one.

Parameter	Name	Unit	Value
Ran-GTP inhibition length	ℓ	4	μm
LGN diffusion coefficient	D	0.01	μm^2/min
LGN unbinding timescale near the DNA	τ	11.5	min
Ratio of LGN off rates away and near the DNA	$\frac{k_{\text{off}}^{\text{far}}}{k_{\text{off}}^{\text{near}}}$	0.1265	
Length of DNA	l_{DNA}	14	μm
Centrosome distance away from the spindle center	l_c	5.6	μm
Maximal microtubule length	l_m	15	μm
Spindle rotation mobility time scale	τ_r	12	min

TABLE III: Parameter values for model of bipolar spindle motion

D. Comparison to experiments

1. Definition of alignment, nematic order

For a given probability distribution $p(\phi)$ with ϕ the spindle angle with respect to the long axis and $0 \leq \phi < 2\pi$, a parameter of alignment to the cell long axis can be defined by

$$a = \int_0^{2\pi} d\phi p(\phi) \cos 2\phi \ , \tag{39}$$

such that $a = 0$ for a uniform distribution of angle ϕ, $a = 1$ when the distribution is infinitely peaked at $\phi = 0$ and $\phi = \pi$, corresponding to perfect alignment, and $a = -1$ when the distribution is peaked at $\phi = \pm\frac{\pi}{2}$, corresponding to perfect alignment orthogonal to the long axis of the cell.

When comparing LGN concentration profiles $c(\theta)$ on the cell contour, we define the associated nematic tensor Q_{ij}^{LGN} for the LGN distribution:

$$Q_{xx}^{\mathrm{LGN}} = -Q_{yy}^{\mathrm{LGN}} = \frac{\int_0^{2\pi} d\theta c(\theta) \cos 2\theta}{\int_0^{2\pi} d\theta c(\theta)} \tag{40}$$

$$Q_{xy}^{\mathrm{LGN}} = Q_{yx}^{\mathrm{LGN}} = \frac{\int_0^{2\pi} d\theta c(\theta) \sin 2\theta}{\int_0^{2\pi} d\theta c(\theta)} \ , \tag{41}$$

and the associated magnitude of nematic order S_{LGN} and nematic angle ϕ_{LGN}:

$$S_{\mathrm{LGN}} = \sqrt{\left(Q_{xx}^{\mathrm{LGN}}\right)^2 + \left(Q_{xy}^{\mathrm{LGN}}\right)^2} \tag{42}$$

$$\cos 2\phi_{\mathrm{LGN}} = \frac{Q_{xx}^{\mathrm{LGN}}}{S_{\mathrm{LGN}}} \tag{43}$$

$$\sin 2\phi_{\mathrm{LGN}} = \frac{Q_{xy}^{\mathrm{LGN}}}{S_{\mathrm{LGN}}} \ . \tag{44}$$

To solve Eqs. 43-44, we use the numpy function arctan2.

For a population of cells, an average alignment parameter of the LGN distribution with respect to the long axis can be quantified by calculating $\langle \cos 2\phi_{\mathrm{LGN}} \rangle$, with $\langle \cdot \rangle$ denoting averaging over the cell population.

2. Perturbations

Here we briefly discuss simulations of bipolar spindle rotation under perturbed conditions.

- To simulate LGN RNAi experiments, we assume that only the binding rate of LGN to the cortex is affected in this perturbation, due to the lower number of LGN molecules inside the cell in these experiments. As a result we modify the dynamic equation for LGN concentration, Eq. 22:

$$\frac{1}{\sqrt{g}} \partial_t (\sqrt{g} \bar{c}) = D \Delta \bar{c} + \frac{k_{\mathrm{RNAi}}}{\tau} - k_{\mathrm{off}}(\theta) \bar{c} \ . \tag{45}$$

  where k_{RNAi} is a reduction factor of LGN on rate in LGN RNAi cells compared to control cells. Here we have chosen $k_{\mathrm{RNAi}} = 0.1$ (Figures 4N, 4P, S4H).

- In Fig. S4M we consider the effect of introducing an additional bias influencing LGN dynamics along the vertical direction, perpendicular to the direction of initial cell alignment. An external bias is introduced by modifying Eq. 20, taking k_{on} as a function of space:

$$k_{\mathrm{on}}(\theta) = k_{\mathrm{on}}^- + \frac{k_{\mathrm{on}}^+ - k_{\mathrm{on}}^-}{2} (1 - \cos 2\theta) \ . \tag{46}$$

  This function is chosen so that $k_{\mathrm{on}}(\theta)$ take values between k_{on}^- and k_{on}^+, with a minimal value k_{on}^- at $\theta = 0, \pi$ and a maximal value k_{on}^+ at $\theta = \pm\frac{\pi}{2}$. In practice we imposed either $k_{\mathrm{on}}^+ = 2k_{\mathrm{on}}$ and $k_{\mathrm{on}}^- = 0.5k_{\mathrm{on}}$; with k_{on} the reference value used in previous sections (strong bias case in Fig. S4M) or $k_{\mathrm{on}}^+ = 1.2k_{\mathrm{on}}$ and $k_{\mathrm{on}}^- = 0.83k_{\mathrm{on}}$(weak bias case in Fig. S4M).

- In Fig. S4N we consider the effect of varying cell size. To simulate the effect of varying cell size, we scaled the parameters L, L^0_M, L^0_m in Eqs. 37-38 and Table II by a common factor, 1.5 and 2 (Fig. S4N).
- In Fig. S4O we consider the effect of maintaining an elongated cell shape on spindle dynamics. The cell shape is still represented by an ellipse, with constant semi-major and semi-minor axis, set according to Eqs. 37-38 evaluated at $t = 0$. Other parameters are as described above.

[1] Maïwen Caudron, Gertrude Bunt, Philippe Bastiaens, and Eric Karsenti. Spatial coordination of spindle assembly by chromosome-mediated signaling gradients. *Science*, 309(5739):1373–1376, 2005.
