# Supplementary material for: Mechanochemical Crosstalk Produces Cell-Intrinsic Patterning of the Cortex to Orient the Mitotic Spindle
Source: Curr Biol. 2020 Sep 21;30(18):3687–3696.e4. doi: 10.1016/j.cub.2020.06.098 (PMC7521479; doi:10.1016/j.cub.2020.06.098)
Supplement: Document S2. Article plus Supplemental Information [file mmc4.pdf]

Report

Current Biology

Mechanochemical Crosstalk Produces Cell-Intrinsic Patterning of the Cortex to Orient the Mitotic Spindle

Highlights

- Monopolar spindles undergo persistent chase and run movements in flat mitotic cells
- Spindle movements depend on microtubules, LGN, and the Ran-GTP pathway
- A mechanochemical computational model recapitulates monopolar spindle movements
- The model shows how spindles orient along the long cell axis during mitotic rounding

Authors

Andrea Dimitracopoulos, Pragya Srivastava, Agathe Chaigne, ..., Kristian Franze, Guillaume Salbreux, Buzz Baum

Correspondence

ad865@cam.ac.uk (A.D.), guillaume.salbreux@crick.ac.uk (G.S.), b.baum@ucl.ac.uk (B.B.)

In Brief

Dimitracopoulos et al. show how two-way feedback between the cortex and the spindle drives unstable spindle movements in mitotic cells in culture and show how this dynamic system is able to orient the mitotic spindle so that it aligns with the interphase long cell axis, even in the absence of external cues.

Dimitracopoulos et al., 2020, Current Biology *30*, 3687–3696
September 21, 2020 © 2020 The Authors. Published by Elsevier Inc.
https://doi.org/10.1016/j.cub.2020.06.098

CellPress

Current Biology

CellPress
OPEN ACCESS

Report

Mechanochemical Crosstalk Produces Cell-Intrinsic Patterning of the Cortex to Orient the Mitotic Spindle

Andrea Dimitracopoulos,[1,2,*] Pragya Srivastava,[3] Agathe Chaigne,[2] Zaw Win,[2] Roie Shlomovitz,[2,4] Oscar M. Lancaster,[2] Maël Le Berre,[5] Matthieu Piel,[5] Kristian Franze,[1] Guillaume Salbreux,[3,6,7,*] and Buzz Baum[2,6,7,8,*]
[1]Department of Physiology, Development and Neuroscience, University of Cambridge, Downing Street, Cambridge CB2 3DY, UK
[2]MRC Laboratory for Molecular Cell Biology, University College London, Gower Street, London WC1E 6BT, UK
[3]The Francis Crick Institute, 1 Midland Road, London NW1 1AT, UK
[4]Department of Chemical Physics, The Weizmann Institute of Science, PO Box 26, Rehovot 76100, Israel
[5]Institut Curie, PSL Research University, CNRS, UMR 144, Paris 75005, France
[6]Institute for the Physics of Living Systems, University College London, Gower Street, London WC1E 6BT, UK
[7]These authors contributed equally
[8]Lead Contact
*Correspondence: ad865@cam.ac.uk (A.D.), guillaume.salbreux@crick.ac.uk (G.S.), b.baum@ucl.ac.uk (B.B.)
https://doi.org/10.1016/j.cub.2020.06.098

SUMMARY

Proliferating animal cells are able to orient their mitotic spindles along their interphase cell axis, setting up the axis of cell division, despite rounding up as they enter mitosis. This has previously been attributed to molecular memory and, more specifically, to the maintenance of adhesions and retraction fibers in mitosis [1–6], which are thought to act as local cues that pattern cortical Gαi, LGN, and nuclear mitotic apparatus protein (NuMA) [3, 7–18]. This cortical machinery then recruits and activates Dynein motors, which pull on astral microtubules to position the mitotic spindle. Here, we reveal a dynamic two-way crosstalk between the spindle and cortical motor complexes that depends on a Ran-guanosine triphosphate (GTP) signal [12], which is sufficient to drive continuous monopolar spindle motion independently of adhesive cues in flattened human cells in culture. Building on previous work [1, 12, 19–23], we implemented a physical model of the system that recapitulates the observed spindle-cortex interactions. Strikingly, when this model was used to study spindle dynamics in cells entering mitosis, the chromatin-based signal was found to preferentially clear force generators from the short cell axis, so that cortical motors pulling on astral microtubules align bipolar spindles with the interphase long cell axis, without requiring a fixed cue or a physical memory of interphase shape. Thus, our analysis shows that the ability of chromatin to pattern the cortex during the process of mitotic rounding is sufficient to translate interphase shape into a cortical pattern that can be read by the spindle, which then guides the axis of cell division.

RESULTS AND DISCUSSION

Mitosis in animal cells is accompanied by large-scale changes in cell geometry and cytoskeletal organization, making it hard to understand how the spindle in a rounded mitotic cell is able to align along the interphase long cell axis. Thus, to simplify the system, we overexpressed a constitutively activated form of the small guanosine triphosphatase (GTPase) Rap1 to prevent mitotic rounding (Rap1* in this text) [24, 25] in the presence/absence of a kinesin-5 inhibitor S-trityl-L-cysteine (STLC), which both inhibits the formation of a bipolar spindle (Figures 1 and S1A–S1D) [26] and stops cells from exiting mitosis, extending the time window in which to study mitotic cells (Figures 1A–1C; Video S1A) [26]. When cells were treated in this way and imaged by using tubulin-GFP and H2B-mCherry, we observed striking spindle dynamics in flat monopolar cells (Figures S1E–S1I). In 13 out of 15 of Rap1* cells treated with STLC, spindles moved more than 10 μm away from their average position (Figures 1B and S1I), traveling at speeds of up to ~5 μm/min (Figure S1H). As spindles moved across the basal cell cortex, centrosomes led, tilted downward (Figures 1C–1E), while kinetochore-microtubules and DNA followed (Figures 1B and 1D).

We were then able to use this simplified system to test whether cell-extracellular matrix (ECM) adhesions function as positional cues to guide spindle movements, as previously proposed [1]. To do so, we physically confined cells to a height of 5 μm by using polydimethylsiloxane (PDMS) spacers [24] and compared spindle movements in flat cells in the presence (fibronectin [FN]-coated PDMS) (Figure 1F, top) or absence (polyethylene glycol [PEG]-coated PDMS) (Figure 1F, bottom) of adhesive cues. Under these conditions, the distribution of monopolar spindle velocities was unaffected by cell-substrate adhesions (Rap1* + STLC on FN: 0.85 ± 0.60 μm/min; PDMS + STLC on FN: 0.97 ± 0.40 μm/min; PDMS + STLC on PEG: 0.91 ± 0.27 μm/min; mean ± SD; p = 0.37; Kruskal Wallis non-parametric test) (Figures 1F and 1G). Similarly, monopolar spindles moved repeatedly back and forth along the long axis of cells plated on thin micropatterned lines of 10 μm width [24],

This is an open access article under the CC BY license (http://creativecommons.org/licenses/by/4.0/).

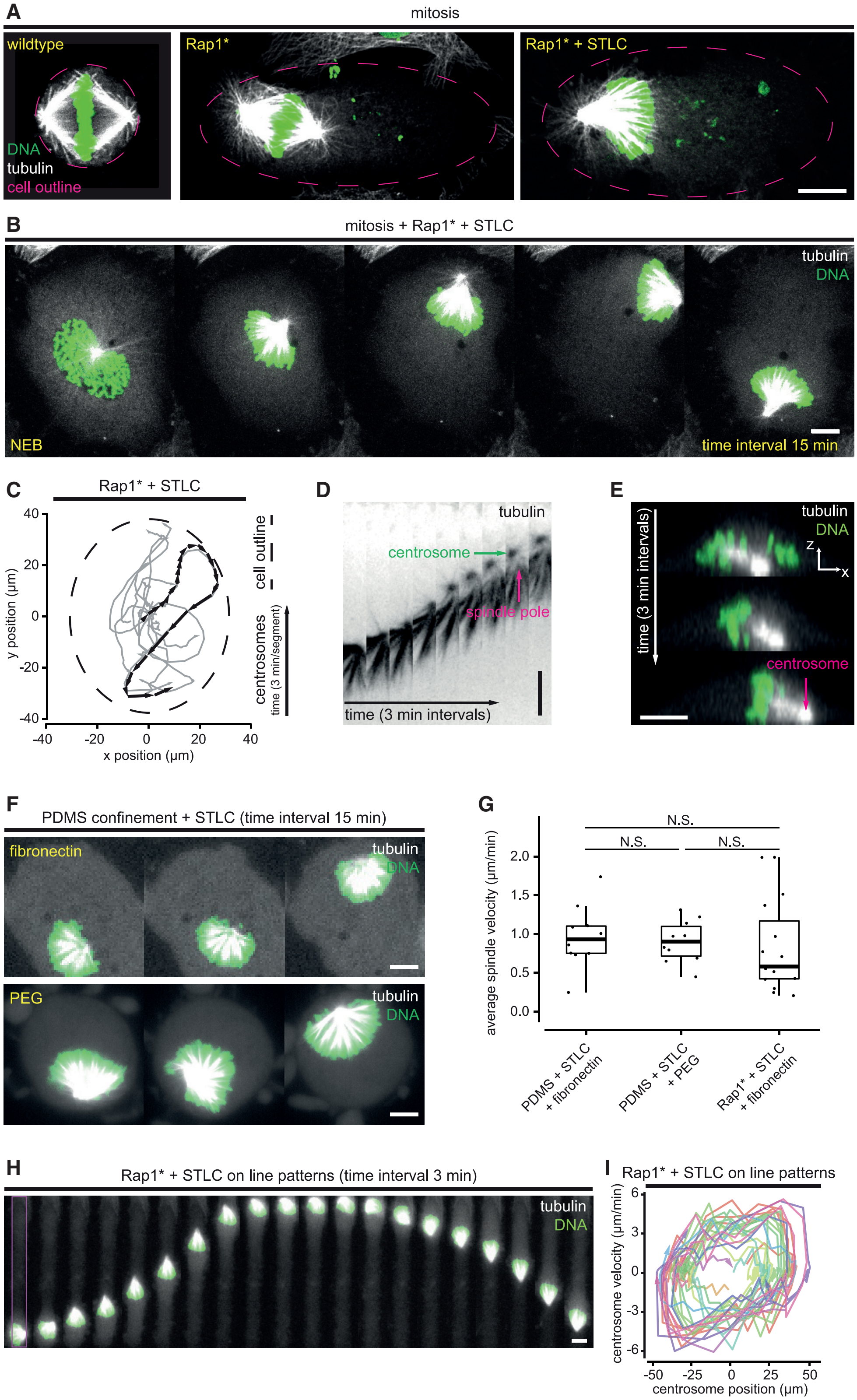

Figure 1. Flat cells with a monopolar spindle as a simplified system to study dynamic spindle positioning

(A) Immuno-fluorescence confocal images of HeLa cells in mitosis on FN-coated unpatterned substrates. Wild-type cells are round and have a bipolar spindle (left). Overexpression of Rap1* results in cells that fail to round up in mitosis (middle), and the combined treatment of Rap1* and STLC results in flat mitotic cells with a monopolar spindle. The dashed line (magenta) shows the elliptical fit of the cell outline.

(legend continued on next page)

apparently blind to the cell poles (Figures 1H and 1I)—where adhesion is strong and retraction fibers are concentrated. Monopolar spindles in these cells reached a dynamic steady state as they oscillated from one cell end to the other, reaching their maximum velocity as they crossed the cell center before pausing and turning (rather than flipping) to repeat the oscillation (Figures 1H and 1I). Taken together, these data suggest that spindle movements in this system are not influenced by local adhesive cues or retraction fibers.

In other systems, spindle orientation depends on Gαi, LGN, and NuMA [8], which recruit Dynein to the cortex, where it exerts forces on astral microtubules to move the spindle. Because all four proteins had a similar cortical distribution at the basal cortex of fixed flat monopolar cells (Figures S1J and S1K), we used GFP-LGN [12] as a proxy for the entire set of proteins in live-imaging experiments. Strikingly, the pattern of GFP-LGN accumulation in these experiments was both dynamic and closely correlated with monopolar spindle movement imaged by using α-tubulin-mCherry (Figures 2A and 2B; Video S1B). In addition, the pattern of GFP-LGN remained stable during periods in which the spindle remained in place (Figures S2D and S2I). In more detail, upon entry into mitosis, the distribution of LGN across the basal surface of flat cells appeared relatively homogeneous. Shortly thereafter, cortical LGN became depleted at the cell center. As LGN polarized, spindles tended to move off center toward regions of the cortex rich in LGN (Figure S2A). LGN was then lost from regions of the cortex that came to within ∼4 μm of the DNA that trailed behind the centrosome and microtubules (Figures 2A, 2B, 2F, top, 2G, left graph, S2B, and S2C), in line with the idea of a Ran-GTP-dependent inhibitory signal emanating from mitotic chromatin [12, 27]. At the same time, LGN was observed re-accumulating at regions of the cortex that were previously depleted of LGN (Figures 2A, 2B, S2D, and S2E). This led to a cycle of LGN loss from the cortex close to the chromatin and its re-accumulation at sites far from the moving spindle.

To determine whether these correlations between spindle movement and cortical LGN patterning reflect a causal relationship between the two systems, as expected based on previous work, we first treated cells with low doses of the microtubule depolymerizing drug nocodazole to assess whether monopolar spindle movements depend on astral microtubules, whose distribution we determined (Figures 2C, 2D, and S2G) [28, 29]. This proved to be the case: monopolar spindle movements were markedly slower in nocodazole-treated cells than in the control (Figure 2E, left) ($p < 0.001$; Mann-Whitney *U* test), leading to a pause in LGN dynamics (Figure S2I). Second, when we used RNAi to silence LGN expression, spindle movements were dramatically reduced as expected if LGN is required for force generation at the cortex (Figure 2E, right) ($p < 0.001$; Mann-Whitney *U* test). Third, to determine whether chromatin-based signals are responsible for the dynamic changes in the association of LGN with the cortex [12], we treated flat monopolar cells with importazole for short periods (Figure 2F) to interfere with chromatin-based Ran-GTP signaling [12, 27, 30]. Importazole reduced the clearance of LGN from the cortex close to chromatin, leading to a reduction in the LGN inhibition range (Figures 2F, 2G, and S2H) (4.5 μm control, 1.2 μm importazole-treated; $p < 0.01$; Mann-Whitney *U* test; see Methods S1), as expected based on previous work [27]. Additionally, in a rare flat, untreated cell in which a bipolar spindle broke into two, LGN was seen locally clearing from the basal membrane in the vicinity of both half-spindles (Figure S2F; Video S1C), implying that the effect is mediated by local short-range signaling. Together, these data support the idea that LGN and other cortical proteins controlling Dynein-mediated forces on astral microtubules, together with the Ran-GTP gradient centered on mitotic chromatin, constitute a dynamic feedback system that links the spindle and the cortex. This feedback prevents the system from reaching a static equilibrium state, giving rise to the striking instability of monopolar spindle positioning in flat cells.

Despite previous work suggesting a role for actin cortical mechanics in spindle orientation [2], we found no correlation between the organization of the actin cortex and the position or movement of the spindle (Figure S2J). In line with this, two perturbations that inhibited cortical myosin did not alter spindle movement ($p > 0.05$; Mann-Whitney *U* test) (Figures S2L and S2M). Nevertheless, when we disrupted the actin cortex by using high doses of latrunculin B, LGN (and associated membrane) was pulled toward the centrosome in a microtubule-dependent manner (Figure S2K). As previously suggested by work in *C. elegans* embryos and HeLa cells [31, 32], this implies that the actin cortex is not required for cortical motors to exert forces on the spindle. Instead, the cortex provides a stable platform that resists cortical deformation as the spindle moves.

(B) Time-lapse confocal images of a HeLa cell (Rap1* + STLC) on a uniformly FN-coated substrate as it enters mitosis: at NEB, the two centrosomes fail to separate, resulting in monopolar spindle formation. The monopolar spindle moves freely and continuously.

(C) Plot of the trajectories of monopolar spindles in mitotic HeLa cells on fibronectin-coated adhesive substrates (Rap1* + STLC; n = 15). The trajectory of the spindle shown in (B) is highlighted in black, and the outline of the cell is shown as a dashed line, although other trajectories are shown in gray.

(D) Detailed time lapse of the monopolar spindle shown in (B). The centrosome leads the movement, and the rest of the spindle follows.

(E) X-Z section of a confocal time lapse of a representative HeLa cell treated with Rap1* + STLC on a FN-coated unpatterned substrate. Centrosomes lie close to the basal membrane as the spindle moves.

(F) Spindle motion still occurs in STLC-treated HeLa cells on FN-coated surfaces under a FN-coated PDMS roof (upper graphic, top row; n = 10), as well as on non-adherent PEG-coated surfaces, held flat under a PEG-coated roof of PDMS (bottom; n = 10).

(G) Boxplots of STLC-treated monopolar spindle velocities in cells flattened by different means. Physical confinement with FN or PEG coating (shown in F) or genetic treatments (Rap1* overexpression; shown in B and C) results in similar monopolar spindle behavior (PDMS + STLC + FN n = 10; PDMS + STLC + PEG n = 10; Rap1* + STLC + FN n = 15; significance tests: $p > 0.05$; Mann-Whitney U test). Thick bars and boxes indicate median values and lower/upper quartiles, respectively. Whiskers extend to the smallest/largest value, but no further than 1.5 times the interquartile range.

(H) Wide-field time-lapse images of a mitotic HeLa cell (Rap1* + STLC) on a FN-coated line pattern (10 μm width, magenta box). The spindle follows a 1D path, alternating its direction of movement.

(I) Phase portrait of centrosome motion in monopolar cells on line-patterns (as in H). The phase portrait reveals that the spindle motion alternates between fast motion and pausing near cell ends as it changes direction.

All cells in time-lapse images are expressing tubulin-GFP and H2B-mCherry. All scale bars indicate 10 μm. See also Figure S1 and Video S1A.

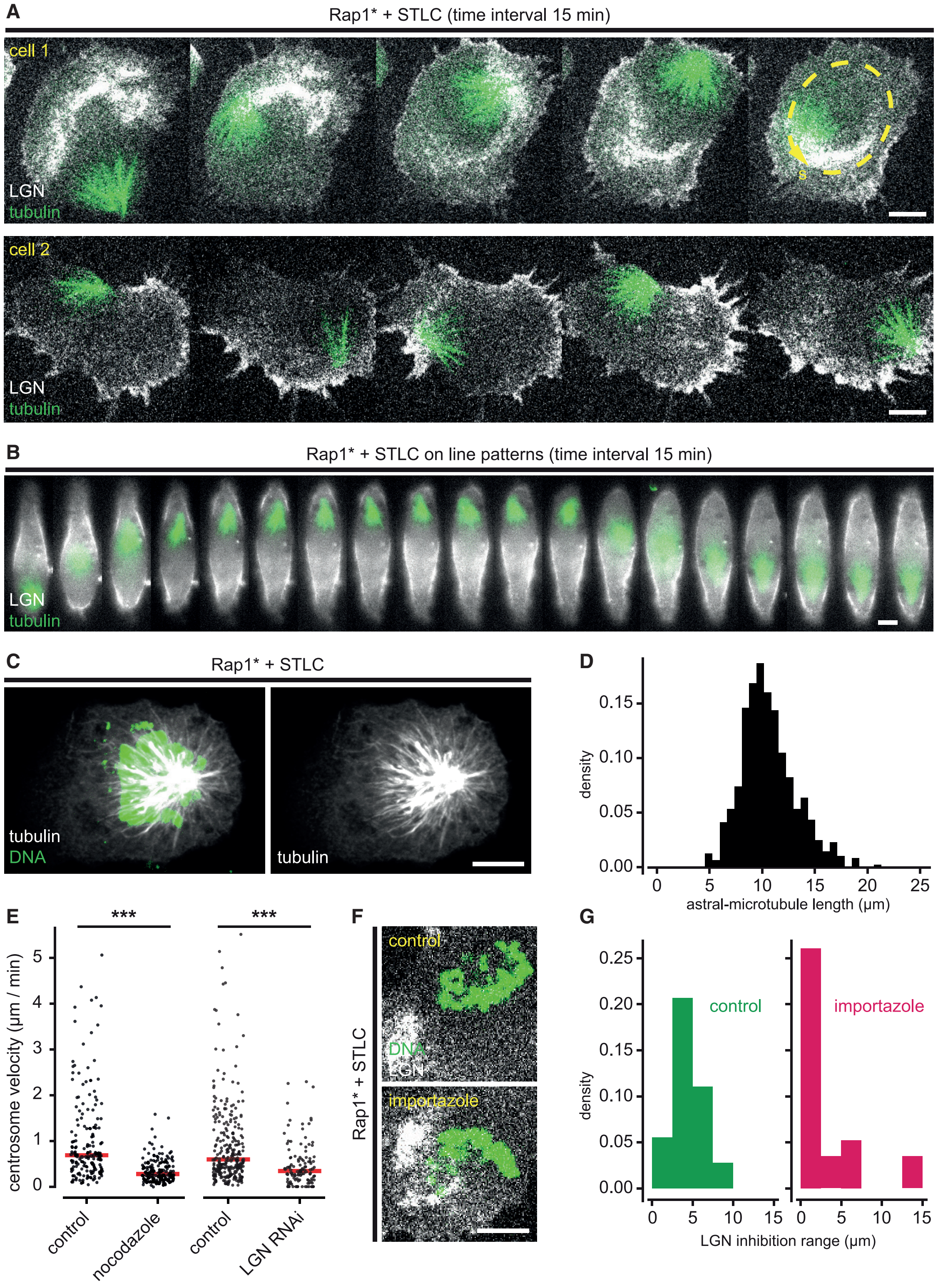

Figure 2. LGN, astral microtubules, and the ran pathway are responsible for monopolar spindle motion in flat cells

(A and B) Time-lapse imaging reveals a feedback loop between spindle position and cortical LGN levels. LGN levels decrease in regions close to the spindle and increase far from the spindle.

(A) Time-lapse images of mitotic HeLa cells (Rap1* + STLC) on FN-coated unpatterned substrates (confocal imaging; two-representative example). The last frame of the top montage shows the reference line used to obtain the kymograph in Figure 3B.

(legend continued on next page)

CellPress
OPEN ACCESS

To better understand how such dynamical feedback between the cortex and the spindle is likely to work, we developed a computational model of monopolar spindle movement in flat cells. This model includes (1) DNA-dependent inhibition of cortical LGN and (2) cortical dynein motors that pull on astral microtubules to exert forces on the spindle (Figures 3A and S3A). In the model, cortical LGN diffuses on the cell surface and undergoes exchange with cytoplasmic LGN with on and off rates denoted by$k_{on/off}$, under the assumption that the cytoplasmic pool of LGN acts as a large reservoir. Cortical LGN is affected by spindle movement because dissociation of LGN from the cortex (k_{off} rate) occurs preferentially near the DNA, as observed in experiments (Figures 2A, 2B, 2F, and 2G; Videos S1B and S1C). To test whether this simple model can account for the observed dynamics of LGN, we quantified the position of the DNA, centrosomes, and LGN profiles in flat cells along the path of monopolar spindle movement (Figure 2A, top, last frame), which we visualized in kymographs as a 1D, periodic motion (Figures 3B and S3H). Taking the motion of DNA as an input, we then obtained theoretical cortical LGN profiles (Figures 3B and S3H) by using a small value for the diffusion constant ($D = 0.01\ \mu m^2/min$), consistent with the experimentally observed stability of cortical accumulations of LGN far from the spindle (Figures S2D and S2E). With unbinding occurring near the DNA with a characteristic timescale of ~10 min and far from the DNA with a timescale of ~90 min, kymographs generated from simulations reproduced key features of the experimental data (Figures 3B and S3H; Methods S1). We then assumed monopolar spindle movement to be driven by forces acting on the end of astral microtubules, taken as proportional to the sampled cortical LGN concentration at microtubule plus ends. Using our experimental measure of the astral microtubule length distribution (Figures 2C, 2D, 3C, S2G, and S3B), this simple model was also able to account for the experimentally observed motion of the DNA in the 12 cells we studied in detail (Figures 3D, S3I, and S3J), by adjusting a free parameter $v_0 = 6.7 \pm 6.7 \mu m/min$. This characteristic velocity depends on the force exerted by dynein motors at a reference LGN concentration, the number of microtubules, and the friction coefficient acting on the spindle (Methods S1).

Using the parameters defined above to generate a 1D dynamic model of a monopolar spindle, we found that, starting with a uniform concentration of LGN (Figures 3E and S3C), the spindle eventually moves with a constant velocity, following a traveling wave of LGN (Figures 3E, 3F, S3C, and S3D). This closely resembles monopolar spindle movement in real cells (Figures 3B, 3D, and S3H–S3J) and suggests that there is no stable rest state for a monopolar spindle configuration in which the centrosome and DNA are physically separate (Figure S3E; Methods S1). Interestingly, the model predicts that, even if the centrosome and DNA occupy the same position (e.g., equivalent to the centrosome lying on top of the DNA in 3D), the system can still undergo spontaneous symmetry breaking, leading to monopolar spindle movement (Figures S3C and S3F; Methods S1), as observed in flat monopolar cells entering mitosis (Figure S2A). Simulations also replicated the back-and-forth oscillations seen in cells plated on thin micropatterned lines (Figures S3K–S3N, 1H, and 1I), although this 1D model cannot capture spindle turns.

By modifying the model to study the movement of bipolar spindles in flat cells, we found that, in addition to a non-moving solution arising from the inherent symmetry of the bipolar configuration, the coupled spindle and LGN dynamics can also give rise to bipolar spindle movement (Figure S3G), depending on parameters (Figures S3U–S3X). This fits with our previous work, in which we showed that spindles move off center in cells that are prevented from rounding [24, 33], and with bipolar spindle behavior in Rap1* cells confined to 1D line micropatterns (Figures S3O–S3T and S3Y).

Finally, we wondered whether the mechanochemical model we identified in flat cells could shed light on the functional consequences of dynamic crosstalk between the spindle and cortex in the context of a normal mitosis, where cells assemble and orient a bipolar spindle as they round. To do so, we generated a 2D model on the basis of the parameters defined above (Figures S4A and S4B; Methods S1), in which we included the dynamics of cell rounding (Figures 4A and S4C; Video S1D) [34] and bipolar spindle assembly (completed ~9 min after nuclear envelope breakdown [NEB] [24]) (Figures S4D and S4E). For simplicity, using these values, we modeled mitosis as two distinct phases (Figure 4B). In phase I, as cells round, DNA-mediated inhibition patterns cortical LGN (Figures 4B and S4C). In phase II, from 9 min after NEB onward, the bipolar spindle interacts with cortical motors through astral-microtubule-mediated forces, in an LGN-dependent manner (Figures 4B and 4G). During this second phase, we assume that the spindle rotates according to the torque arising from cortical forces and acting on astral microtubules, in line with previous models of spindle orientation [1, 20–22]. We then compared simulation results to experiments in which we observed cells expressing GFP-LGN as they rounded (Figure 4A). In both experiments and in the model, LGN

(B) Time-lapse images of a mitotic HeLa cell (Rap1* + STLC) on a FN-coated line pattern (wide-field imaging, representative example).

(C) Maximum projection of immuno-fluorescence confocal images of a HeLa cell (Rap1* + STLC) in mitosis on a FN-coated unpatterned substrate. Microtubules emanate radially from the spindle pole.

(D) Histogram of astral microtubule lengths, measured as the distance between the spindle pole and the microtubule ends in HeLa cells (as shown in C; n = 8).

(E) Perturbing astral microtubules or LGN reduces spindle velocity. Plots show centrosome velocities in control HeLa cells (Rap1* + STLC), in cells treated with small doses of nocodazole to perturb astral microtubules (control n = 9; nocodazole n = 9), and in LGN RNAi cells (control n = 14; LGN RNAi n = 6; significance tests: $p < 0.001$; Mann-Whitney U test). Red lines indicate median values

(F and G) Perturbation of the Ran pathway reduces LGN clearance close to the DNA.

(F) Confocal images of mitotic HeLa cells (Rap1* + STLC) on FN-coated unpatterned substrates, treated with importazole to perturb the Ran pathway. Compared with control, importazole-treated cells show higher levels of LGN near the DNA.

(G) Histograms show minimum distance between DNA and high-level LGN (thresholded at the 0.975 quantile) in control (n = 29) and importazole-treated (n = 23) HeLa cells (Rap1* + STLC). In importazole-treated cells, the LGN inhibition range is shorter than in control cells (medians: 4.5 μm control; 1.2 μm importazole treated; $p < 0.01$; Mann-Whitney *U* test).

Images of live cells show GFP-LGN, tubulin-mCherry, and/or H2B-mCherry. All scale bars indicate 10 μm. See also Figure S2 and Videos S1B and S1C.

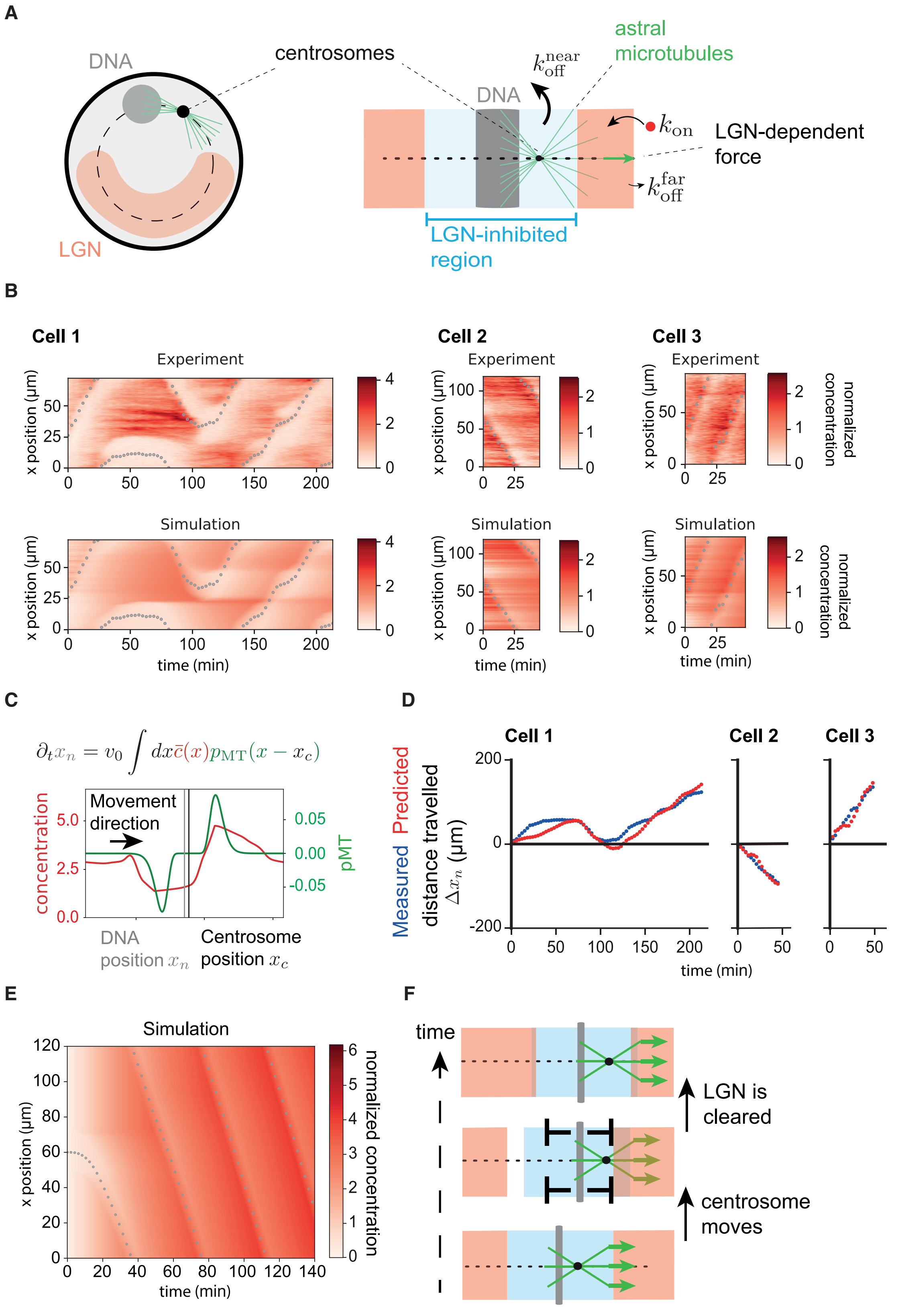

(legend on next page)

crescents formed at the poles along the long cell axis in the first ~9 min after NEB (experiment: Figures 4A, 4D, 4E, and S4I–S4K; model: Figures 4F, 4I, S4J, and S4L). In the model, this can be understood as a simple consequence of LGN accumulation due to cell rounding and DNA-mediated inhibition of LGN along the short cell axis, as previously observed [12, 27]. Moreover, this polarized distribution of LGN was retained for an extended period of time after spindle reorientation in both simulations and experiments (Figures 4A, 4D–4F, 4I, and S4I). Then, in phase II, astral-microtubule-mediated forces act on the bipolar spindle to change its alignment to the interphase long cell axis (Figures 4G and 4H). As it does so, the polarized distribution of LGN (established in phase I) leads to an imbalance in the cortical forces acting on astral microtubules, leading to spindle rotation, even though cells are round, i.e., in the absence of a geometrical cue (Figure 4I). In fact, this simple 2D model of cell-intrinsic signaling recapitulates the reorientation of mitotic spindles toward the interphase long cell axis (Figure 4I) in rounded mitotic cells, across a wide range of initial angles (Figures 4J, 4K, and S4G), yielding spindle reorientation dynamics that are similar to those observed in HeLa cells rounding up and dividing on FN-coated micropatterned lines (Figures 4L and 4M), where there is a strong interphase shape signal (Figure S4C). Further, as expected, spindle alignment was compromised in experiments where LGN was depleted through RNAi and in equivalent simulations where LGN levels were strongly reduced (Figures 4N–4Q, S4F, and S4H; Videos S1E and S1F).

Together, these data indicate that the dynamic DNA/LGN mechanochemical system, which we defined on the basis of monopolar spindle behavior in flat cells (Figures 1, 2, and 3), is sufficient to enable cells undergoing mitotic rounding to translate interphase cell shape into a cortical pattern of LGN, which persists after cells become completely rounded (Figure 4). Although spindle movement in this system does not depend on cell-substrate mitotic adhesion sites (Figures 1F–1I) and is unaffected by perturbations that affect cortical mechanics (Figures S2L and S2M), local differences in cortical stiffness will likely impact spindle movements and alignment [2, 32]. Furthermore, these observations do not mean that external cues cannot influence spindle orientation in this context. When a strong external cue perpendicular to the interphase cell axis was introduced into the model, the spindle rotated toward the external cue and maintained a stable orientation perpendicular to the interphase cell axis (Figure S4M). This shows that, although this intrinsic patterning system is sufficient to position the spindle, it can be easily over-ridden. In these more complex scenarios, the influence of the intrinsic patterning based on spindle-cortical crosstalk functions will depend on other factors, such as the strength of extrinsic cues, gradients in cortical stiffness, the relative size of the cell and spindle, and the persistence of cell elongation (Figures S4M–S4Q), in ways that will be interesting to explore in the future. It was by studying the simplest case, though, that we were able to uncover rules of dynamic spindle-cortical crosstalk, which, as we show, are sufficient to explain how spindles orient in relation to the interphase long cell axis as cells round in the absence of any extrinsic signals.

STAR★METHODS

Detailed methods are provided in the online version of this paper and include the following:

- KEY RESOURCES TABLE
- RESOURCE AVAILABILITY
  - Lead Contact
  - Materials Availability
  - Data and Code Availability
- EXPERIMENTAL MODEL AND SUBJECT DETAILS
- METHOD DETAILS
  - DNA and siRNA transfection
  - Drug treatments
  - Micropatterning and Cell Confinement
  - Live-cell microscopy

Figure 3. A mechanochemical model describing the LGN/DNA/microtubule interaction accounts for monopolar spindle motion

(A) Schematic of 1D model simplification. Experimental data show DNA, centrosome positions, and LGN profiles projected on a circular line. These data are compared to a 1D model of monopolar spindle formation with periodic boundary conditions. In the model, LGN unbinding is faster near the DNA (blue region, rate k_{off}^{near}) than away from the DNA (red region, rate k_{off}^{far}). The centrosome/DNA/spindle structure is subjected to cortical forces acting on astral microtubules. These cortical forces increase with cortical LGN concentration.

(B) Simulations of LGN inhibition by spindle motion recapitulate LGN experimental profiles. Top panels: experimental kymographs show the cortical LGN fluorescence intensity over space and time (red colors) and the DNA position over time (gray dots) for 3 representative cells. Bottom panels: corresponding kymographs of simulated cortical LGN concentration are shown, imposing the experimentally measured DNA position. In the simulation, the cortical LGN is modeled using simple binding/unbinding rates that depend on distance to the DNA. See Figure S3H for kymographs of 9 additional analyzed cells. Concentrations have been normalized to the spatiotemporal mean in kymographs.

(C) DNA velocity is assumed to be set by the force exerted on the DNA-spindle-centrosome structure by cortical motors pulling on astral microtubules. Top: equation of motion for the DNA is shown, capturing the dependency of the force acting on the DNA-spindle-centrosome structure on the normalized LGN concentration $\bar{c}$ and the signed distribution of microtubule ends, p_{MT}. x_n and x_c are the positions of the DNA center and of the centrosome. Bottom: example plot of normalized LGN concentration (red) and the distribution of microtubules (green) in the model are shown.

(D) Comparison to experimental data of DNA motion predicted in (C). Graphs show the distance traveled by the DNA Δx_n as a function of time, experimentally measured (blue) and predicted from the equation given in (C) (red) for 3 representative cells. The value of the proportionality coefficient v_0 is chosen independently for each cell. See Figure S3I for graphs of 9 additional analyzed cells.

(E) LGN concentration and spindle motion in full mechanochemical feedback model (colors are as in B). The system settles in a steady state characterized by constant DNA velocity and a traveling wave of LGN concentration. Parameters are given in Methods S1.

(F) Schematic of mechanism of steady-state motion. The depletion of LGN around DNA results in an asymmetric distribution of LGN around the centrosome and a net force acting on the spindle. As a result of this force, the DNA-centrosome-spindle structure moves. Further LGN depletion around the DNA results in a traveling wave of LGN concentration.

See also Figure S3.

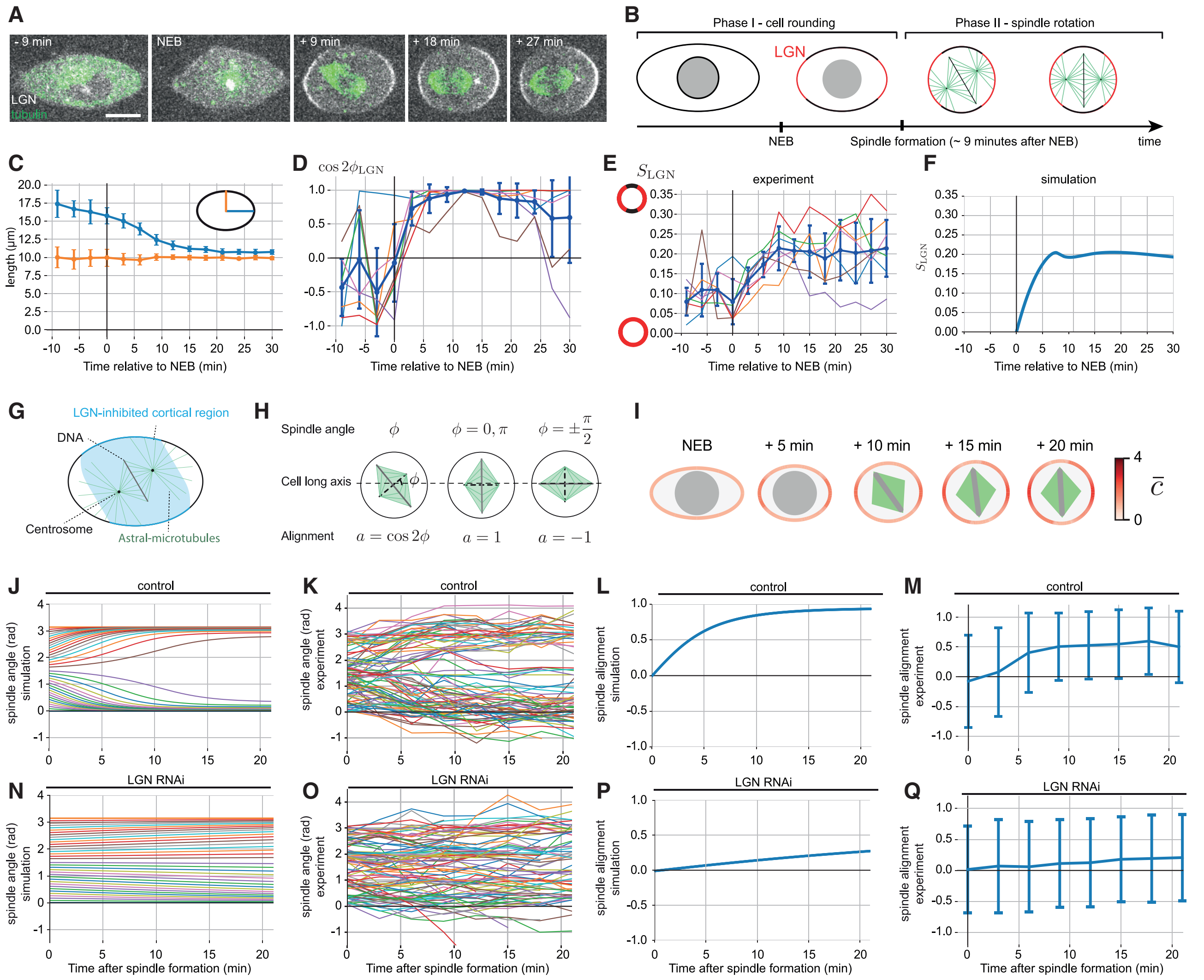

Figure 4. Mechanochemical model is sufficient to explain how bipolar spindles align with the interphase long cell axis as cells round

(A) Experimental images showing bipolar spindle formation and reorientation upon entry into mitosis. Confocal images of a representative HeLa cell expressing GFP-LGN on a FN-coated unpatterned substrate show cell shape changes around NEB and bipolar spindle formation and reorientation after NEB. LGN is inhibited along the short axis of the cell and can freely accumulate at the two crescents corresponding to the interphase long axis of the rounded mitotic cell. The scale bar indicates 10 µm.

(B) Schematic of the main phases of bipolar spindle orientation. In phase 1, the cell undergoes mitotic rounding and LGN accumulates in the cortex after NEB as cells assemble a bipolar spindle. In phase 2, the bipolar spindle reorients in the rounded cell.

(C) Quantification of cell shape changes during mitotic rounding for mitotic HeLa cells (n = 7), for which (A) is a representative example. Blue, long cell axis half-length; orange, short cell axis half-length. Error bars, standard deviation.

(D) Alignment of the nematic angle calculated from LGN fluorescence intensity profiles with the long cell axis for mitotic HeLa cells (n = 7), for which (A) is a representative example. Thick blue line, dots, and error bars indicate the average and standard deviation of alignment for different quantified cells. Other thin lines show the alignment for individual cells. A strong alignment with the long cell axis (average alignment close to 1) is reached ~10 min after NEB.

(E) Magnitude of nematic order parameter S_{LGN} for LGN concentration as a function of time (see Methods S1 for a definition) for mitotic HeLa cells (n = 7), for which (A) is a representative example. Thick blue line, dots and error bars indicate the average and standard deviation of alignment for different quantified cells. Other thin lines correspond to S_{LGN} for individual cells. The nematic order parameter increases between 0 and 10 min after NEB and stabilizes after 10 min.

(F) Magnitude of nematic order parameter S_{LGN} as a function of time, for the simulation shown in (I). The initial rise after NEB of LGN ordering in the cell contour is comparable to that measured in experiments in (E).

(G) Schematic of mechanochemical model for bipolar spindle orientation. In the model, loss of LGN from the cortex is induced within a range of 4 µm away from the DNA. LGN influences the cortical forces acting on the astral microtubules from the cortex, resulting in a torque acting on the spindle and driving its reorientation after spindle formation. At the same time, the cell undergoes mitotic cell rounding.

(H) An alignment parameter $a = \cos 2\phi$ can be defined for ϕ the spindle angle in relation to the interphase long cell axis, quantifying how good ($a \rightarrow 1$) or bad ($a \rightarrow -1$) spindle alignment is with respect to the long axis.

(I) Images depict outputs from the spindle orientation simulation in a cell undergoing mitotic rounding, at different times after NEB. The initial angle of the spindle in relation to the long axis is $\phi = \pi/4$. The red color on the cell contour corresponds to the concentration of LGN: DNA is represented by a gray circle up to 9 min after

(legend continued on next page)

- ○ Immunofluorescence and antibodies
- QUANTIFICATION AND STATISTICAL ANALYSIS
  - ○ Quantification
  - ○ Statistical analysis and visualization

SUPPLEMENTAL INFORMATION

Supplemental Information can be found online at https://doi.org/10.1016/j.cub.2020.06.098.

ACKNOWLEDGMENTS

We thank Susannah McLaren, Clotilde Cadart, Thomas Surrey, Margarida Dantas, and members of the Baum and Franze groups for critical readings and feedback of the manuscript. We also thank Andrew Vaughan for microscopy support and Iain Cheeseman for providing cell lines. B.B. was supported by two CRUK Programme grants (C1529/A9786 and C1529/A17343) and received additional support from the MRC-LMCB (MC_CF12266). G.S. and P.S. were supported by the Francis Crick Institute, which receives its core funding from Cancer Research UK (FC001317), the UK Medical Research Council (FC001317), and the Wellcome Trust (FC001317), and A.D. was supported by UCL's EPSRC-sponsored CoMPLEX PhD programme and the Herchel-Smith Fund.

AUTHOR CONTRIBUTIONS

B.B. and A.D. conceived the project, with input from R.S. and O.M.L. The project was developed jointly by A.D., B.B., and G.S. Experiments were carried out by A.D., with supervision from B.B. and input from M.P., M.L.B., and K.F. Spindle orientation studies were carried out by A.D. and A.C. The model was conceived by B.B., A.D., P.S., and G.S. The modeling was carried out by P.S. and G.S. The paper was written by A.D., B.B., and G.S. All authors reviewed and edited the manuscript.

DECLARATION OF INTERESTS

The authors declare no competing interests.

Received: December 19, 2019
Revised: May 14, 2020
Accepted: June 29, 2020
Published: July 30, 2020

REFERENCES

1. Théry, M., Jiménez-Dalmaroni, A., Racine, V., Bornens, M., and Jülicher, F. (2007). Experimental and theoretical study of mitotic spindle orientation. Nature *447*, 493–496.
2. Fink, J., Carpi, N., Betz, T., Bétard, A., Chebah, M., Azioune, A., Bornens, M., Sykes, C., Fetler, L., Cuvelier, D., and Piel, M. (2011). External forces control mitotic spindle positioning. Nat. Cell Biol. *13*, 771–778.
3. Kotak, S., and Gönczy, P. (2013). Mechanisms of spindle positioning: cortical force generators in the limelight. Curr. Opin. Cell Biol. *25*, 741–748.
4. Dix, C.L., Matthews, H.K., Uroz, M., McLaren, S., Wolf, L., Heatley, N., Win, Z., Almada, P., Henriques, R., Boutros, M., et al. (2018). The role of mitotic cell-substrate adhesion re-modeling in animal cell division. Dev. Cell *45*, 132–145.e3.
5. Lock, J.G., Jones, M.C., Askari, J.A., Gong, X., Oddone, A., Olofsson, H., Göransson, S., Lakadamyali, M., Humphries, M.J., and Strömblad, S. (2018). Reticular adhesions are a distinct class of cell-matrix adhesions that mediate attachment during mitosis. Nat. Cell Biol. *20*, 1290–1302.
6. Théry, M., Racine, V., Pépin, A., Piel, M., Chen, Y., Sibarita, J.-B., and Bornens, M. (2005). The extracellular matrix guides the orientation of the cell division axis. Nat. Cell Biol. *7*, 947–953.
7. Colombo, K., Grill, S.W., Kimple, R.J., Willard, F.S., Siderovski, D.P., and Gönczy, P. (2003). Translation of polarity cues into asymmetric spindle positioning in Caenorhabditis elegans embryos. Science *300*, 1957–1961.
8. Du, Q., and Macara, I.G. (2004). Mammalian pins is a conformational switch that links NuMA to heterotrimeric G proteins. Cell *119*, 503–516.
9. Du, Q., Stukenberg, P.T., and Macara, I.G. (2001). A mammalian partner of inscuteable binds NuMA and regulates mitotic spindle organization. Nat. Cell Biol. *3*, 1069–1075.
10. Gotta, M., and Ahringer, J. (2001). Distinct roles for Galpha and Gbetagamma in regulating spindle position and orientation in Caenorhabditis elegans embryos. Nat. Cell Biol. *3*, 297–300.
11. Gotta, M., Dong, Y., Peterson, Y.K., Lanier, S.M., and Ahringer, J. (2003). Asymmetrically distributed C. elegans homologs of AGS3/PINS control spindle position in the early embryo. Curr. Biol. *13*, 1029–1037.
12. Kiyomitsu, T., and Cheeseman, I.M. (2012). Chromosome- and spindle-pole-derived signals generate an intrinsic code for spindle position and orientation. Nat. Cell Biol. *14*, 311–317.
13. Kotak, S., Busso, C., and Gönczy, P. (2012). Cortical dynein is critical for proper spindle positioning in human cells. J. Cell Biol. *199*, 97–110.
14. Lorson, M.A., Horvitz, H.R., and van den Heuvel, S. (2000). LIN-5 is a novel component of the spindle apparatus required for chromosome segregation and cleavage plane specification in Caenorhabditis elegans. J. Cell Biol. *148*, 73–86.
15. Merdes, A., Ramyar, K., Vechio, J.D., and Cleveland, D.W. (1996). A complex of NuMA and cytoplasmic dynein is essential for mitotic spindle assembly. Cell *87*, 447–458.
16. Srinivasan, D.G., Fisk, R.M., Xu, H., and van den Heuvel, S. (2003). A complex of LIN-5 and GPR proteins regulates G protein signaling and spindle function in C elegans. Genes Dev. *17*, 1225–1239.
17. Woodard, G.E., Huang, N.N., Cho, H., Miki, T., Tall, G.G., and Kehrl, J.H. (2010). Ric-8A and Gi alpha recruit LGN, NuMA, and dynein to the cell cortex to help orient the mitotic spindle. Mol. Cell. Biol. *30*, 3519–3530.
18. Bosveld, F., Markova, O., Guirao, B., Martin, C., Wang, Z., Pierre, A., Balakireva, M., Gaugue, I., Ainslie, A., Christophorou, N., et al. (2016). Epithelial tricellular junctions act as interphase cell shape sensors to orient mitosis. Nature *530*, 495–498.

NEB and by a gray line after 9 min after NEB, to represent the metaphase plate. For simplicity, astral microtubules are not depicted, and spindle microtubules are shown as two triangles to visualize the spindle orientation. In the simulation, LGN accumulation due to cell rounding and feedback from DNA inhibition leads to spindle rotation along the long cell axis.
(J, L, N, and P) Predicted spindle dynamics as cell round, with normal or reduced levels of LGN, as a function of time. Spindle dynamics were obtained from simulations as shown in (I).
(J and N) Simulated trajectories of spindle angles in relation to long axis as a function of time, in control conditions (J) and for reduced LGN concentrations (N).
(L and P) Average alignment of the spindle angle with respect to the long axis, as a function of time, obtained from simulations in (J) and (N), respectively. The alignment is defined in (H). Simulation trajectories are obtained by choosing a set of uniformly spaced initial spindle angles. For each simulation, spindle rotation is determined by the torque exerted by LGN-dependent forces on astral microtubules, in a cell which changes its shape. See Methods S1 for details.
(K, M, O, and Q) Plots of spindle orientation trajectories in control (n = 94) and LGN RNAi (n = 96) cells on micropatterned lines fit with predicted behavior. Experimental trajectories of spindle angles in relation to the long cell axis in control conditions (K) and LGN-RNAi (O) (1 of 96 trajectories is out of range) are shown.
(M and Q) Average alignment of the spindle angle with respect to the long axis as a function of time, obtained from experiments in (K) and (O), respectively.
See also Figure S4 and Videos S1D–S1F.

19. Pecreaux, J., Röper, J.-C., Kruse, K., Jülicher, F., Hyman, A.A., Grill, S.W., and Howard, J. (2006). Spindle oscillations during asymmetric cell division require a threshold number of active cortical force generators. Curr. Biol. *16*, 2111–2122.

20. Corrigan, A.M., Shrestha, R., Draviam, V.M., and Donald, A.M. (2015). Modeling of noisy spindle dynamics reveals separable contributions to achieving correct orientation. Biophys. J. *109*, 1398–1409.

21. Bjerknes, M. (1986). Physical theory of the orientation of astral mitotic spindles. Science *234*, 1413–1416.

22. Minc, N., Burgess, D., and Chang, F. (2011). Influence of cell geometry on division-plane positioning. Cell *144*, 414–426.

23. Grill, S.W., Kruse, K., and Jülicher, F. (2005). Theory of mitotic spindle oscillations. Phys. Rev. Lett. *94*, 108104.

24. Lancaster, O.M., Le Berre, M., Dimitracopoulos, A., Bonazzi, D., Zlotek-Zlotkiewicz, E., Picone, R., Duke, T., Piel, M., and Baum, B. (2013). Mitotic rounding alters cell geometry to ensure efficient bipolar spindle formation. Dev. Cell *25*, 270–283.

25. Dao, V.T., Dupuy, A.G., Gavet, O., Caron, E., and de Gunzburg, J. (2009). Dynamic changes in Rap1 activity are required for cell retraction and spreading during mitosis. J. Cell Sci. *122*, 2996–3004.

26. DeBonis, S., Skoufias, D.A., Lebeau, L., Lopez, R., Robin, G., Margolis, R.L., Wade, R.H., and Kozielski, F. (2004). In vitro screening for inhibitors of the human mitotic kinesin Eg5 with antimitotic and antitumor activities. Mol. Cancer Ther. *3*, 1079–1090.

27. Bird, S.L., Heald, R., and Weis, K. (2013). RanGTP and CLASP1 cooperate to position the mitotic spindle. Mol. Biol. Cell *24*, 2506–2514.

28. di Pietro, F., Echard, A., and Morin, X. (2016). Regulation of mitotic spindle orientation: an integrated view. EMBO Rep. *17*, 1106–1130.

29. Machicoane, M., de Frutos, C.A., Fink, J., Rocancourt, M., Lombardi, Y., Garel, S., Piel, M., and Echard, A. (2014). SLK-dependent activation of ERMs controls LGN-NuMA localization and spindle orientation. J. Cell Biol. *205*, 791–799.

30. Tsuchiya, K., Hayashi, H., Nishina, M., Okumura, M., Kanemaki, M.T., Goshima, G., and Kiyomitsu, T. (2019). Acute degradation reveals that Ran-Importin network dynamically polarizes and maintains HURP, but not NuMA, on human mitotic spindle. bioRxiv. https://doi.org/10.1101/473538.

31. Redemann, S., Pecreaux, J., Goehring, N.W., Khairy, K., Stelzer, E.H.K., Hyman, A.A., and Howard, J. (2010). Membrane invaginations reveal cortical sites that pull on mitotic spindles in one-cell C. elegans embryos. PLoS ONE *5*, e12301.

32. Zheng, Z., Wan, Q., Liu, J., Zhu, H., Chu, X., and Du, Q. (2013). Evidence for dynein and astral microtubule-mediated cortical release and transport of Gαi/LGN/NuMA complex in mitotic cells. Mol. Biol. Cell *24*, 901–913.

33. Cadart, C., Monnier, S., Grilli, J., Sáez, P.J., Srivastava, N., Attia, R., Terriac, E., Baum, B., Cosentino-Lagomarsino, M., and Piel, M. (2018). Size control in mammalian cells involves modulation of both growth rate and cell cycle duration. Nat. Commun. *9*, 3275.

34. Matthews, H.K., Delabre, U., Rohn, J.L., Guck, J., Kunda, P., and Baum, B. (2012). Changes in Ect2 localization couple actomyosin-dependent cell shape changes to mitotic progression. Dev. Cell *23*, 371–383.

35. Steigemann, P., and Gerlich, D.W. (2009). Cytokinetic abscission: cellular dynamics at the midbody. Trends Cell Biol. *19*, 606–616.

36. Valon, L., Marín-Llauradó, A., Wyatt, T., Charras, G., and Trepat, X. (2017). Optogenetic control of cellular forces and mechanotransduction. Nat. Commun. *8*, 14396.

37. Schindelin, J., Arganda-Carreras, I., Frise, E., Kaynig, V., Longair, M., Pietzsch, T., Preibisch, S., Rueden, C., Saalfeld, S., Schmid, B., et al. (2012). Fiji: an open-source platform for biological-image analysis. Nat. Methods *9*, 676–682.

38. Carpi, N., Piel, M., Azioune, A., Cuvelier, D., and Fink, J. (2011). Micropatterning on silicon elastomer (PDMS) with deep UVs. https://protocolexchange.researchsquare.com/article/nprot-2131/v1.

39. Le Berre, M., Aubertin, J., and Piel, M. (2012). Fine control of nuclear confinement identifies a threshold deformation leading to lamina rupture and induction of specific genes. Integr. Biol. *4*, 1406–1414.

STAR★METHODS

KEY RESOURCES TABLE

REAGENT or RESOURCE	SOURCE	IDENTIFIER
Antibodies		
Mouse monoclonal anti-α-tubulin	Sigma-Aldrich	Cat#T9026; RRID: AB_477593
Mouse monoclonal anti-α-tubulin−FITC	Sigma-Aldrich	Cat#F2168; RRID: AB_476967
Chick anti-GFP	abcam	Cat#ab13970; RRID: AB_300798
Rabbit anti-NuMA	abcam	Cat#ab84680; RRID: AB_2154610
Mouse monoclonal $G_{\alpha i1}$	Santa Cruz Biotechnology	Cat#sc-13533; RRID: AB_2111358
Goat anti-mouse secondary antibody, Alexa Fluor 488	ThermoFisher	Cat#A-11001; RRID: AB_2534069
Goat anti-chick secondary antibody, Alexa Fluor 488	ThermoFisher	Cat#A-11039; RRID: AB_2534096
Goat anti-mouse secondary antibody, Alexa Fluor 546	ThermoFisher	Cat#A-11030; RRID: AB_2534089
Goat anti-rabbit secondary antibody, Alexa Fluor 546	ThermoFisher	Cat#A-11035; RRID: AB_2534093
Goat anti-mouse secondary antibody, Alexa Fluor 647	ThermoFisher	Cat#A-21241; RRID: AB_2535810
Goat anti-mouse secondary antibody, Alexa Fluor 405	ThermoFisher	Cat#A-31553; RRID: AB_221604
Chemicals, Peptides, and Recombinant Proteins		
DMEM GlutaMAX	ThermoFisher	Cat#10566016
Fetal Bovine Serum (FBS)	ThermoFisher	Cat#16000044
Penicillin-Streptomycin	ThermoFisher	Cat#15070063
G418	Sigma-Aldrich	Cat#345810
Puromycin	Sigma-Aldrich	Cat#P8833
FuGENE HD	Promega	Cat#E2311
Lipofectamine LTX Reagent with PLUS Reagent	ThermoFisher	Cat#15338030
Fibronectin	Sigma-Aldrich	Cat#F1141
Lipofectamine 2000	ThermoFisher	Cat#11668030
Latrunculin B	Sigma-Aldrich	Cat#428020
Nocodazole	Sigma-Aldrich	Cat#487929
Y-27632	Sigma-Aldrich	Cat#Y0503
Importazole	Sigma-Aldrich	Cat#SML0341
S-trityl-L-cysteine (STLC)	Sigma-Aldrich	Cat#164739
PLL-g-PEG	SuSOS	Cat#pll20-g3-5-peg2
Fibrinogen-Alexa Fluor 647	ThermoFisher	Cat#F35200
Trypsin-EDTA	ThermoFisher	Cat#R001100
Formaldehyde	TAAB	Cat#F017
Triton X-100	Sigma-Aldrich	Cat#T8787
FluorSave	Sigma-Aldrich	Cat#345789
Phalloidin-TRITC	Sigma-Aldrich	Cat#P1951
DAPI	Sigma-Aldrich	Cat#D9542
Experimental Models: Cell Lines		
Human: HeLa Kyoto cells	[35]	N/A
Human: HeLa H2B-mRFP/tubulin-GFP	[35]	N/A
Human: HeLa GFP-LGN	[12]	N/A
Human: HeLa DHC-GFP	[12]	N/A
Oligonucleotides		
siRNAs against LGN (GAACUAACAGCACGACUUA)	[12]	N/A
Recombinant DNA		
pRK5-Rap1[Q63E] (Rap1*)	[25]	N/A
pmCherry-α-tubulin-IRES-puro2	[35]	N/A

(*Continued on next page*)

Continued

REAGENT or RESOURCE	SOURCE	IDENTIFIER
H2B-mCherry	addgene	20972
lifeact-iRFP	[36]	N/A
Software and Algorithms		
Volocity	Quorum Technologies	https://www.quorumtechnologies.com/volocity
Fiji	[37]	https://fiji.sc/
Custom Python 3 analysis scripts	This paper	https://github.com/andimi/spindle-orientation
Custom Python 3 modeling scripts	This paper	https://github.com/salbreux/Spindle
Other		
12-well glass-bottom plates	MatTek	Cat#P12G-1.5-14-F
35 mm glass-bottom dishes	MatTek	Cat#P35G-1.5-14-C
4-well Lab-Tek II Chamber Slide	Sigma-Aldrich	C6807
Axiovert 200M	Zeiss	N/A
Observer Z1	Zeiss	N/A
Eclipse Ti	Nikon	N/A
Retiga EXi camera	QImaging	N/A
UltraView VOX	Perkin Elmer	N/A
ImagEM camera	Hamamatsu	N/A
TCS SPE laser scanning confocal microscope	Leica	N/A

RESOURCE AVAILABILITY

Lead Contact

Further information and requests for resources and reagents should be directed to and will be fulfilled by the Lead Contact, Buzz Baum (b.baum@ucl.ac.uk).

Materials Availability

There are no restrictions on any data or materials presented in this paper.

Data and Code Availability

Data and Code are available at https://github.com/andimi/spindle-orientation and https://github.com/salbreux/Spindle

EXPERIMENTAL MODEL AND SUBJECT DETAILS

Unlabeled HeLa Kyoto cells and HeLa stable cell lines expressing H2B-mRFP/tubulin-GFP [35], GFP-LGN and DHC-GFP [12], were cultured under standard conditions. They were maintained in Dulbecco's Modified Eagles Medium (DMEM GlutaMAX; ThermoFisher) supplemented with 10% FBS (ThermoFisher) and 50 U/ml penicillin and 50 μg/ml streptomycin (ThermoFisher) at 37 °C under 5% CO_2. Where appropriate, medium was supplemented with selective antibiotics, 0.64 mg/ml G418 (Sigma-Aldrich) and 0.5 μg/ml puromycin (Sigma-Aldrich).

METHOD DETAILS

DNA and siRNA transfection

HeLa cells were transfected with pRK5-Rap1[Q63E] (Rap1* throughout this text) [25], pmCherry-α-tubulin-IRES-puro2 (tubulin-mCherry) [35], H2B-mCherry (addgene plasmid #20972), or lifeact-iRFP [36] using FuGENE HD (Promega), or Lipofectamine LTX with Plus reagent (ThermoFisher), according to the manufacturers' instructions. 20,000 HeLa cells were plated in 12-well plates, in 35 mm glass-bottom dishes (MatTek), in 4-well Lab-Tek (Sigma-Aldrich), or on 10 mm coverslips coated with 10 μg/ml fibronectin (Sigma-Aldrich). The following day, the culture medium was changed for DMEM supplemented with 10% FBS without antibiotics. For transfections with Fugene HD or Lipofectamine LTX with Plus reagent, cells were processed for microscopy 24 hours later to allow expression of the plasmids. For transfection in larger culture dishes, the procedure was scaled appropriately. Control transfection reactions were performed in the absence of plasmid DNA. Cells transfected with Rap1* were identified by their failure to round up in mitosis. HeLa cells were transfected with siRNAs against LGN (GAACUAACAGCACGACUUA) as in [12], using Lipofectamine 2000 (Invitrogen) as previously described [24]. Cells were processed for microscopy after 48 hours. Where RNAi was performed

in conjunction with Rap1* expression, cells were first transfected with siRNAs and sequentially transfected with the Rap1* plasmid for the final 24 hours.

Drug treatments

Cells were treated with 5 μM latrunculin B (Sigma-Aldrich), 10 ng/ml (low doses) or 200 ng/ml (high doses) nocodazole (Sigma-Aldrich), 10 μM Y27632 (Sigma-Aldrich), 40 μM importazole (SigmaAldrich), 5 μM S-trityl-L-cysteine (STLC; Sigma-Aldrich), and, where indicated, control treatments were performed with an equivalent volume of the solvent DMSO.

Micropatterning and Cell Confinement

Micropatterned islands of fibronectin were fabricated with deep UV light [38] on 25 mm coverslips. Glass-bottom dishes were coated with non-adhesive polyethylene glycol, PLL-g-PEG (SuSOS, Switzerland) for 1 h, before deep UV illumination through a photomask. Then, a 25 µg/ml fibronectin solution (Sigma-Aldrich) together with Alexa Fluor 405 or Fibrinogen-Alexa Fluor 647 (ThermoFisher) were incubated for 1 h at room temperature. HeLa cells expressing Rap1* were trypsinised (using Trypsin-EDTA; ThermoFisher), re-suspended in medium at a density of 60,000 cells/ml, seeded onto micropatterned glass-bottomed dishes, and incubated at 37 °C under 5% CO_2 for 1 hr. After 1 hr, the cells were washed in fresh medium and incubated for 4 h to allow spreading on patterned fibronectin before they were processed for microscopy.

For confinement assays, cells were seeded as above on glass-bottom 6-well plates, either on fibronectin-coated, or on PLL-g-PEG-coated glass-bottom dishes. The next day, cells were confined in a defined space (5 µm) as previously described [24, 39]. Briefly, micropillar spacers of the desired height were molded onto a thin layer of PDMS coating 10-mm-diameter glass coverslips. Pillars were coated with either (adhesive) fibronectin, or (anti-adhesive) PLL-g-PEG. Then, these pillars were positioned onto the cells, confining them with sub-micron homogeneity.

Live-cell microscopy

For live-cell imaging, cells were seeded on glass-bottomed dishes (MatTek) coated with 10 mg/ml fibronectin (Sigma-Aldrich), or on micropatterned fibronectin. For mitotic timing experiments, cells were imaged every 2, 3, or 5 min. For live cell microscopy, cells were imaged with a Zeiss Axiovert 200M or Observer Z1 or Nikon Eclipse Ti microscope with a 20X objective (numerical aperture, NA 0.5) or 10X objective (NA 0.3) or 40X oil objective (NA 1.3) equipped with temperature and CO_2 controlling environmental chambers and images acquired using a Retiga EXi camera (Qimaging) and Volocity software (Perkin Elmer). For live cell confocal microscopy, cells were imaged using an UltraView VOX (Perkin Elmer) spinning disc confocal microscope with a 40X (NA 0.75) air objective or 60X (NA 1.4) oil objective equipped with temperature and CO_2 controlling environmental chambers, and images were acquired using a Hamamatsu ImagEM camera and Volocity software (Perkin Elmer).

Immunofluorescence and antibodies

For immunofluorescence, cells on fibronectin-coated glass coverslips were fixed with 4% formaldehyde (TAAB), permeabilized with 0.5% Triton X-100 (Sigma-Aldrich) in PBS for 5 min, and blocked with 5% bovine serum albumin (Sigma-Aldrich) in PBS for 30 min. The cells were sequentially incubated with primary and fluorescently labeled secondary antibodies for 1 h at room temperature and then washed in PBS, 0.1% Triton X-100. The cells were mounted in FluorSave (Sigma-Aldrich) and images were acquired using a Leica TCS SPE laser scanning confocal microscope system.

For immunofluorescence, primary antibodies were used at the following dilutions: α-tubulin 1:200 (mouse monoclonal DM1A; Sigma-Aldrich); FITC-conjugated α-tubulin 1:500 (mouse monoclonal DM1A; Sigma-Aldrich); GFP (chick; abcam); NuMA (rabbit; abcam); $G_{\alpha i1}$ (mouse monoclonal; Santa Cruz Biotechnology). Secondary anti-mouse, anti-rabbit, or anti-chick IgG antibodies (ThermoFisher) tagged with Alexa Fluor 488, 546 or 647 were used at 1:500. Actin was visualized with TRITC-conjugated phalloidin at 1 μg/ml (Sigma-Aldrich), and DNA with DAPI at 1 μg/ml (Sigma-Aldrich).

QUANTIFICATION AND STATISTICAL ANALYSIS

Quantification

Images were processed using Fiji/ImageJ [37], and, where necessary, contrast/brightness was changed uniformly (linearly) across the field. To measure cell length and height, the x, y, and z scales of microscopes were calibrated using 19.28 ± 0.3 mm beads coated with fluorescent PLL-g- PEG by soaking for 30 min in a 0.5 mg/ml PLL-g-PEG solution in 10 mM HEPES pH 7.4 after plasma activation. Measurements and analyses (e.g., cell structure dimensions, distances between elements within the cell, etc) were performed either by using Fiji/ImageJ built-in functions, or custom Python scripts (https://github.com/andimi/spindle-orientation). Centrosome positions were manually tracked, and DNA outlines were manually or semi-automatically traced, and then fitted to an ellipse to extract geometrical parameters. The LGN intensity used in the analysis was normalized by subtracting the background signal outside the cell, and by dividing the resulting intensity by the average intensity calculated in the cell, both in space and time. For the graphical representation of LGN kymographs shown in Figure S3, LGN intensity values were normalized by the average intensity in space, frame-by-frame.

Statistical analysis and visualization

Two sample t test and Mann-Whitney U test were implemented using the scipy library in Python, to compare the mean or median of data from controls and experiments. When samples were assumed to be drawn from a normal distribution, the t test was used, and the Mann-Whitney U test otherwise. The statistical details of experiments can be found in the Figure legends and in the Results section. Unless otherwise stated, n represents the number of cells. Significance was defined as p values < 0.05, and the following notation was used in the Figures: "*" for $0.01 < p$ values < 0.05; "**" for $0.001 < p$ values < 0.01; and "***" for p values < 0.001. The plotnine module (implementing the ggplot R library in python), or the matplotlib/seaborn modules were used for data visualisation. Box and whisker plots show median, upper (75th percentile), and lower (25th percentile) quartiles as the box; whiskers represent the range of the data above the 75th percentile and below the 25th percentile and extend up to 1.5 times the interquartile distance (the difference between the 75th and 25th percentiles). All data points were included in the statistical analysis and in the plots.

Current Biology, Volume *30*

Supplemental Information

Mechanochemical Crosstalk Produces Cell-Intrinsic Patterning of the Cortex to Orient the Mitotic Spindle

Andrea Dimitracopoulos, Pragya Srivastava, Agathe Chaigne, Zaw Win, Roie Shlomovitz, Oscar M. Lancaster, Maël Le Berre, Matthieu Piel, Kristian Franze, Guillaume Salbreux, and Buzz Baum

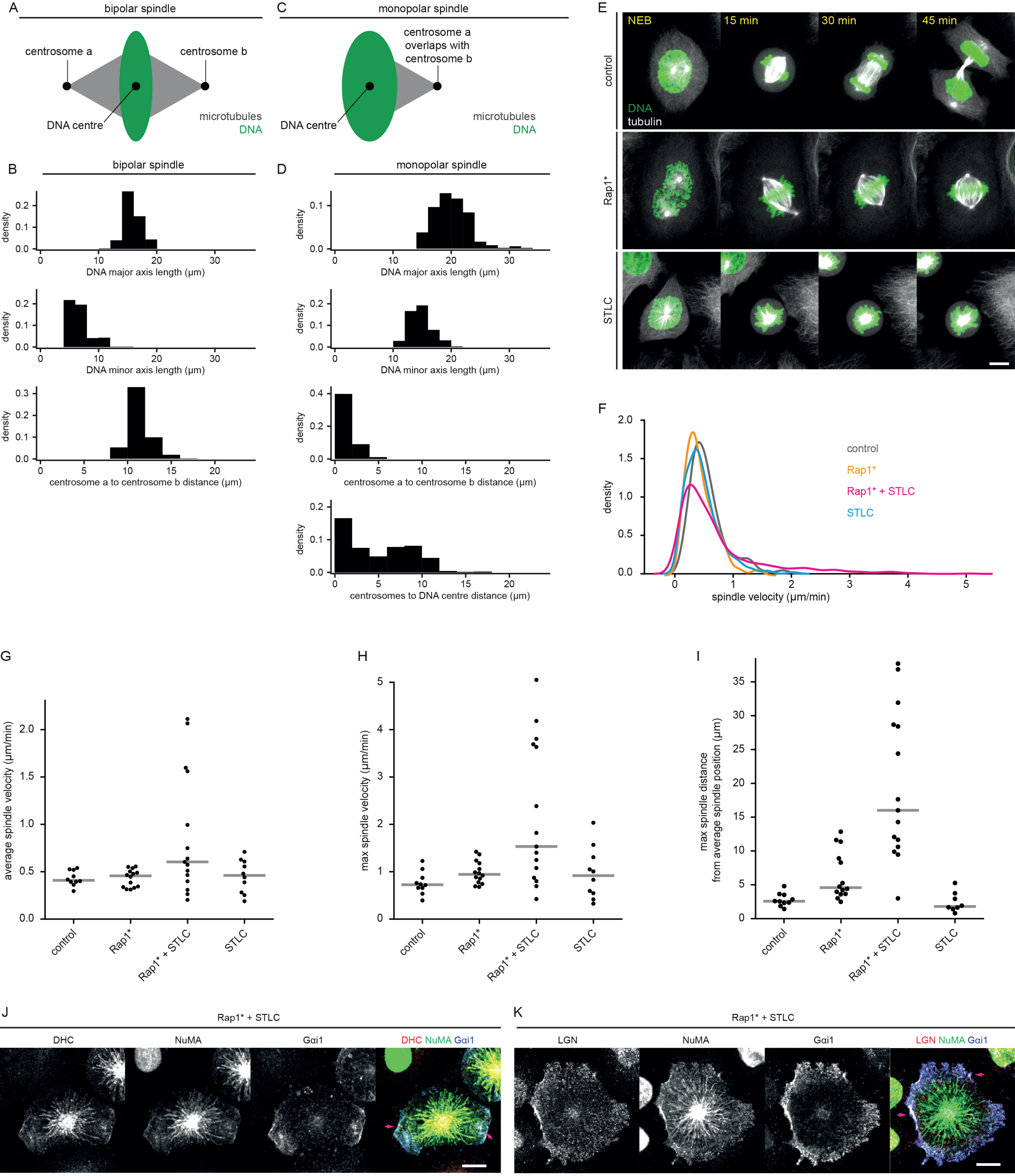
A
bipolar spindle
centrosome a
centrosome b
DNA centre
microtubules
DNA
C
monopolar spindle
centrosome a overlaps with centrosome b
DNA centre
microtubules
DNA
B
bipolar spindle
density
DNA major axis length (μm)
DNA minor axis length (μm)
centrosome a to centrosome b distance (μm)
D
monopolar spindle
DNA major axis length (μm)
DNA minor axis length (μm)
centrosome a to centrosome b distance (μm)
centrosomes to DNA centre distance (μm)
E
NEB
15 min
30 min
45 min
control
Rap1*
STLC
DNA
tubulin
F
control
Rap1*
Rap1* + STLC
STLC
density
spindle velocity (μm/min)
G
average spindle velocity (μm/min)
control
Rap1*
Rap1* + STLC
STLC
H
max spindle velocity (μm/min)
I
max spindle distance from average spindle position (μm)
J
Rap1* + STLC
DHC
NuMA
Gαi1
DHC NuMA Gαi1
K
Rap1* + STLC
LGN
NuMA
Gαi1
LGN NuMA Gαi1

Figure S1 – The geometrical parameters of bipolar and monopolar spindles, and spindle movements in cells treated with different combinations of Rap1* and STLC. Related to Figure 1.

A – Schematic representing a fully formed bipolar spindle (15 min or longer after NEB) under control condition.

B – Histograms of the geometrical parameters (DNA major/minor axis length, and centrosome-to-centrosome distance) that characterise a fully formed bipolar spindle (15 min or longer after NEB) in cells on FN-coated unpatterned substrates (as in **A**).

C – Schematic representing a monopolar spindle (in Rap1* +STLC treated cells, where centrosomes fail to separate).

D – Histograms of the geometrical parameters (DNA major/minor axis length, centrosome-to-centrosome distance, and centrosome-to-DNA distance) that characterise a monopolar spindle in cells on FN-coated unpatterned substrates (as in **C**).

E –Time-lapse confocal images of representative HeLa cells on FN-coated unpatterned substrates in 3 conditions as they enter mitosis: (top) control, (middle) Rap1*, and (bottom) STLC. Cells are expressing tubulin-GFP and H2B-mCherry. The scalebar indicates 10 µm.

F – Density plots of spindle velocities in cells on FN-coated unpatterned substrates in 4 different conditions: control, Rap1*, Rap1* + STLC, and STLC.

G – Plot showing average spindle velocities for cells used in **F**.

H – Plot showing maximum spindle velocities for cells used in **F**.

I – Plot showing the maximum distance between the spindle at any time during mitosis from its average position in the 4 conditions (as in **F**).

J, K – Immuno-fluorescence confocal images of HeLa cells in mitosis (Rap1* + STLC) on FN-coated unpatterned substrates. **J**. Dynein (DHC) and NuMA are found localised to spindle microtubules and the cell cortex far from the spindle - where Gαi1 also localises. **K**. NuMA is found localised to spindle microtubules and the cell cortex - where LGN and Gαi1 also localise.

Regions where the three proteins overlap are shown in white in the overlay and highlighted by magenta arrows.

All scalebars indicates 10 µm. In **G**, **H**, and **I** grey bars indicate median values.

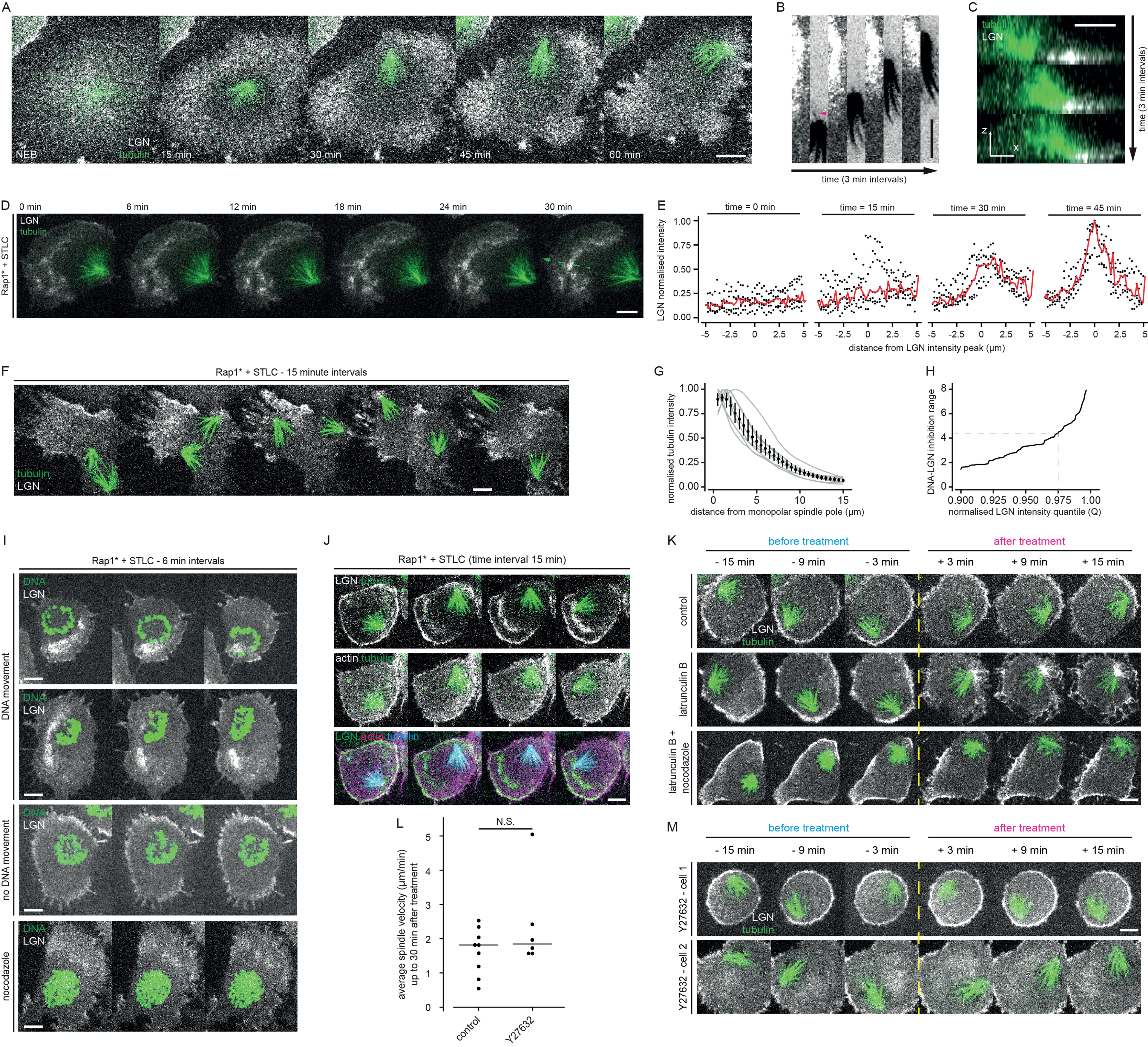
A
LGN
tubulin
NEB
15 min
30 min
45 min
60 min
B
time (3 min intervals)
C
tubulin
LGN
time (3 min intervals)
z
x
D
0 min
6 min
12 min
18 min
24 min
30 min
Rap1* + STLC
LGN
tubulin
E
time = 0 min
time = 15 min
time = 30 min
time = 45 min
LGN normalised intensity
distance from LGN intensity peak (µm)
F
Rap1* + STLC - 15 minute intervals
tubulin
LGN
G
normalised tubulin intensity
distance from monopolar spindle pole (µm)
H
DNA-LGN inhibition range
normalised LGN intensity quantile (Q)
I
Rap1* + STLC - 6 min intervals
DNA movement
no DNA movement
nocodazole
DNA
LGN
J
Rap1* + STLC (time interval 15 min)
LGN tubulin
actin tubulin
LGN actin tubulin
K
before treatment
after treatment
- 15 min
- 9 min
- 3 min
+ 3 min
+ 9 min
+ 15 min
control
latrunculin B
latrunculin B + nocodazole
LGN
tubulin
L
N.S.
average spindle velocity (µm/min) up to 30 min after treatment
control
Y27632
M
before treatment
after treatment
Y27632 - cell 1
Y27632 - cell 2

Figure S2 – LGN dynamics in flat mitotic cells with a monopolar spindle, and the role of the actomyosin cortex in monopolar spindle movement and LGN cortical stability. Related to Figure 2.

A – After NEB, LGN is inhibited near the spindle and accumulates far from the spindle. Time-lapse images of a mitotic HeLa cell (Rap1* + STLC) on a FN-coated unpatterned substrate (confocal imaging), showing dynamics of LGN accumulation and inhibition as a monopolar spindle moves across the cell.

B – Detail of a monopolar spindle in a cell on a FN-coated unpatterned substrate following high levels of LGN (left, high levels in white), which in turn are depleted as the spindle approaches (right, microtubules in black). A magenta arrow points at astral-microtubules emanating from the spindle pole towards a patch of LGN.

C – X-Z section of a confocal time-lapse of a representative HeLa cell on a FN-coated unpatterned substrate treated with Rap1* + STLC. Centrosomes lie close to the basal membrane where LGN is concentrated.

D – Representative confocal time-lapse of a HeLa cell on a FN-coated unpatterned substrate with a monopolar spindle that stopped moving. LGN accumulates in regions far from the spindle but does not diffuse. The green dashed line indicates a region of interest used in **E**

E – Plots of LGN normalised intensity in cells where monopolar spindles stopped moving and allowed for monitoring the accumulation of LGN in space and time. The horizontal axis represents the distance from a peak of LGN (e.g. the green dashed line in **D**). Over time LGN increases in specific regions of the cell but does not diffuse rapidly (n = 5). Red lines indicate median values.

F – Confocal time-lapse of a HeLa cell on a FN-coated unpatterned substrate with a bipolar spindle that breaks up into 2 monopolar spindles that move across the cell. Cycles of LGN clearance and accumulation are locally coordinated with both monopolar spindles.

G – Plot of the normalised tubulin intensity as a function of the distance from the spindle pole in HeLa cells (as shown in Figure 2C-D, n = 8). Black dots and bars represent mean and standard error from bootstrap respectively.

H – Plot of the DNA's mean LGN inhibition range (n = 29), as a function of the threshold (corresponding to a specific quantile of the normalised LGN intensity distribution) used to

define “high levels of LGN” (see Methods S1). The threshold (0.975) and corresponding mean inhibition range are indicated by dashed green lines. The corresponding full histogram of the DNA’s LGN inhibition range is shown in Figure 2G.

I – Time-lapse confocal images of representative HeLa cells treated with Rap1* + STLC (monopolar flat) in 3 conditions: (top two rows) no additional treatment with DNA moving across the cell, (middle) no additional treatment with no DNA movements across the cell, and (bottom) additional nocodazole treatment. Cells are expressing tubulin-GFP and H2B-mCherry.

J – Confocal time-lapse of a monopolar spindle (top and middle montage, green, and bottom montage, cyan) moving in a representative HeLa cells on FN-coated unpatterned substrates. LGN (top montage, grey, and bottom montage, green) undergoes cycles of accumulation and clearance that are tightly coordinated with spindle position, while actin (middle montage, grey, and bottom montage, magenta) does not.

K – Confocal time-lapses of monopolar spindles in cells on FN-coated unpatterned substrates before and after treatments perturbing the actin cortex. Compared to the control treatment (top), latrunculin B treatment (middle) leads to a large relocalisation of LGN from the cortex to the centrosome. This accumulation is astral-microtubule dependent, as it is prevented by simultaneously treating cells with latrunculin B and low doses of nocodazole (bottom).

L – Average spindle velocities in cells treated with Y27632 (as in **M**) are similar to control cells (p-value > 0.05, Mann-Whitney *U* test). Grey bars represent median values.

M – Confocal time-lapses of monopolar spindles in 2 representative HeLa cells on FN-coated unpatterned substrates before and after Y27632 treatment. Spindle velocity is not affected by ROCK inhibition.

All scalebars indicates 10 µm

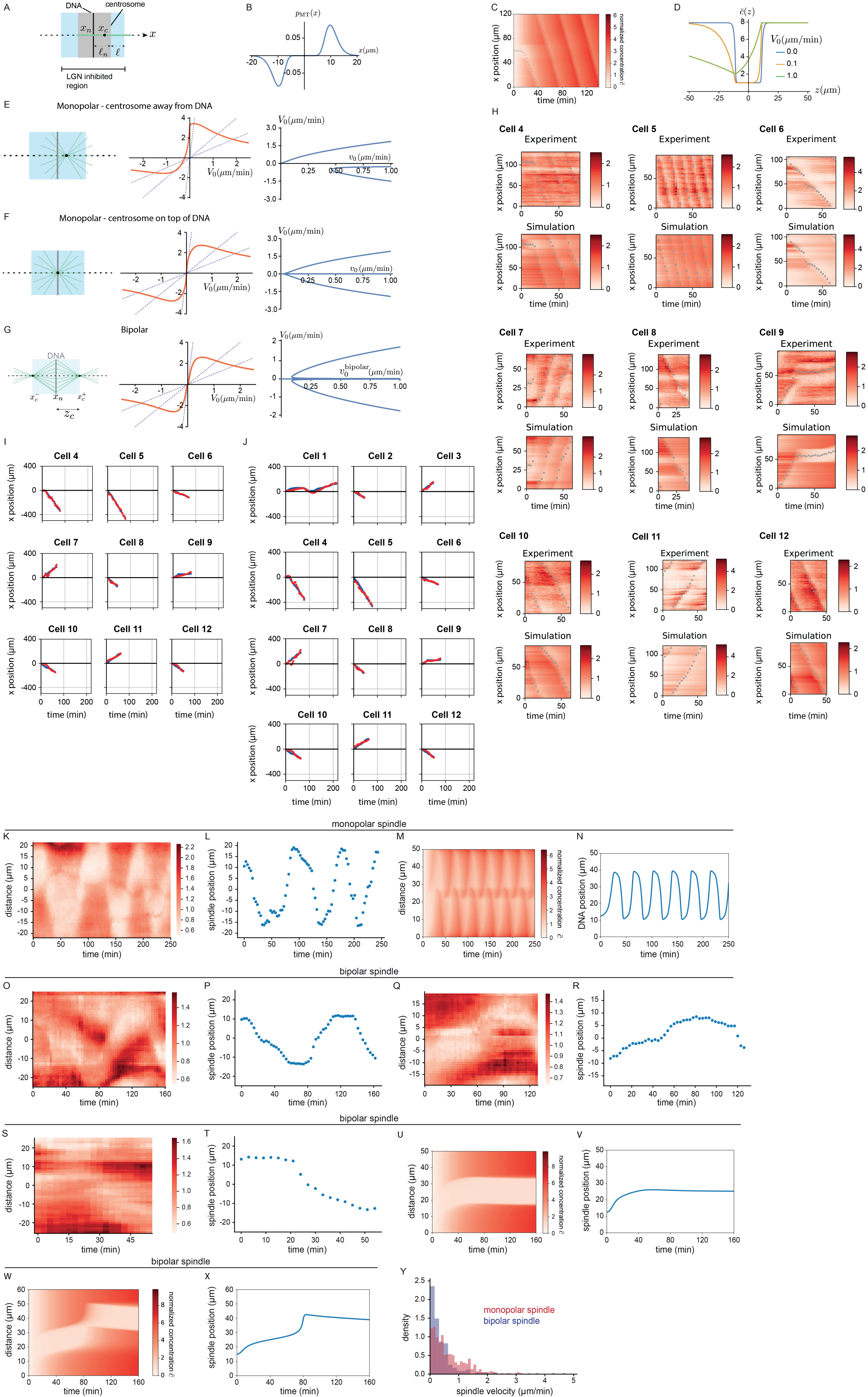

A
DNA
centrosome
x_n
x_c
ℓ_n
ℓ
x
LGN inhibited region
B
$p_{\mathrm{MT}}(x)$
$x(\mu m)$
C
x position (μm)
time (min)
normalized concentration $\bar{c}$
D
$\bar{c}(z)$
$V_0(\mu m/\mathrm{min})$
0.0
0.1
1.0
$z(\mu m)$
E
Monopolar - centrosome away from DNA
$V_0(\mu m/\mathrm{min})$
$v_0(\mu m/\mathrm{min})$
F
Monopolar - centrosome on top of DNA
$V_0(\mu m/\mathrm{min})$
$v_0(\mu m/\mathrm{min})$
G
Bipolar
DNA
x_c^-
x_n
x_c^+
z_c
$V_0(\mu m/\mathrm{min})$
$v_0^{\mathrm{bipolar}}(\mu m/\mathrm{min})$
H
Cell 4
Cell 5
Cell 6
Cell 7
Cell 8
Cell 9
Cell 10
Cell 11
Cell 12
Experiment
Simulation
x position (μm)
time (min)
I
J
Cell 1
Cell 2
Cell 3
x position (μm)
time (min)
monopolar spindle
K
L
M
N
distance (μm)
spindle position (μm)
DNA position (μm)
normalized concentration $\bar{c}$
time (min)
bipolar spindle
O
P
Q
R
bipolar spindle
S
T
U
V
bipolar spindle
W
X
Y
density
monopolar spindle
bipolar spindle
spindle velocity (μm/min)

Figure S3 – Analysis of the one-dimensional model of monopolar and bipolar spindle motion and LGN concentration in cells on unpatterned substrates and on patterned lines. Related to Figure 3.

A – Schematic of geometrical quantities in the one-dimensional model of spindle motion. Black line: DNA, black dot: centrosome, green line: microtubules emanating from the centrosome. x_n is the position of the DNA, x_c the position of the centrosome, ℓ_n half the width of the DNA, and ℓ is the distance away from the DNA up to which the cortical LGN unbinding rate is enhanced.

B – Signed distribution of astral microtubule end density, obtained from a fit to experimental data (Figure 2D). The sign of the distribution corresponds to the orientation of microtubules.

C – LGN concentration and spindle motion in the full mechano-chemical feedback model, when the centrosome and DNA centre are at the same location ($x_c = x_n$). Colours are as in Figure 3E. The initial condition for the concentration profile is homogeneous with a small random perturbation. A symmetry-breaking instability leads to spontaneous motion of the DNA.

D – Steady-state profiles of theoretical LGN concentration, for positions z relative to the DNA, at different DNA velocities and for an infinite domain (see Methods S1). Different colours correspond to different velocities of the DNA (blue: $V_0 = 0$, orange: $V_0 = 0.1\ \mu\text{m/min}$, green: $V_0 = 1\ \mu\text{m/min}$). Other parameters are listed in Table I in Methods S1. DNA movement is assumed to be directed towards positive values of z. As the DNA velocity is increased, a region of inhibited LGN concentration extends behind the DNA.

E – Left: schematic of centrosome, DNA and microtubule organization, when the centrosome and DNA have different positions. Middle: a self-consistent equation (Eq. 15 in Methods S1) determines the DNA-centrosome velocity V_0 at the intersection between the red curve and blue lines (corresponding to v_0=0.05 $\mu\text{m/min}$, v_0=0.5 $\mu\text{m/min}$, v_0=1 $\mu\text{m/min}$, from the largest to smallest slope). Right: DNA velocity V_0 as a function of the characteristic velocity v_0, as determined from the self-consistent equation. A moving solution exists for all non-zero values of v_0, with the velocity of the DNA oriented towards the centrosome. For high enough values of v_0, two other solutions emerge with an opposite orientation of velocity.

F – Left: schematic of centrosome, DNA and microtubule organization, when the centrosome and DNA are at the same position. Middle: A self-consistent equation determines the DNA-centrosome velocity V_0 at the intersection between the red curve and blue lines (corresponding to v_0=0.05 $\mu\mathrm{m/min}$, v_0=0.5 $\mu\mathrm{m/min}$, v_0=1 $\mu\mathrm{m/min}$, from the largest to smallest slope). Right: DNA velocity V_0 as a function of the characteristic velocity v_0, as determined from the self-consistent equation. For a large enough value of v_0, two moving solutions with velocities in opposite direction emerge. In our analysis of monopolar spindle, we obtain $v_0 = 6.7 \pm 6.7\ \mu\mathrm{m/min}$, with an average value above the threshold for spontaneous motion.

G – Left: schematic of centrosome, DNA and microtubule organization, for a bipolar spindle. Middle: A self-consistent equation determines the bipolar spindle velocity V_0 at the intersection between the red curve and blue lines (corresponding to v_0^{bipolar}=0.05 $\mu\mathrm{m/min}$, v_0^{bipolar}=0.5 $\mu\mathrm{m/min}$, v_0^{bipolar}=1 $\mu\mathrm{m/min}$, from the largest to smallest slope). Right: bipolar spindle velocity V_0 as a function of the characteristic velocity v_0^{bipolar}, as determined from the self-consistent equation. For a large enough value of v_0^{bipolar}, moving solutions with velocities in opposite direction emerge.

H – Top panels, experimental kymographs showing the cortical LGN fluorescence intensity over space and time (red colours), and the DNA position over time (grey dots) for 9 cells analysed, in addition to 3 cells shown in Figure 3B. Bottom panels, corresponding kymographs of simulated cortical LGN concentration, obtained by imposing the experimentally measured DNA position.

I – Distance travelled by the DNA, experimentally measured (blue) and predicted from the equation given in Figure 3C, for 9 analysed cells, in addition to 3 cells shown in Figure 3D. The value of the proportionality coefficient v_0 is chosen independently for each cell.

J – As in **I**, distance travelled by the DNA, experimentally measured (blue) and predicted from the equation given in Figure 3C, for all 12 analysed cells, but for a different choice of distribution of force acting on microtubules. Instead of assuming that forces act on the end of microtubules, as in **I**, one assumes here that forces act on the entire microtubule length (see Methods S1).

K – Kymograph (space-time representation) of LGN concentration over time in a HeLa cell treated with Rap1* + STLC (with a monopolar spindle) on a line pattern.

L – Position over time of the monopolar spindle in the HeLa cell shown in **K.**

M – Kymograph of simulated cortical LGN concentration, with no-flux boundary conditions for LGN at both ends of the one-dimensional domain, for a monopolar spindle, when the centrosome and DNA centre are at the same location ($x_c = x_n$). Parameters are as given in Table 1, except for the cell size which is $L_c = 50\mu\text{m}$. Initial conditions are set to $\bar{c} = 0$ (no cortical LGN) and the DNA position is offset from the cell centre (initial position $L_c/4$).

N – Position over time of DNA centre for the simulation shown in **M**. The DNA exhibits spontaneous oscillations.

O, Q, S – Kymograph (space-time representation) of LGN concentration over time in a HeLa cell treated with Rap1* (with a bipolar spindle) on a line pattern.

P, R, T – Position over time of the bipolar spindle in the HeLa cell shown in **O, Q, S.**

U, W – Kymograph of simulated cortical LGN concentration, with no-flux boundary conditions for LGN at both ends of the one-dimensional domain, for a bipolar spindle. Parameters are as given in Table 1 or in section 1D-E of the Methods S1, and the domain size is $L_c = 50$ µm (U) or $L_c = 60$ µm (W). Initial conditions are set to $\bar{c} = 0$ (no cortical LGN) and the DNA position is offset from the cell centre (initial position $L_c/4$ with L_c the domain size).

V, X – Position over time of DNA centre for the simulation shown in **U**, **W**. In **V** the spindle converges to the cell centre, while it starts an oscillatory movement in **W**.

Y – Histograms of spindle velocities for monopolar (n = 5 cells) and bipolar (n = 4 cells) spindles in HeLa cells on line patterns (representative plots shown respectively in **L,** and **P-R-T**).

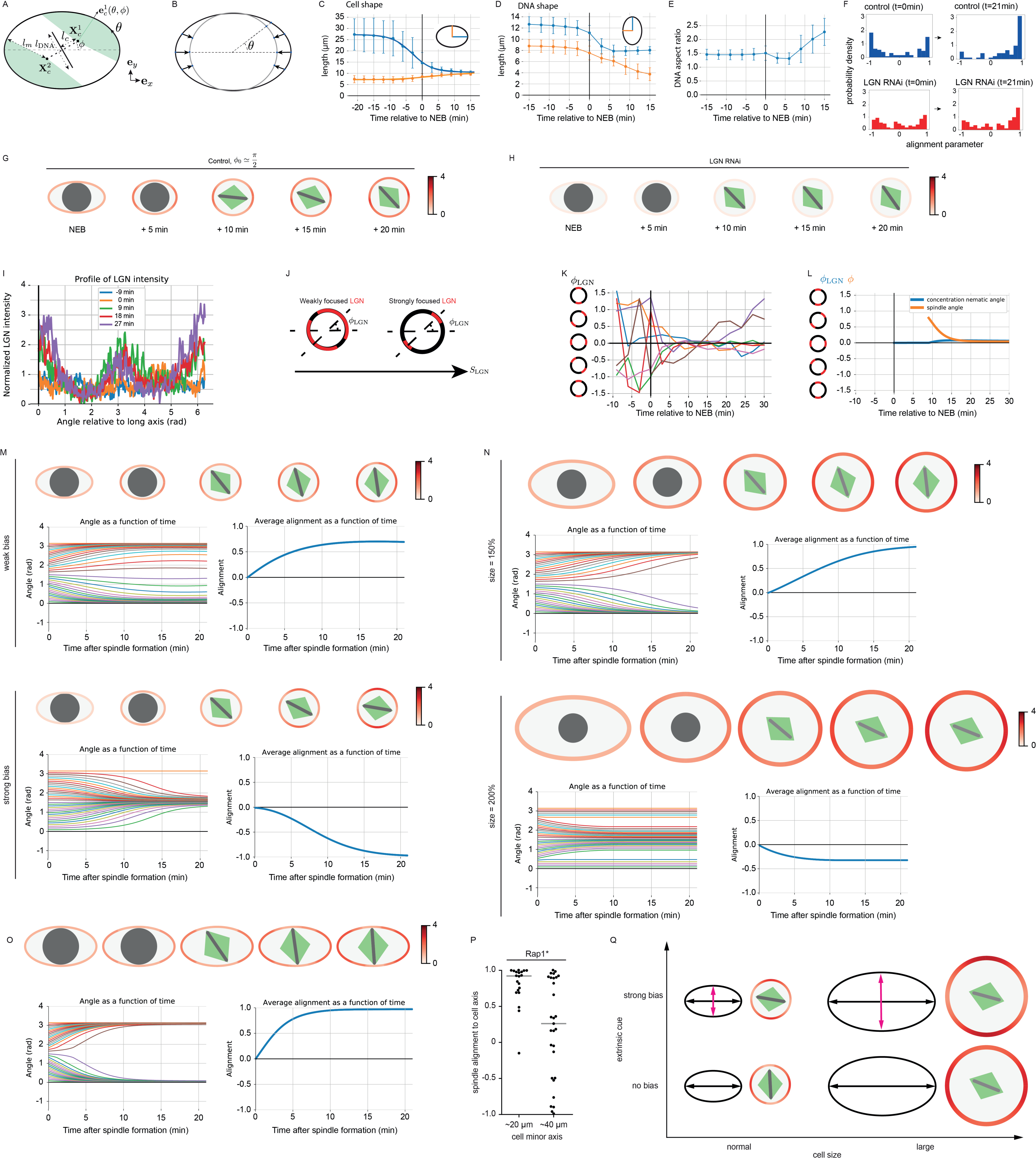

A
B
C
Cell shape
length (μm)
Time relative to NEB (min)
D
DNA shape
length (μm)
Time relative to NEB (min)
E
DNA aspect ratio
Time relative to NEB (min)
F
control (t=0min)
control (t=21min)
LGN RNAi (t=0min)
LGN RNAi (t=21min)
probability density
alignment parameter
G
Control, $\phi_0 \simeq \frac{\pi}{2}$
NEB
+ 5 min
+ 10 min
+ 15 min
+ 20 min
H
LGN RNAi
NEB
+ 5 min
+ 10 min
+ 15 min
+ 20 min
I
Profile of LGN intensity
-9 min
0 min
9 min
18 min
27 min
Normalized LGN intensity
Angle relative to long axis (rad)
J
Weakly focused LGN
Strongly focused LGN
ϕ_{LGN}
S_{LGN}
K
ϕ_{LGN}
Time relative to NEB (min)
L
ϕ_{LGN} ϕ
concentration nematic angle
spindle angle
Time relative to NEB (min)
M
weak bias
Angle as a function of time
Angle (rad)
Time after spindle formation (min)
Average alignment as a function of time
Alignment
Time after spindle formation (min)
strong bias
Angle as a function of time
Angle (rad)
Time after spindle formation (min)
Average alignment as a function of time
Alignment
Time after spindle formation (min)
N
size = 150%
Angle as a function of time
Angle (rad)
Time after spindle formation (min)
Average alignment as a function of time
Alignment
Time after spindle formation (min)
size = 200%
Angle as a function of time
Angle (rad)
Time after spindle formation (min)
Average alignment as a function of time
Alignment
Time after spindle formation (min)
O
Angle as a function of time
Angle (rad)
Time after spindle formation (min)
Average alignment as a function of time
Alignment
Time after spindle formation (min)
P
Rap1*
spindle alignment to cell axis
~20 μm
~40 μm
cell minor axis
Q
strong bias
no bias
extrinsic cue
normal
large
cell size

Figure S4 – Two-dimensional model of bipolar spindle motion and LGN cortical distribution in simulations and experiments, and the effect of bias of cortical cues and cell size on the model. Related to Figure 4.

A – Schematic of geometrical parameters in a two-dimensional model of bipolar spindle motion.

B – Schematic of cell rounding. In simulations, we assume for simplicity that points on the cell outline follow trajectories of constant polar angle θ as mitotic rounding occurs.

C – Fit to quantification of cell shape changes during mitotic rounding, for mitotic HeLa cells cultured on FN-coated micropatterned lines. Blue: long cell axis half-length, Orange: short cell axis half-length. Dots: mean of experimental data for n=33 cells, error bars: standard deviation, solid lines: fit to Eqs. 37-38 in Methods S1.

D - DNA shape changes around NEB in HeLa cells on FN-coated unpatterned substrates, as a function of time. Blue: DNA semi long axis length, Orange: DNA semi short axis length. Dots are mean of experimental data for n=44 cells, error bars are standard deviation.

E – DNA aspect ratio around NEB in HeLa cells on FN-coated unpatterned substrates, as a function of time. The aspect ratio is defined as the long axis length divided by the short axis length. Error bars: standard deviation for n=44 cells.

F – Distribution of experimentally measured spindle alignment along the long cell axis in mitotic HeLa cells cultured on micropatterned lines, with either control (n=94) or LGN-RNAi (n=96) treatment, at the time of spindle formation (left) and 21 minutes after spindle formation (right).

G – Cell shape, DNA and spindle angle, and LGN concentration at different times after NEB, in a simulation of mitotic rounding and spindle rotation in control conditions, for an initial spindle angle ϕ away from the long axis close to $\pi/2$ ($\phi_0 = \pi/2$ - $\pi/40$). Simulation time points and colour codes are as in Figure 4I.

H – Cell shape, DNA and spindle angle, and LGN concentration at different times after NEB, in a simulation of mitotic rounding and spindle rotation in LGN-RNAi conditions, for an initial angle $\phi_0 = \pi/4$. Simulation time points and colour codes are as in Figure 4I.

I – Quantification of LGN fluorescence intensity profile on the cell contour, at different times, from one representative cell shown in Figure 4A. Angles are taken relative to the long cell axis measured at time = NEB – 9 minutes.

J – Schematic of nematic order parameter calculated from the distribution of LGN concentration around the cell contour. S_{LGN} is the nematic order magnitude which quantifies how strongly the concentration is focused on opposite poles in the cell (see Methods S1 for a definition). ϕ_{LGN} is the nematic angle which quantifies the orientation of the axis of bipolar distribution of LGN.

K – Nematic angle ϕ_{LGN} as a function of time for different cells (n=7), for which Figure 4A is a representative example.

L – Nematic angle ϕ_{LGN} (blue curve) and spindle angle ϕ (orange curve) as a function of time, for the simulation shown in Figure 4I. In the simulation LGN orients along the long axis after NEB, and a slight loss of LGN orientation occurs after spindle formation, due to the anisotropic shape of DNA and slight misalignment of the spindle.

M – Simulation results with increasing bias along the vertical axis arising from an external cue. A bias is introduced by assuming a spatial profile of LGN binding rate $k_{\mathrm{on}}(\theta)$ with a maximum k_{on}^{+} at $\theta = \pm\frac{\pi}{2}$ and a minimum k_{on}^{-} at $\theta = 0,\ \pi$ (see Methods S1 for a more precise description). Top graphs: weak bias $k_{\mathrm{on}}^{+} \simeq 1.45\ k_{\mathrm{on}}^{-}$; bottom graphs: strong bias $k_{\mathrm{on}}^{+} = 4\ k_{\mathrm{on}}^{-}$. For each case: Top images depict simulation outputs, with time points and colour codes as in Figure 4I; bottom left graphs show predicted spindle orientation trajectories as a function of time, as in Figure 4J; bottom right graphs show the average alignment of the spindle angle with the initial cell elongation axis, as a function of time, as in Figure 4L. A strong enough bias polarizes LGN along the vertical cell axis, leading to a force distribution that orients the spindle axis perpendicular to the cell elongation axis. Negative alignment values correspond to spindle orienting along the vertical axis, with -1 corresponding to perfect vertical alignment.

N – Simulation results with increasing cell size. The cell shape is isotropically scaled by a constant factor (see Methods S1 for a more precise description). Top graphs: cell size is increased by 1.5 compared to Figure 4I; bottom graphs: cell size is increased by 2. For each case: Top images depict simulation outputs, with time points and colour codes as in Figure 4I; bottom left graphs show predicted spindle orientation trajectories as a function

of time, as in Figure 4J; bottom right graphs show the average alignment of the spindle angle with the initial cell elongation axis, as a function of time, as in Figure 4L. When the cell becomes too large, astral microtubules have reduced contacts with the cortex, leading to loss of spindle alignment along the long axis.

O - Simulation result with constant elongated cell shape (see Methods S1 for a more precise description). Top images depict simulation outputs, with time points and colour codes as in Figure 4I; bottom left graph shows predicted spindle orientation trajectories as a function of time, as in Figure 4J; bottom right graph shows the average alignment of the spindle angle with the initial cell elongation axis, as a function of time, as in Figure 4L. In this example the spindle orients on average along the long cell axis.

P – Plot showing the alignment between the mitotic spindle and the cell axis (measured at the timepoint before anaphase onset) in Rap1* cells with a small width (on FN-coated line patterns, minor axis length 22 µm ± 6 µm; major axis length 65 µm ± 17 µm), and with a larger width (on FN-coated unpatterned substrates, minor axis length 37 µm ± 7 µm; major axis length 59 µm ± 10 µm). Grey bars represent median values.

Q – Schematic of the effect of the bias of cortical cues and of cell size on spindle alignment with the cell axis. Black ellipses show interphase cells shape, black lines and arrows show the interphase cell axis, and the magenta line and arrows represent the extrinsic cue bias. In control HeLa cells (where weak or no extrinsic bias of cortical cues are present), the spindle aligns to the interphase cell axis through the mechano-chemical interaction between astral-microtubules and cortical motors. When a strong extrinsic cue is present, spindle alignment can be overridden to ignore interphase cell shape. In large cells, microtubules and DNA-mediated inhibition do not scale with cell size, thus the spindle cannot align to the interphase elongation cell axis.
